# Not Frozen in the Ice: Large and Dynamic Rearrangements in the Mitochondrial Genomes of the Antarctic Fish

**DOI:** 10.1093/gbe/evab017

**Published:** 2021-02-11

**Authors:** Chiara Papetti, Massimiliano Babbucci, Agnes Dettai, Andrea Basso, Magnus Lucassen, Lars Harms, Celine Bonillo, Franz Maximilian Heindler, Tomaso Patarnello, Enrico Negrisolo

**Affiliations:** 1 Department of Biology, University of Padova, Padova 35121,Italy; 2 Consorzio Nazionale Interuniversitario per le Scienze del Mare (CoNISMa), Roma 00196, Italy; 3 Department of Comparative Biomedicine and Food Science, University of Padova, Legnaro 35020, Italy; 4 Institut de Systematique, Evolution, Biodiversité (ISYEB) Muséum national d’Histoire naturelle-CNRS-Sorbonne Université-EPHE, MNHN, Paris 75005, France; 5 Helmholtz Centre for Polar and Marine Research, Alfred Wegener Institute, Am Handelshafen 12, Bremerhaven 27570, Germany; 6 Helmholtz Institute for Functional Marine Biodiversity, University of Oldenburg (HIFMB), Ammerlsity of Oldenburg (HIFMOldenburg 26129, Germany; 7 Service de Systématique Moléculaire, UMS2700 Acquisition et Analyse de Données (2AD), MNHN, Paris 75005, France; 8 Laboratory of Biodiversity and Evolutionary Genomics, KU Leuven, Leuven, Belgium; 9 CRIBI Interdepartmental Research Centre for Innovative Biotechnologies, University of Padova, viale G. Colombo 3, Padova 35121, Italy

**Keywords:** mitochondrial genome evolution, gene order rearrangements, Notothenioidei, *Trematomus*, icefish, *Dissostichus*

## Abstract

The vertebrate mitochondrial genomes generally present a typical gene order. Exceptions are uncommon and important to study the genetic mechanisms of gene order rearrangements and their consequences on phylogenetic output and mitochondrial function. Antarctic notothenioid fish carry some peculiar rearrangements of the mitochondrial gene order. In this first systematic study of 28 species, we analyzed known and undescribed mitochondrial genome rearrangements for a total of eight different gene orders within the notothenioid fish. Our reconstructions suggest that transpositions, duplications, and inversion of multiple genes are the most likely mechanisms of rearrangement in notothenioid mitochondrial genomes. In Trematominae, we documented an extremely rare inversion of a large genomic segment of 5,300 bp that partially affected the gene compositional bias but not the phylogenetic output. The genomic region delimited by *nad5* and *trnF*, close to the area of the Control Region, was identified as the hot spot of variation in Antarctic fish mitochondrial genomes. Analyzing the sequence of several intergenic spacers and mapping the arrangements on a newly generated phylogeny showed that the entire history of the Antarctic notothenioids is characterized by multiple, relatively rapid, events of disruption of the gene order. We hypothesized that a pre-existing genomic flexibility of the ancestor of the Antarctic notothenioids may have generated a precondition for gene order rearrangement, and the pressure of purifying selection could have worked for a rapid restoration of the mitochondrial functionality and compactness after each event of rearrangement.

**Significance**

The mitochondrial genomes of the Antarctic notothenioid fish deviate from the canonical vertebrate gene order, as was described for a few species, but the extent of this variability was not known for the whole group. Our study found that more gene order rearrangements than previously known have occurred during the entire history of the Antarctic notothenioid species by multiple events of transposition, duplication, and inversion of several genes. These findings suggest a high flexibility of the notothenioid mitochondrial genome which is unusual for a vertebrate group. The results outline the exceptionality and uniqueness of the notothenioids beyond their widely studied extraordinary array of exclusive adaptations to cold.

## Introduction

**Fig. 1.**
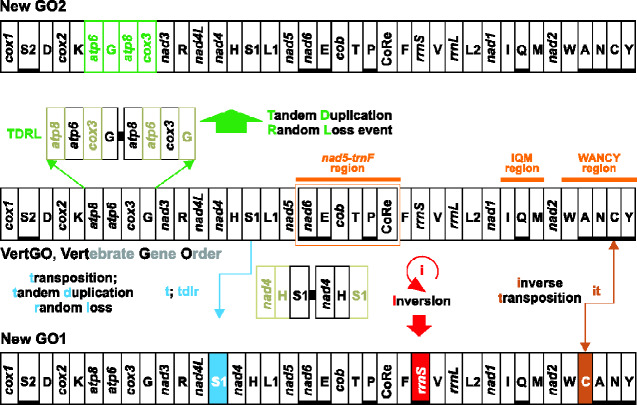
Mechanisms generating novel gene orders (GO). NewGO: hypothetical GO generated by the depicted rearrangements. VertGO is linearized starting from *cox1*. The genes encoded on the H-strand (orientation from left to right) are black-boxed, whereas those encoded on the L-strand (orientation from right to left) are underlined and black-boxed. Nomenclature: *atp6* and *atp8*, ATP synthase subunits 6 and 8; *cob*, apocytochrome b; *cox1-3*, cytochrome c oxidase subunits 1–3; *nad1-6* and *nad4L*, NADH dehydrogenase subunits 1–6 and 4 L; *rrnS* and *rrnL*, small and large subunit ribosomal RNA (rRNA) genes; X, transfer RNA (tRNA) genes, where X is the one-letter abbreviation of the corresponding amino acid, in particular L1 (CTN codon family) L2 (TTR codon family), S1 (AGN codon family) S2 (TCN codon family); CoRe, Control Region; I, inversion; it, inverse transposition; t, transposition; tdrl, tandem duplication random loss mechanism producing the observed rearrangement; TDRL, tandem duplication and random loss event. The extra copy of every gene that is lost in the genomic rearrangement is framed in light green.

The Bilaterian mitochondrial genome (hereafter mitogenome) is usually a double-strand circular DNA molecule spanning 15–20 kb (Boore 1999). Most Bilaterian mitogenomes contain 37 genes of well-known function (fig. 1),that is,13 protein-coding genes (*cox1*–*cox3*, *atp6* and *atp8*, *cob*, *nad1*–*nad6*, and *nad4L*), two ribosomal genes (*rrnS* and *rrnL*), and 22 tRNAs (Boore 1999; Gissi et al. 2008; Bernt et al. 2013). The Bilaterian mitogenomes also contain two replication origins, one for the H-strand (the Control Region, CoRe in fig. 1) and one for the L-strand (O_L_) (Boore 1999, 2000, Bernt et al. 2013). Some Bilaterian mitogenomes contain less than 37 genes owing to the loss of tRNAs or protein-coding genes (Gissi et al. 2008; Bernt et al. 2013), whereas some other organisms carry multiple copies of the same mitochondrial gene/s (Gissi et al. 2008). The 37 mitochondrial genes are differently arranged among Bilaterians. They can all be encoded on the same strand, or on both strands (Boore 1999). Irrespective to their arrangement on the two strands, the Bilaterian mitochondrial genes can be contiguous, overlapping, or separated by intergenic spacers (ISPs) of variable length.

The mitogenomes of Vertebrata exhibit a typical gene order (hereafter VertGO) (fig. 1) that is generally conserved in the lineage (Boore 1999) although mitochondrial gene order rearrangements are not rare and several exceptions to the VertGO have been described. In fish, most species sequenced to date present the VertGO, but several alternative GOs have been found for instance in flatfishes (Shi et al. 2013, 2014), deep-sea gulper eels (Inoue et al. 2003) as also in the Antarctic notothenioids (Zhuang and Cheng 2010; for a review see Satoh et al. 2016). Instances of gene order rearrangement are exceptional opportunities to investigate the genetic mechanisms of gene order rearrangements and the consequences on phylogenetic output and mitochondrial function.

Theoretically, if every gene had an equal probability of movement, the 37 mitochondrial genes could be arranged in an astonishing number of gene order combinations (i.e., 37! or 38!, if CoRe is also included, and excluding gene duplications) (Babbucci et al. 2014; Cameron 2014). However, it is not yet clear why some mitochondrial regions are more prone than others to rearrangement. In fact, the known exceptions to VertGo show that not all the gene order combinations are equally represented. For instance, in fish mitogenomes, hot spots with higher frequency of change have been identified in the CoRe, IQM (*trnI*, *trnQ*, *trnM*), and WANCY (*trnW*, *trnA*, *trnN*, O_L_, *trnC*, *trnY*) regions (fig. 1) (e.g., Inoue et al. 2003; Miya et al. 2003; Satoh et al. 2016). Genes frequently replicated are located close to the replication origin, the CoRe, for functional efficiency and appear to be also more prone to duplication. This suggests that the loss or retention of duplicated genes may not occur randomly but may depend on the position and polarity of the copies (Lavrov et al. 2002; Xia et al. 2014).

Exceptions to the typical VertGO are piling up, also thanks to the increasing availability of complete mitogenomes, showing that the typical rearrangements can be generally classified as gene duplications (i.e., multiple and functional copies of the same gene occur in the same mitogenome), transpositions (i.e., genes occurring in a different place on the same strand), inversions (i.e., genes occurring on the opposite strand without transposition), or a combination of inversions and transpositions (fig. 1).

Various models have been proposed to explain GO rearrangements (see Bernt et al. 2013 for a review). The most frequently invoked models can be summarized in events of 1) tandem duplication and random loss, 2) intramitochondrial recombination, or 3) mitogenome dimerization and nonrandom loss. The transposition of a single gene is mostly explained by a tandem duplication (likely resulting from strand-slippage during replication) and random loss (tdrl) (fig. 1) (Moritz et al. 1987; Boore 2000; Bernt et al. 2013). Complicated patterns of rearrangement involving the simultaneous transposition of multiple genes, can be modeled by one or more TDRL events (usually indicated as an event and with a capital-letter acronym to distinguish it from a single gene tdrl, see also fig. 1, Bernt et al. 2007, 2008; Bernt and Middendorf 2011). In this case, a tandem duplication of a continuous segment of genes occurs and, at the beginning, the original segment and its copy are arranged consecutively (fig. 1). This duplication is usually followed by the loss of one copy of each redundant gene (Bernt and Middendorf 2011) therefore leading to a simultaneous change of position of multiple genes (fig. 1). More complex and particular is the case of multiple transpositions and CoRe duplications as observed in the flatfish *Samariscus latus* (Shi et al. 2014). The authors hypothesized that the *S. latus* GO originated from an initial duplication of the CoRe followed by a double replication of the whole mitogenome initiated from one of the two CoRes and random loss of redundant genes. The process results in two clusters of genes separated by the two Control Regions (Shi et al. 2014). The gene inversion can be modeled through an intramitochondrial recombination, an illegitimate recombination (Dowton and Campbell 2001; Lavrov et al. 2002; Bernt et al. 2013), or a head-to-tail dimerization of linearized monomeric mitogenomes that in some cases leads to polarity-driven nonrandom loss of duplicated genes (DMNL, e.g., in four flatfishes, Shi et al. 2013). The inverse transposition is explained by the combination of transposition and inversion or, more rarely, by recombination alone (fig. 1) (e.g., Basso et al. 2017).

These cases show that several alternative molecular mechanisms might contribute to the same rearrangement event (Bernt et al. 2013) and may occur in combination. Therefore, the lack of direct experimental evidence of each intermediate step makes it difficult to identify the main mechanism of rearrangement (Xia et al. 2016). Despite the study of mitogenomes is a very active field of research (e.g., Bernt et al. 2013, 2014; Cameron 2014; Satoh et al. 2016; Basso et al. 2017; Hartmann et al. 2018; Luo et al. 2019; Zhang et al. 2020), uncertainty and lack of precision of models still pose a challenge in explaining the evolution of GOs.

Indirect evidence for an intermediate step in a genomic rearrangement may come from the analysis of the position and content of ISPs (Basso et al. 2017). Two types of mitochondrial ISPs are known (e.g., Basso et al. 2017). The standard ISPs (STD-ISP) are generated by a strand slippage during the replication of the mitogenome (e.g., Salvato et al. 2008), and are the most frequent type of intergenic spacers in the Bilaterian mitogenomes. A second type of mitochondrial spacers is usually the result of a genomic rearrangement (GR-ISP). GR-ISPs are relicts of duplicated genes that have undergone a progressive loss of function and can sometimes be identified when the degenaration of the pseudogene is not complete. The occurrence of GR-ISPs in the positions hypothesized to be involved in a mitochondrial rearrangment is regarded as a first evidence supporting a reconstructed evolutionary pathway (San Mauro et al. 2006; Basso et al. 2017; Hartmann et al. 2018). A robust evidence of involvement in a genomic rearrangement usually comes from GR-ISPs containing unambiguously recognizable relicts of genes that lost function via degeneration (San Mauro et al. 2006; Jühling et al. 2012; Basso et al. 2017; Hartmann et al. 2018). The thorough analysis of GR-ISPs has been applied to a limited number of cases (e.g., Mueller and Boore 2005; San Mauro et al. 2006; Jühling et al. 2012; Basso et al. 2017), none of which were fish, to our best knowledge. One main limitation of the analysis of intergentic spacers owes to the fact that the pseudogenes contained in the GR-ISPs tend to disappear rapidly, due to strong selective pressure towards the maintenance of a compact mitogenome and a constant gene content (Wolstenholme 1992; San Mauro et al. 2006).

Understanding what factors lead to new GOs and if there is an adaptive nature of the rearrangements are additional challenges. The role of selection, mutation, and species biology from one side and the consequences of GO rearrangements in terms of phylogenetic output and mitochondrial function remain largely unexplored research venues.

It is generally expected that mitogenomic organization should evolve neutrally (Brown 1985) or under a condition of relaxed selective pressure (Xia et al. 2014). Nonadaptive forces, such as genetic drift or bottleneck, may drive the fixation and dispersal of mitogenomic reorganizations, whereas the demographics and life history of a species may explain the speed of fixation of largescale genomic modifications (Boussau et al.2011; Xia et al. 2014, 2016). However, this expectation is at odds with the presence of purifying selection which strongly preserves mitochondrial gene function in several species with gene order rearrangements (e.g., frogs, Xia et al. 2014, 2016). Advanced computational analysis of signatures of selective pressure at single and multigene scale in a phylogenetic framework can provide indications on the different contribution of neutral and adaptive forces on generation and retention of GO rearrangements (e.g., Smith et al. 2015; Wertheim et al. 2015).

From a phylogenetic point of view, the large number of potential gene order combinations means that the probability of the same GO occurring in separate lineages is extremely low and thus it is expected that the different GO may represent a class of genomic markers capable to define unambiguously monophyletic groups at different taxonomic rank (Boore et al. 1998; Boore 2006). Several mitochondrial GOs have proved to play this role in defining clades at both low and high taxonomic rank (e.g., Boore et al. 1995, 1998; Basso et al. 2017). In some cases, the rearranged gene orders characterize unambiguously vertebrate subclades like the Marsupialia and Crocodylidae (Pääbo et al. 1991; Kumazawa and Nishida 1995). However, since not all GO combinations of genes are equally probable, the differential susceptibility of some genes (tRNAs) and/or portions of the mitogenome to changes (e.g., Babbucci et al. 2014) increases the chances of homoplastic rearrangements (i.e., the presence of the same GO in unrelated groups), due to convergent or parallel evolution. Therefore, in some groups, like frogs, rearrangements are not diagnostic of a clade (Xia et al. 2014, for some other examples: Inoue et al. 2003). This limits the possibility of GO rearrangements to act as clade-defining genomic signatures (e.g., Babbucci et al. 2014) and suggests that sampling for mitogenomic studies should always aim at the largest taxon coverage possible.

Useful models to begin investigating challenging topics related to mechanisms of rearrangement, impact of GO changes on phylogenetic output, and role of selection on generation and retention of new GO come from fish radiations. Rapid evolutionary radiations are frequently credited to be the driving force of structural genomic rearrangements or duplications (Turner 2007; Coppe et al. 2013; Poulsen et al. 2013).

The Southern Ocean is dominated by the in situ radiation of highly specialized and geographically restricted Antarctic fish of the suborder Notothenioidei, a prime example of a marine species flock. This suborder includes four families, the Bovichtidae, Pseudaphritidae, Eleginopsidae, and the Nototheniidae (Duhamel et al. 2014). The first three families were the first to diverge and comprise only non-Antarctic species. The Nototheniidae encompass all Antarctic and secondary non-Antarctic species and are sometimes referred as Cryonotothenioidea (sensu Dornburg et al. 2017 and Near et al. 2018). Notothenioids have exceptionally high rates of species formation compared with most temperate and tropical ray-finned fish taxa (Rabosky et al. 2018). Although the Antarctic notothenioids have radiated quickly over ∼20–25 Ma (Dornburg et al. 2017), they have developed an astonishingly large variety of physiological, ecological, behavioral, morphological, and life-history characteristics (Eastman 2005 and Matschiner et al. 2015 for a review). For all these characteristics, notothenioids are a developing model system to investigate evolutionary biology and ecology questions.

Notothenioids are mainly renowned for their peculiar physiological adaptations to cold. These include the ability to synthesize antifreeze glycoproteins (AFGP) and antifreeze-potentiating proteins (AFPP) (for a review see Duman 2015). The ecological hallmark of the notothenioid’s radiation is an evolutionary adaptation of morphology and physiology for life in the water column (Eastman 2005), sometimes referred to as pelagization, and is enabled by a combination of lack of swim bladder, lipid deposition, and reduced skeletal mineralization that allows reductions in density and alterations in buoyancy (Eastman 2005; Chen et al. 2019). Pelagization is most strongly evident in the species of the subfamilies Pleuragramminae and Dissostichinae, all of which show adaptations towards neutral buoyancy (Eastman and DeVries 1989; Eastman 1993; Near et al. 2003, 2007). Other notothenioids show reductions in density that appear to facilitate bentho-pelagic strategies, in which fish move into the water column to feed. Strongly negative buoyancy remains a characteristic primarily of species associated with benthic habitats (Eastman 1985, 2017; Eastman and Sidell 2002).

The ecological diversification of Antarctic notothenioids and the selective pressure of the cold have also elicited remarkable changes in the structure and gene content of cryonotothenioid nuclear genomes. These modifications range from extensive cold-specific gene duplications and functional diversification (e.g., the AFGP family and duplicated Zona Pellucida [ZP] genes to aid in cellular freezing resistance), to the expansion of transposable elements and high frequency of nuclear chromosomal rearrangements (Pisano and Ozouf-Costaz 2000; Chen et al. 2008, 2019; Coppe et al. 2013; Cao et al. 2016; Bargelloni et al. 2019; Auvinet et al. 2020).

Given these premises, it may be expected that the rearrangements of the mitochondrial gene order documented in notothenioids may be the result of the selective pressure of the environmental conditions. The mitochondrial genomes of Antarctic notothenioids have undergone extensive gene order rearrangements in the form of transposition and duplication of the genes and the CoRe encoded in the genomic region delimited by *nad5* and *trnF* (hereafter *nad5*-*trnF* region) (Zhuang and Cheng 2010). Among the 11 mitogenomes (2 partial and 9 complete, see supplementary table S2, Supplementary Material online) available at the time of this study, six different GO were already known for Notothenioidei. These preliminary data showed that all early-diverged species carried the VertGO arrangement, whereas extensive gene order rearrangements characterized the Antarctic family (Nototheniidae, Zhuang and Cheng 2010) suggesting that all rearrangements and the radiation in the newly formed polar environment occurred coincidentally. The mitogenomes of *Bovichtus argentinus* and *Eleginops maclovinus* exhibit the VertGO (Satoh et al. 2016; Lee-Estevez et al. 2019) and also in *Pseudaphritis urvillii*, the sole species of the family Pseudaphritidae, the *nad5*-*trnF* region is arranged as in VertGO (the only structural information available for this species, Zhuang and Cheng 2010). In the cryonotothenioid, five mitogenomic arrangements different from VertGO characterize all mitogenomes analyzed to date (Zhuang and Cheng 2010; Lin et al. 2012; Lee et al. 2015a; Lee et al. 2015b; Oh et al. 2016; Song et al. 2016). All rearrangements occur in the *nad5*-*trnF* region (fig. 1).

Among the five documented GOs, three (hereafter named Noto1GO, Noto2GO, and Noto3GO) are shared among different cryonotothenioid species (fig. 3) (Zhuang and Cheng 2010; Lee et al. 2015a, 2015b; Oh et al. 2016; Song et al. 2016). A fourth GO (hereafter named ChamGO) was retrieved only in the mitogenome of *Champsocephalus gunnari* (subfamily Channichthyinae, Lin et al. 2012). The fifth GO (hereafter named DissoGO) is described in this study for the first time and resulted from the analysis of the mitogenomes of *Dissostichus eleginoides* and *Dissostichus mawsoni* (unpublished, but available in GenBank).

Although the mitogenomes of few cryonotothenioids have been available for years, fundamental questions remain unaddressed about 1) the extent of the GO variability within the whole Antarctic clade, 2) the mechanisms and 3) the evolutionary pathways that led to the mitochondrial structural diversity, 4) the different contribute of drift and selection in shaping the GO diversity, and 5) the consequences of gene order rearrangements on phylogenetic output and mitochondrial function in the Notothenioidei. To address these questions about the Antarctic notothenioid mitogenomic evolution, we investigated the origins and steps resulting in the observed gene rearrangements by 1) expanding the sampling to 15 complete and two nearly complete new mitogenomes of notothenioids, 2) determining the structure of the rearranged regions for each GO, 3) analyzing the position and content of all available GR-ISPs, 4) assessing the type of selection acting on mitochondrial genes, the compositional bias, and the homogeneity of the substitution pattern, 5) tracing the evolution of each GO on an inferred species phylogeny, and 6) investigating the impact of the GO rearrangements on the phylogenetic output. Our results posit new avenues of research to test our preliminary speculations about the adaptive or nonadaptive nature of the extensive rearrangements and the consequences on mitochondrial function.

## Materials and Methods

### Biological Sample Collection and Handling

The species sequenced in this study were collected during different Antarctic campaigns (details are provided in supplementary table S1, Supplementary Material online). Most of the samples were collected by bottom trawl and tissues are stored in ethanol 95% at +4°C at the Biology Department of the University of Padova (Italy), at the Muséum national d’Histoire naturelle (MNHN, Paris), or at the Alfred Wegener Institute Helmholtz Centre for Polar and Marine Research (Bremerhaven, Germany). For further details on sampling, see supplementary Materials and Methods, Supplementary Material online.

### Genome Sequencing and Annotation

Fifteen complete and two partial mitogenomes were sequenced for this study. The complete mtDNAs were obtained by the Illumina technology or long PCR (Hinsinger et al. 2015) sequencing that allowed to ascertain gene order and inversions. The two partial genomes were generated through standard PCR with universal primers followed by Sanger sequencing (for further details, see supplementary Materials and Methods, Supplementary Material online). The Sanger chromatograms were assembled using the DNASTAR package (Madison, WI). The Illumina reads were assembled de novo using the CLC Genomics Workbench 8.5 (Qiagen, Hilden, Germany) and the program MITObim (Hahn et al. 2013) as described in Basso et al. (2017). Annotation of the new mitochondrial genomes was performed according to the strategy described in Montelli et al. (2016), whereas for Control Regions, we followed Zhuang and Cheng (2010). To ensure a homogenous annotation for the full set of studied genomes, sequences available in GenBank were reannotated following the same approach described above. The two strands of the mitogenome are indicated as H-strand and L-strand following the standard nomenclature for Vertebrata (fig. 1) (e.g., Satoh et al. 2016).

### Taxa Selection

The taxa analyzed in this work are listed in supplementary table S2, Supplementary Material online. We studied 28 species covering all major notothenioid clades. We excluded some sequences available in GenBank because of poor quality (further details in supplementary table S2 and Materials and Methods, Supplementary Material online). One special note concerns the mitogenomes that have become available after the completion of our analyses and before the publication of this study. The mitochondrial genomes of *Chionobathyscus dewitti* (Andriyono et al. 2019; Genbank record published on March 9, 2020), *Pogonophryne albipinna* (Tabassum et al. 2020; Genbank record published on February 3, 2020)*, Notothenia rossii* (MT192936, only Genbank record, published on May 10, 2020), and *Trematomus loennbergii* (MT447073, only Genbank record, published on June 9, 2020) did not become available in GenBank in time to be included in our most advanced analyses. However, we annotated the new mitogenomes and we will consider their GO and published phylogenetic relationship with the species considered in our analyses when relevant for the aims of the study.

### The Genomic Compositional Bias and the Impact on the Phylogenetic Output

The two strands of the mitogenome differ in their nucleotide composition due to an asymmetric mutation process that favors transitions over transversions (Hassanin et al. 2005). The result of this process is that the H-strand in vertebrate mitogenomes is usually GT-rich, whereas the L-strand is typically AC-rich. This is probably due to the hydrolytic deamination of A (A →G) and C (C → T), of the single H-strand during replication and, to a less extend, during transcription (Bernt et al. 2013).

The strand compositional bias of mitochondrial genomes can be impacted by gene order rearrangements. In the most extreme case, when an inversion of the Control Region occurs, the compositional bias can be completely inverted (Fonseca et al. 2008, 2014). In this case, it is expected that both the AT-skew and GC-skew (GC-skew = (G−C)/(G + C); AT-skew = (A−T)/(A + T)) of mitochondrial protein-coding genes (PCGs) are affected, particularly the third positions. This results in AT-skews and GC-skews of different absolute value and in extreme cases also of opposite sign compared with unarranged mitogenomes. If this effect is not accounted for, it could impact the phylogenetic output (e.g., cases of long-branch attraction or misplacement of taxa in the phylogeny) when analyses are performed including genes that exhibit the strongest bias,that is, a complete reversal of the strand compositional bias (Hassanin et al. 2005). Since in this study we documented an instance of inversion of the Control Region (in Trematominae, see Results and Discussion), we verified the impact of gene order rearrangements on compositional bias (Perna and Kocher 1995) by computing the sign and value of AT-skews and GC-skews for each mitochondrial gene, for the ribosomal genes, and for the entire mitogenomes (supplementary figs.S17–S23, Supplementary Material online). Subsequently, we verified with a Student’s*t*-test whether the absolute values of the AT-skews and the GC-skews in taxa affected by gene inversion were significantly different from all other notothenioids.

The occurrence of a global reversal of the strand compositional bias that affects the strand symmetry and the composition of third positions in some mitogenomes was assessed following the strategy described in Hassanin et al. (2005). Initially, two data sets containing synonymous third positions of the 2-fold (NNR2+NNY2) and 4-fold (NNN4) degenerate codons from all PCGs were generated with the CAIcal server (http://genomes.urv.es/CAIcal/, last accessed February 12, 2021) (Puigbo et al. 2008). This step allowed to generate two data sets large enough to perform a statistical analysis (Hassanin et al. 2005). The synonymous third positions of genes translocated to the opposite strand during the mitogenomic rearrangements were excluded from NNN4 and NNR2+NNY2 data sets to avoid effects due to strand change (Hassanin et al. 2005). Accordingly, *nad1* was excluded from our analysis because it is located in a different strand in the mitogenomes of Trematominae (see Results and Discussion for details). The AT4-, GC4-skews and AT2-, GC2-skews were then computed for NNN4 and NNR2+NNY2 data sets and the hypothesis of strand symmetry (i.e., not significant differences in compositional biases) was tested by applying the statistical equations developed by Hassanin et al. (2005, calculated with Excel ver. 2016, Microsoft). We concluded that a global reversal of the strand compositional occurred in a specific mitogenome if simultaneously: 1) the AT4-/GC4-skews and AT2-/GC2-skews carry a sign opposite to that expected for the analyzed set in case of no inversion, and 2) the hypothesis of strand symmetry is rejected at *P* < 0.05 for both A versus T and G versus C compositions (Hassanin et al. 2005). Finally, to verify if the change in compositional bias had any visible effect on the phylogenetic output based on the dataset of species used in this study, we repeated the phylogenetic analyses described below in this section (Materials and Methods) only using the mitochondrial genes (*28 T.Mito dataset*) with the best partition scheme approach and the heterotachy evolutionary model (Chernomor et al. 2016; Crotty et al. 2019).

### Type of Selection Acting on Mitochondrial Protein-Coding Genes

**Fig. 2.**
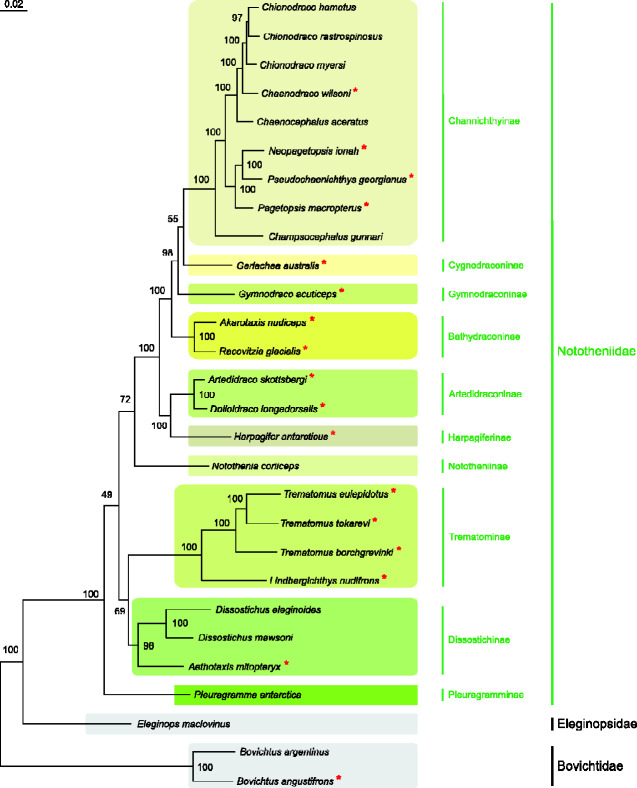
Maximum Likelihood tree (−ln = 145,314.7783) obtained from the analysis performed on the *28 T.Mito* + *28 T.Nucl* multiple alignments by applying the GTR+FO×H4 heterotachous model to the two s. Ultrafast bootstrap values are provided for each node. The scale bar represents 0.2 substitutions/site. Species whose genomes were sequenced de novo in this study are indicated by an asterisk.

To assess what type of selection (purifying, neutral or positive) shaped the evolution of the mitochondrial protein-coding genes involved in the GO rearrangements and the genes with a stable position, we applied two approaches. First, we computed the pairwise comparisons of Ka/Ks ratios (Ka, the number of nonsynonymous substitutions and Ks the number of synonymous substitutions, Nei and Gojobori 1986) with the program DnaSP 6.12.03 (Rozas et al. 2017). The distribution of the pairwise Ka/Ks ratios was represented with a box plot with Excel ver. 2016 (Microsoft). Second, we applied the phylogeny-based strategy implemented in the programs aBSREL (adaptiveBranch-Site RandomEffects Likelihood) and RELAX (available at the DataMonkey 2.0 web site, https://www.datamonkey.org/, last accessed February 12, 2021, Smith et al. 2015; Wertheim et al. 2015; Weaver et al. 2018) to test for selection across branches of a reference topology based on an alignment of orthologous genes. The program aBSREL tests for the occurrence of events of episodic diversifying selection along selected single branches of a topology (Smith et al. 2015). The program RELAX tests whether the strength of natural selection has been relaxed or intensified along a specified set of branches (Wertheim et al. 2015). By tracking their evolution at gene level, the two approaches were used to test the occurrence of taxon-specific changes in the 13 PCGs. The target species for the test were identified by selecting branches along which changes in gene order occurred or that lead to cryonotothenioid clades containing two or more species. As reference topologies, we considered the two trees obtained in this study (based on the heterotachy approach and on the best partition scheme, see section “A Multigene Phylogeny for the Suborder Notothenioidei”) and compared results. The selected branches, and corresponding taxa tested in separate runs were (see fig. 2 and supplementary fig. 36, Supplementary Material online): 1) the family Nototheniidae, 2) *Pleuragramma antarctica*, 3) the subfamily Dissostichinae, 4) *Aethotaxis mitopteryx*, 5) the *Dissostichus* genus, 6) the subfamily Trematominae, 7) *Notothenia coriiceps*, 8) *Harpagifer antarcticus*, 9) the subfamily Artedidraconinae, 10) the subfamily Bathydraconinae, 11) *Gymnodraco acuticeps*, 12) the subfamily Channichthyinae, and 13) *Champsocephalus gunnari*. A detailed description of the list of test and reference branches selected for the analyses with aBSREL and RELAX is provided in supplementary Materials and Methods, section “Type of Selection Acting on Mitochondrial Protein-Coding Genes: A Phylogeny-Based Strategy,” Supplementary Material online.

### Gene Selection and DataSet Generation for the Phylogenetic Analysis

For all the 28 species, we created single gene orthologous alignments encompassing 15 mitochondrial genes (13 PCGs and 2 ribosomal genes) and 8 nuclear markers (supplementary table S3, Supplementary Material online). Single markers were aligned with the program MAFFT (Katoh et al. 2002, 2005).

Single gene alignments were concatenated in two multimarker data sets. The first, named *28 T.Mito* contained 15 mitochondrial genes (13 protein-coding genes, plus *rrnS* and *rrnL*) and was 13,791 positions long. The second, named *28 T.Nucl* included the eight nuclear markers (supplementary table S3, Supplementary Material online) and was 6,619 positions long.

Further details on the strategy and rationale followed to select the genes for the phylogenetic analysis are provided in supplementary Materials and Methods, Supplementary Material online.

### Heterogeneity of the Substitution Pattern, Detection of Phylogenetic Signal, and Phylogenetic Analysis

To preliminary check the overall level of heterogeneity of the substitution pattern, we analyzed the multiple alignment of each gene with the software AliGROOVE (Kück et al. 2014) (supplementary figs. S25–S35, Supplementary Material online).

To test for the presence of phylogenetic signal in the single marker alignments, we applied the likelihood mapping approach (Strimmer and von Haeseler 1997) implemented in the program IQ-TREE 1.6.9 (Nguyen et al. 2015). The percentage of Fully Resolved Quartets (%FRQ) was considered as an estimation of the phylogenetic signal (Strimmer and von Haeseler 1997) (supplementary figs. S25–S35, Supplementary Material online).

Maximum likelihood phylogenetic analyses were performed on *28 T.Mito* + *28 T.Nucl* sets using the program IQ-TREE 1.6.9 by applying different strategies. Phylogenetic reconstructions were done by applying the best partition scheme (Chernomor et al. 2016), as well as the heterotachy model, that allow to accommodate the level of substitution heterogeneity detected in the molecular markers (Crotty et al. 2019). Statistical support to the nodes of the best tree were computed by applying the ultrafast bootstrap test (10,000 replicates) (Hoang et al. 2018). Finally, a maximum likelihood phylogenetic analysis was performed on the multiple alignment containing the Control Regions to study the evolution of these genomic segments, present in different number in the cryonotothenioid species (further details in supplementary Materials and Methods, Supplementary Material online).

### Gene Order Evolution

The reconstruction of GO on internal nodes of the reference phylogeny and the inference of the transformational genomic pathways connecting two different GOs were obtained by manually applying a maximum parsimony approach. The available models on mitochondrial rearrangements were used as guidelines (Moritz et al. 1987; Boore 2000; Dowton and Campbell 2001; Basso et al. 2017). The analysis of intergenic spacers associated to the mitochondrial genomic rearrangements guided and strongly corroborated our reconstruction of the gene order evolutionary pathways (see Supplementary Material online).

### Dating Key Nodes in the Reference Phylogeny Tree

We dated some nodes of the reference tree linked to the appearance of the mitochondrial genomic rearrangements by using the chronogram provided by Dornburg et al. (2017). Geological Epoch/Age naming follows Ogg et al. (2016).

## Results and Discussion

### Mitochondrial Genomic Diversity in the Notothenioidei

Our analysis encompassed 28 notothenioid mitogenomes including 17 newly sequenced for this study. Among the newly sequenced mitogenomes, we found instances of all six GOs known to date (VertGO, Noto1GO, Noto2GO, Noto3GO, DissoGO, and ChamGO) (supplementary figs. S3–S5, Supplementary Material online). *Bovichtus angustifrons* is characterized by the VertGO thus confirming what already known for *B. argentinus* (Bovichtidae) and *E. maclovinus* (Eleginopsidae) members of the families that branch off at the base of notothenioid radiation (Dornburg et al. 2017; Near et al. 2018). *Champsocephalus gunnari* is still the only species, among the Channichthyinae sequenced to date, to have this specific mitochondrial GO. All other Channichthyinae share the Noto3GO, included the recently available mitogenome of *C. dewitti* (Andriyono et al. 2019) although six other Channichthyinae mitochondrial genomes have yet to be sequenced.

Three new GOs (RacoGO, GymnGO, and TremaGO) are described in this study for the first time. RacoGO characterizes *Racovitzia glacialis* (Bathydraconinae) and GymnGO was retrieved only for *Gymnodraco acuticpes* (Gymnodraconinae). Both these new GOs carry a rearrangement in the *nad5*-*trnF* region, as typical of all GO modifications of cryonotothenioids. The *R. glacialis* RacoGO and *G. acuticeps* GymnGO are unique types of mitochondrial arrangements, but they might be shared by close relatives of these species (*Vomeridens infuscipinnis* and *Prionodraco evansi* for *R. glacialis*, and *Acanthodraco dewitti* for *G. acuticeps*) (Dettai et al. 2012). TremaGO was found in all *Trematomus* species (Trematominae) analyzed in this study (included the mitogenome of *T. loennbergii*), and in *Lindbergichthys nudifrons* (Trematominae). The TremaGO is the most complex mitochondrial rearrangement observed in the Nototheniidae clade because it is one of very few examples of an inversion of a large genomic segment (at least 5,300 bp) in fish mtDNA, an event that is generally regarded as very rare (Satoh et al. 2016). Indeed, there are only two records of gene inversion in fish: in the tongue sole *Cynoglossus semilaevis* and black cow-tongue *Paraplagusia japonica* (Pleuronectiformes, Cynoglossidae) a single *trnQ* is inverted and located on the opposite strand (Kong et al. 2009; Gong et al. 2013). As explained more in detail in the next sections, some of these peculiar gene orders are molecular signatures uniquely supporting some of the clades, although others might have been developed several times independently.

### A Multigene Phylogeny for the Suborder Notothenioidei

To trace the evolution of each GO and to reconstruct the time at which each rearrangement arose, we generated a reference phylogeny of the suborder Notothenioidei using 15 mitochondrial and 8 nuclear genes. To take into account the potential impact of the transition from temperate to cold waters in the evolution of GO, we covered 3 notothenioid families including both Antarctic (25 species) and non-Antarctic (3 species) taxa (supplementary tables S1–S3, Materials and Methods, Results and Discussion, Supplementary Material online). We obtained two phylogenetic trees, one based on the heterotachy approach and one applying a best partition scheme (Chernomor et al. 2016; Crotty et al. 2019). In both trees, most nodes received a high statistical support (>95%) (fig. 2 and supplementary fig. S1, Supplementary Material online). The two trees differ for the position of *P. antarctica* and none of the placements of *P. antarctica* in the two topologies is well supported by ultrafast bootstrap values (fig. 2 and supplementary fig. S1, Supplementary Material online). In the topology obtained by applying the best partition scheme, *P. antarctica* is sister taxon of the clade formed by Dissostichinae + Trematominae (supplementary fig. S1, Supplementary Material online). In the topology obtained by applying the heterotachous evolutionary model, *P. antarctica* is the sister taxon of the other Nototheniidae (fig. 2). When only mitochondrial genes are considered, *P. antarctica* is sister taxon of the clade formed by Dissostichinae + Trematominae with both models (see the section “The Inversion of the Control Region in Trematominae: Genomic Compositional Bias and Impact on the Phylogenetic Output”). Thus, the model used to account for the heterogeneity of the substitution process (heterotachy or best partition scheme) has an impact on the arrangement of the basal nodes of the cryonotothenioid clade, when also nuclear genes are analyzed and leads to alternative placements of *P*. *antarctica*. In agreement with our finding, the branching pattern for the most basal nodes of the Nototheniidae clade is not yet well established (e.g., Near et al. 2012, 2018; Dornburg et al. 2017). We used the tree obtained applying the heterotachous evolutionary model as a reference topology since we demonstrated that some level of heterogeneity in the substitution process exists in our datasets (supplementary Results and Discussion, Materials and Methods, and figs. S22–S32, Supplementary Material online). However, we mapped the gene order evolution also on the alternative topology obtained with the best partition scheme (supplementary figs. S1 and S2, Supplementary Material online). The application of two models of substitution process and the alternative placements of *P. antarctica* had no impact on the reconstruction of GO evolution. The pathways of GO evolution, described in detail in the next section, are therefore valid for both tree topologies obtained in this study.

### Evolutionary Pathways of Gene Order Rearrangement

**Fig. 3.**
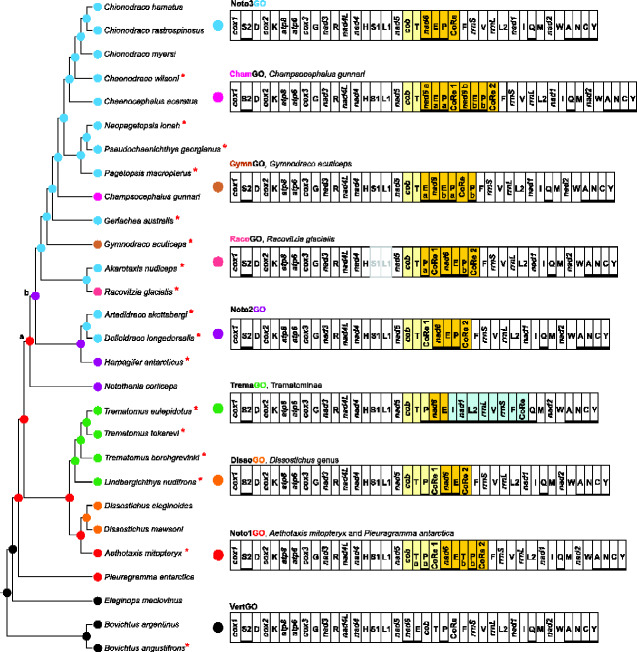
Mitochondrial gene order evolution mapped on the phylogeny of Notothenioidei.The evolution of the mitochondrial gene order is mapped on the reference phylogenetic tree obtained via Maximum Likelihood approach. Every gene order is indicated as GO and is linearized starting from *cox1*. The genomic and genetic nomenclature are the same as in figure 1. Genes transposed/duplicated with respect to the VertGO have a yellow (gene belonging to the 5′ duplicated block) and orange (gene belonging to the 3′ duplicated block) background, whereas genes translocated and moved on the L-strand have a light blue background. Genomes sequenced de novo in this study are indicated by an asterisk. Missing regions in incompletely sequenced genomes (*Racovitizia glacialis*) are in light gray.

**Fig. 4.**
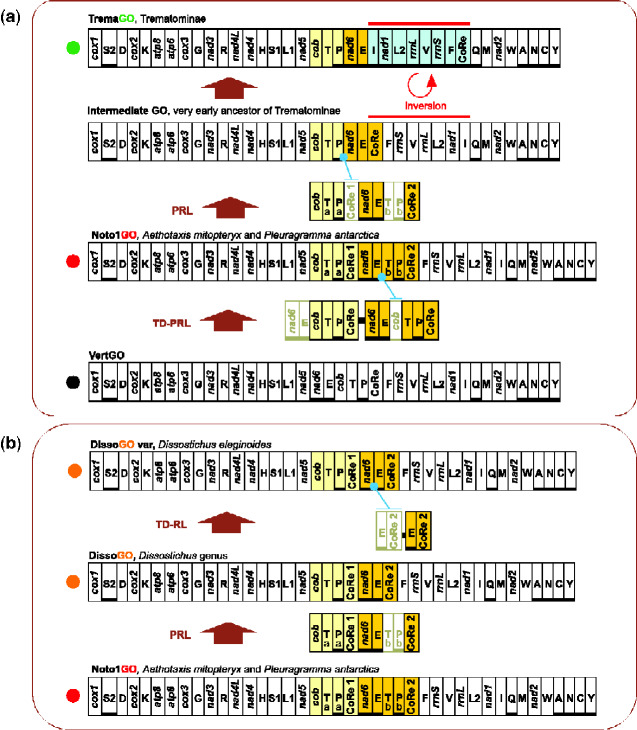
Pathway 1 and 2: the evolution of mitochondrial gene orders Noto1GO, DissoGO, and TremaGO in Nototheniidae.(*a*)Pathway 1:Evolution of the Noto1GO, and the TremaGO in Trematominae.(*b*) Pathway 2:Evolution of the DissoGO in *Dissostichus* genus.The genomic and genetic nomenclature, as well as the color scheme, are the same as in figures 1 and 3. TD-PRL, tandem duplication and partial random loss; TD, tandem duplication; PRL, partial random loss. Copies of the genes lost during the genomic rearrangement are framed in light green. A blue dot points to an intergenic spacer generated during the rearrangement process, which includes remnant/s of the lost duplicated gene/s.

**Fig. 5.**
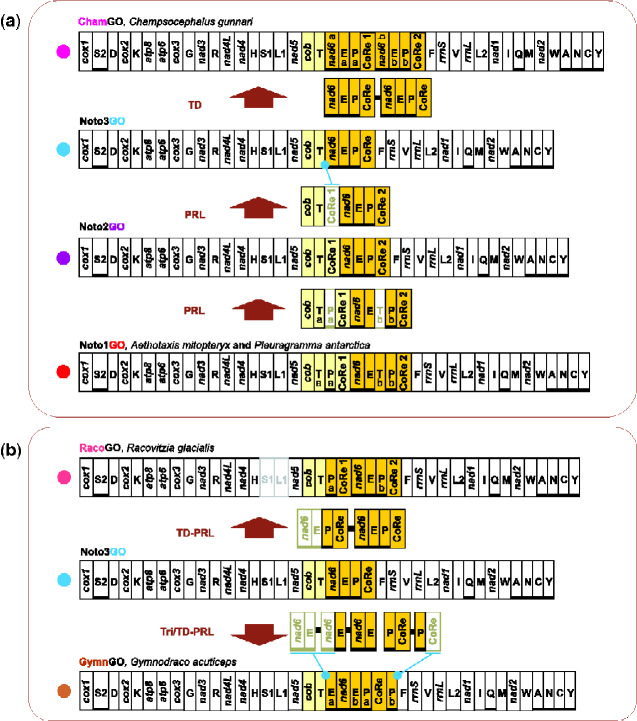
Pathway 3 and 4: the evolution of five mitochondrial gene orders in Nototheniidae. (*a*)Pathway 3: Evolution of Noto2GO, Noto3GO, and *Champsocephalus gunnari* ChamGO. (*b*)Pathway 4: Evolution of *Gymnodraco acuticeps* GymnGO, and *Racovitzia glacialis* RacoGO. The genomic and genetic nomenclature, as well as the color scheme, are the same as in figures 1 and 3. Tri-PRL, tandem triplication and partial random loss; TD-PRL, tandem duplication and partial random loss; TD, tandem duplication; PRL, partial random loss. Copies of the genes lost during the genomic rearrangement are framed in light green. A blue dot points to an intergenic spacer generated during the rearrangement process, which includes remnant/s of the lost duplicated gene/s.

According to our reconstruction and the previous research of Zhuang and Cheng (2010), *P. antarctica* and *A. mitopteryx* share the arrangement Noto1GO (figs. 3–5). Even taking into account the uncertainty on the position of the basal nodes of the family Nototheniidae (fig. 2 and supplementary fig. S1, Supplementary Material online), the Noto1GO could be considered as the ancestral condition for all nototheniids and arose at the beginning of the radiation (Zhuang and Cheng 2010 and this study) (fig. 3 and supplementary fig. S2, Supplementary Material online), around 22 Ma in the early Miocene (Aquitanian, following Dornburg et al. 2017). In the most parsimonious reconstruction of the mitochondrial genomic rearrangements, the Noto1GO derived from the VertGO via an event of tandem duplication partial random loss (TDPRL) (fig. 4*a*) (Zhuang and Cheng 2010). The hypothesis is that the segment spanning from *nad6* to the CoRe was initially duplicated and successively the partial random loss of some of the redundant elements resulted in the presence of two copies of *trnT*, *trnP* and CoRe and the transpositions of *cob* and *nad6 *+* trnE* to a position between one CoRe and a duplicated *trnT* (process described in Zhuang and Cheng 2010). All other gene orders documented in the nototheniids derived from the Noto1GO through further rearrangements and, as our analyses demonstrate, followed four main transformational pathways (figs. 3–5). The order of the pathways, as they are described below, is not meant to reflect the succession of their appearance during the evolution of notothenioids.

The first transformational pathway reconstructs the evolution of an exceptional and extremely rare rearrangement among the documented GOs in vertebrate mtDNAs and leads from the Noto1GO to the TremaGO via an intermediate, yet unsampled, GO (fig. 4*a*). The loss of CoRe1 and T_b_-P_b_ that characterized the transition from Noto1GO to an intermediate GO occurred before the diversification of Trematominae (∼12 Ma, Miocene, late Serravalian, dating according to Dornburg et al. 2017). The condition of the intermediate GO was followed by the inversion of a long mitochondrial segment including seven genes (fig. 4*a*) from CoRe to *trnI* and resulted in the final TremaGO. In particular, these rearrangements led to the inversion and relocation of the Control Region on the opposite strand.

In the second transformational pathway, the DissoGO arrangement (figs. 3 and 4*b*) evolved ∼8 Ma (Miocene, late Tortonian, Dornburg et al. 2017) from the Noto1GO through the loss of the *trnT*-*trnP* pair (T_b_-P_b_) located downstream of *trnE* (fig. 4*b*). Furthermore, a successive duplication of the segment *trnE*-CoRe2 and the subsequent loss of the upstream copies of both genomic elements occurred only in *D.eleginoides*. This can be deduced by the presence of remnants of the duplicated and lost elements *trnE* and CoRe2 in a spacer sequence of *D. eleginoides* located between *nad6* and CoRe2 (fig. 4*b* and supplementary Results and Discussion andfigs. S3, S10, and S11, Supplementary Material online).

The third transformational pathway leads from the Noto1GO to the Noto3GO via the intermediate arrangement Noto2GO and is characterized by multiple losses of mitochondrial genomic elements (figs. 3 and figs. 5*a*). The arrangement Noto2GO (∼18 Ma, Miocene, Burdigallian, Dornburg et al. 2017), found in both *N.coriiceps* and *Harpagifer antarcticus*, evolved from the Noto1GO through the loss of *trnP* (P_a_) and the downstream copy of *trnT* (T_b_). Our results suggest that the transition from Noto1GO to Noto2GO occurred independently in *N*. *coriiceps* and in the common ancestor of the clade including Harpagiferinae, Artedidraconinae, Bathydraconinae, Gymnodraconinae, Cygnodraconinae and Channichthyinae (node b in fig. 3, see also fig. 5*a*). In *N. coriiceps*, Noto2GO appeared after the divergence from *N. rossii* (≤6.7 Ma, Miocene, Messinian; Dornburg et al. 2017). This can be inferred from the mitogenomic rearrangement of *N. rossii*, which carries a modification of Noto1GO, where the segment *trnT_a_*-*trnE* is duplicated. Furthermore, the *nad5*-*trnF* region of *Notothenia angustata* is arranged as in Noto1GO (Zhuang and Cheng 2010). These observations strongly support the view that Noto1GO represents the ancestral condition for the genus *Notothenia* (node a in fig. 3). Therefore, the second emergence of Noto2GO could be located at node b (fig. 3; ∼13.3 Ma, Miocene, Serravallian; Dornburg et al. 2017).

In the subfamilies branching out from node b (fig. 3), the transition from Noto2GO to Noto3GO occurred twice: first in the lineage including Bathydraconinae, Gymnodraconinae, Cygnodraconinae, and Channichthyinae (∼12 Ma, Miocene, Serravallian) and second, more recently, in Artedidraconinae (∼3.22 Ma, Pliocene, Placenzian, Dornburg et al. 2017). The mitogenome of *Pogonophryne albipinna* (Tabassum et al. 2020) carries the Noto3GO in agreement with what found for otherArtedidraconinae in this study (figs. 2 and 3). The presence of clearly identifiable remnants of CoRe1 in the *trnT*-*nad6* intergenic spacer (e.g., in the species *Artedidraco skottsbergi*, *Pagetopsis macropterus*, *Akarotaxis nudiceps*) lends further support to the independent and parallel evolution of Noto3GO from Noto2GO (fig. 5*a* and supplementary figs. S12–S14, Supplementary Material online; for further details, see next section “Intergenic Spacers Linked to Genomic Rearrangements”).

The fourth transformational pathway accounts for the transition from Noto3GO to RacoGO, GymnGO, and ChamGO (fig. 5). A TDPRL event involving one genomic segment spanning *nad6*-CoRe in the Noto3GO generated the RacoGO observed in *R. glacialis* (≤5.47 Ma, boundary of Miocene/Pliocene, Dornburg et al. 2017) (fig. 5*b*). The rearrangement GymnGO, observed in *G. acuticeps*, derived from the Noto3GO (≤6.95 Ma, Miocene, late Messinian, Dornburg et al. 2017) through a complex pattern of triplication/duplication of genomic segments followed by partial random loss of redundant copies. As also supported by the analysis of the intergenic spacer in *G. acuticeps*, a parsimonious reconstruction of the mechanism that led to this rearrangement implies: 1) the triplication of the *nad6*-*trnE* segment followed by the loss of the upstream *nad6*-*trnE*-*nad6* copies, and 2) the tandem duplication of *trnP*-CoRe followed by the loss of the downstream copy of CoRe (supplementary figs. S4, S15, and S16, Supplementary Material online). The GymnGO retains two copies of *trnE* (E_a_ and E_b_) and *trnP* (P_a_ and P_b_). The complete duplication of the entire segment *nad6*-CoRe in the Noto3GO, and no successive loss, generated the ChamGO arrangement found in *C. gunnari* (≤1.77 Ma, Pleistocene, Calabrian, Dornburg et al. 2017) (fig. 5*a*).

Our reconstructions show multiple and complex events of rearrangement and posit a high structural plasticity of the cryonotothenioid mitogenomes. Several lines of evidence support the four evolutionary pathways reconstructed in this study following the most parsimonious approach. However, we cannot exclude that alternative molecular mechanisms could have contributed to the structural evolution of cryonotothenioid mitogenomes also considering that some intermediate steps are only hypothesized and have not yet been documented in the cryonotothenioid mitogenomes.

We are confident to exclude that the diversity of GOs found in this study is the result of incorporation of portions of nuclear mitochondrial DNA sequences (numts) in the cryonotothenioid mitogenomes generated by assembling sequences obtained from total DNA (supplementary Materials and Methods, Supplementary Material online). Frameshifts, stop-in-frame codons, and deviations from the vertebrate mitochondrial genetic code are typical clues of numts incorporation (Antunes and Ramos 2005) and none of these artefacts were found in the mitogenomes generated in this study. The sequence identity was high among mitochondrial orthologous genes/proteins and they always had very similar or identical length. Thus, we did not observe features that could be associated with the occurrence of numts in all mitogenomes considered in this study. In addition, the new mitogenomes obtained in this study were sequenced via an Illumina approach and the total DNA was extracted from muscle tissue, which is particularly rich in mitochondrial DNA relatively to nuclear DNA. Therefore, if present, numts should have been covered by a much lower number of reads than those of true mitochondrial origin. Instead, our consensus sequences were homogenously covered by a very high number of reads (supplementary Materials and Methods, Supplementary Material online).

### Intergenic Spacers Linked to Genomic Rearrangements

**Fig. 6.**
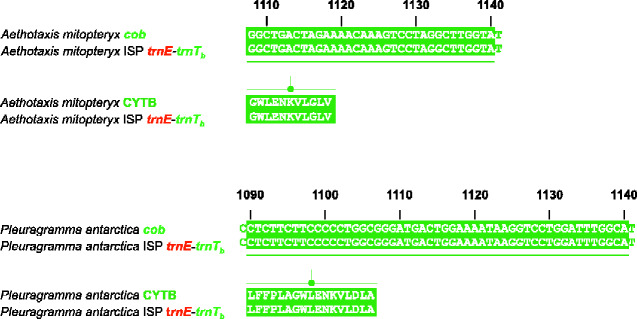
Pairwise-alignments of conserved DNA segments present in the GRT-ISP *trnE*-*trnT_b_* and *cob* genes of *Aethotaxis mitopteryx* and*Pleuragrammaantarctica*. The portions of the sequences encoding identical polypeptides are depicted with a green background. Gene/protein nomenclature as in figure 3.

In the 28 mitochondrial genomes considered in this study, we identified and thoroughly investigated 20 GR-ISPs (figs. 3–5 and supplementary Results and Discussion and figs. S3–S5 and S6–S16, Supplementary Material online). Possibly owing to the relatively recent age of the cryonotothenioid clade, many GR-ISPs contained reliable remnants of the genes originally involved in the mitogenomic rearrangement. As described for *D. eleginoides* and *G. acuticeps*, the analysis of GR-ISPs contributed to a more robust reconstruction of the mechanisms and the pathways hypothesized as responsible for the appearance of the GOs observed in Antarctic notothenioids (supplementary Results and Discussion and figs. S3–S5 and S6–S16, Supplementary Material online). For example, in *A. mitopteryx* and *P. antarctica*, the GR-ISPs between the *trnE* and *trnT_b_* (*trnE*-*trnT_b_*) contains a region identical to the 3′ end of the gene *cob* (34 and 52 nucleotides in *A. mitopteryx* and *P. antarctica* respectively). This region encodes for a polypeptide perfectly matching the C-terminus end of the corresponding apocytochrome B protein (fig. 6 and supplementary fig. S6, Supplementary Material online). The occurrence of the unambiguous remnant of *cob* strongly corroborates the rearrangement pathway, based on a TDPRL event, suggested as leading from VertGO to Noto1GO in *A. mitopteryx* and *P. antarctica* (fig. 4*a*). For a full description of all GR-ISP found in the 28 mitochondrial genomes analyzed in this study, see supplementary Results and Discussion,Supplementary Material online.

### The Inversion of the Control Region in Trematominae: Genomic Compositional Bias and Impact on the Phylogenetic Output

To test the effect of the inversion of the CoRe on the compositional bias of the mitogenomes of Trematominae, we initially computed the AT-skews and the GC-skews for the 13 PCGs, the ribosomal genes, and the whole mitogenomes (supplementary figs. S17–S23, Supplementary Material online). Subsequently, we tested with a Student’s*t*-test whether the absolute values of the AT-skews and the GC-skews in Trematominae were significantly different from all other notothenioids. The comparison resulted statistically significant (*P* < 0.01) when considering the full-length sequence of each PCG, the first and third codon positions only and the ribosomal genes. When considering the second positions of PCGs, results ranged from not significant (e.g., *atp8*; *P* ≥ 0.06) to highly significant differences (e.g., *nad2*; *P* ≪ 0.05). However, despite the significant differences, in many instances the sign of the skews was the same for Trematominae and for all other notothenioid fish (supplementary figs. S17–S23, Supplementary Material online). In general, the comparison of AT-skews and GC-skews across the entire dataset revealed that the CoRe inversion impacted the nucleotide composition of the mitogenomes and PCGs of Trematominae.

To verify if the CoRe inversion in Trematominae caused a global reversal of the strand compositional bias, the AT2-, GC2-skews and AT4-, GC4-skews were computed for the synonymous third positions of NNR2+NNY2 and NNN4 degenerate codons respectively, and the strand symmetry was tested for both A versus T and G versus C compositions (Hassanin et al. 2005). The AT2- and AT4-skews were negative for all Trematominae and positive for all other cryonotothenioid species. The A versus T strand symmetry for the synonymous third positions of NNR2+NNY2 set was rejected (*P* < 0.05) only in *Trematomus borchgrevinki*, whereas the A versus T strand symmetry of NNN4 set was rejected (*P* < 0.05) in all Trematominae. The GC2- and GC4-skews were negative for all cryonotothenioids including the Trematominae. The G versus C strand symmetry of the NNN4 set was rejected (*P* < 0.05) in all cryonotothenioids, whereas for the NNR2+NNY2 set, the G versus C strand symmetry was not rejected for the Trematominae (*P* > 0.05). In general, these results suggest that the CoRe inversion determined only a partial reversal of the compositional bias in the Trematominae.

To verify if the partial reversal of the compositional bias in Trematominae impacted the phylogenetic output, we performed phylogenetic analyses only using the mitochondrial genes (*28 T.Mito* dataset). The best partition scheme approach and the heterotachy evolutionary model (Chernomor et al. 2016; Crotty et al. 2019) generated the same topology, which was, in turn, identical to that obtained combining mitochondrial and nuclear genes (*28 T.Mito* + *28 T.Nucl* datasets) with the best partition scheme approach (supplementary fig. S1, Supplementary Material online). This tree is also in agreement with the most recent phylogeny of 80 notothenioid species based on thousand nuclear loci obtained using RADseq (Near et al. 2018).

Although our test for differences applied to the AT-skews and the GC-skews indicated that the inversion of CoRe in the mitogenomes of Trematominae significantly affected the nucleotide composition, our analysis of the AT2-, GC2-skews and AT4-, GC4-skews and the phylogenetic analysis lead to the conclusion that the inversion of the CoRe in Trematominae does not alter the phylogenetic output. However, we cannot exclude that the application of other evolutionary models would evidence an impact on the phylogeny of the switch of polarity caused by the inversion of the CoRe. Future work, based on a larger taxon coverage, especially of Trematominae, is needed to clarify the extent of the impact of the CoRe inversion on the mitochondrial phylogeny of Notothenioidei.

### Type of Selection Acting on Mitochondrial Protein-Coding Genes

**Fig. 7.**
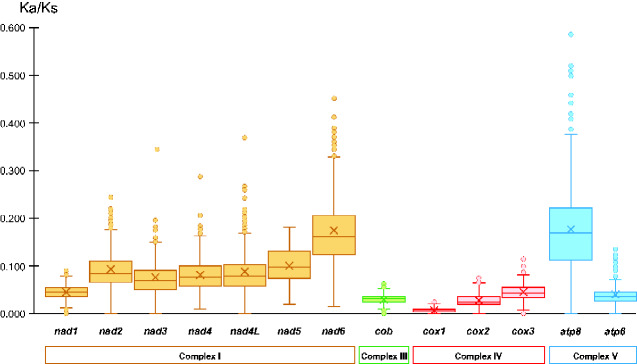
Box plot of the Ka/Ks pairwise ratios calculated for each mitochondrial protein-coding gene and organized based on the corresponding OXPHOS complex. Middle line, median value; x, mean; box upper and lower edges, interquartile range, including 50% of the observations; circles, outliers. Whisker lines extend for 1.5 times the interquartile range. Genes nomenclature as in figure 1. Complex I, III, IV, and V: complexes of the oxidative phosphorylation metabolic pathway containing the proteins encoded by the mitochondrial genes.

The analysis of the Ka/Ks ratios for the 13 mitochondrial PCGs shows that all genes evolved under purifying selection (fig. 7). The overall pattern of Ka/Ks variability in the 13 genes agrees with a previous analysis performed on a broad sampling of 401 fish mitogenomes (Sun et al. 2011, no notothenioids included). The mitochondrial genes of notothenioids have evolved under variable levels of purifying selection. The genes encoding proteins included in the complexes III and IV of the oxidative phosphorylation (OXPHOS) metabolic pathway have evolved under the strongest purifying selection, particularly *cox1*. Conversely, the genes coding for proteins of the complex I experienced a less pronounced purifying selection, particularly *nad6*. In general, among the 13 genes, *atp8* (complex V) evolved under the most relaxed conditions. In contrast, *atp6* shows a purifying selective pressure similar to genes of complexes III and IV. The protein-coding genes particularly involved in the rearrangements in cryonotothenioids are *nad1* (in Trematominae), *cob*, and *nad6*. Our analysis of the Ka/Ks ratios indicate that *cob* and *nad1* evolved under the effect of a purifying selection similarly to the genes not involved in the rearrangements (fig. 7). On the contrary, *nad6* is the second less constrained gene in terms of purifying selection after *atp8* (fig. 7).

To identify the occurrence of taxon-specific changes in the 13 PCGs and detect signatures of episodic diversifying selection or shifts in the stringency of selection (Smith et al. 2015; Wertheim et al. 2015), we tracked the evolution of each gene along selected branches of the two topologies obtained in our analysis. Since results were fully congruent for the two topologies, we report here only what we obtained for the tree based on the heterotachy approach (fig. 2 and supplementary fig. S36 and tables S6*a*, S6*b*, S7*a*, and S7*b* in Supplementary Material online).

The analysis performed with aBSREL identified the occurrence of four events of episodic diversifying selection (supplementary fig. S36 and tables S6*a* and S6*b*, Supplementary Material online) involving *cox3*, *nad3*, *nad5*, and *nad6*. Except for the root branch of Channichthyinae, the events of episodic diversifying selection were restricted to subterminal or terminal branches of the reference tree (e.g., the branch leading to node 16 for *nad6*, to node 18 for *cox3*, and to *R. glacialis* for *nad*3, supplementary fig. S36,Supplementary Material online).

The analyses performed with RELAX identified 18 and 24 events of intensification and relaxation of selection respectively (supplementary fig. S36 and tables S7*a* and S7*b*, Supplementary Material online). These events interested, at different extent, all genes except for *cox1* that is one of the genes that has evolved under the strongest purifying selection based on the Ka/Ks ratios analysis (for further detail see supplementary Results, section “Type of Selection Acting on Mitochondrial Protein-Coding Genes: A Phylogeny-Based Strategy”, Supplementary Material online).

All taxa tested with RELAX experienced events of intensification or relaxation of selection (supplementary fig. S36 and tables S7*a* and S7*b*, Supplementary Material online).

When the occurrence of the intensification or relaxation of selective pressure is analyzed at the combined taxonomic and gene level, it becomes evident that genes involved in multiple events experienced opposite types of selection in multiple taxa (supplementary fig. S36 and tables S7*a* and S7*b*, Supplementary Material online). For instance, in the evolution of *nad2*, the intensification of selection involved *N. coriiceps*, Artedidraconinae, *C. gunnari* and Channichthyinae, whereas a relaxation occurred in Nototheniidae, *P. antarctica*, Trematominae and *G. acuticeps.* Nonetheless, no difference in the selective pressure was found for *cob*, *nad1* and *nad6* to explain their role in the GO rearrangements.

When considering comprehensively the results of the analyses carried with aBSREL and RELAX and setting the cryonotothenioid clade (Nototheniidae) as a foreground group, we observed no indication of diversifying selection and a prevalence of relaxation of the selective pressure in comparison with the non-Antarctic notothenioids (for *cox3*, *cob*, *nad2*, *nad4*, and *nad6*; supplementary tables S6*a*, S6*b*, S7*a*, and S7*b*, Supplementary Material online). Similarly, early diverging taxa as the Dissostichinae, *Dissostichus* genus, *P. antarctica*, and *A. mitopteryx* experienced a relaxation of the selective pressure for several genes (supplementary tables S7*a* and S7*b*, Supplementary Material online). These results indicate that a relaxation of the selective pressure, under the prevailing direction of purifying selection, characterized the inception of the evolution of Nototheniidae.

On the contrary, events of diversifying selection and intensification of the selective pressure become more relevant in more recently diverged taxa. In particular in the subfamily Channichthyinae, the episodic diversifying selection was detected for *nad5*, whereas eight (*cox2*, *cox3*, *atp6*, *cob*, *nad2*, *nad4*, *nad5*, and *nad6*) of the 13 PCGs were subject to the intensification of the selection. The diversifying selection acting at the root of the Channichthyinae (*nad5*) suggests a boost of differentiation at the onset of the clade while subsequently the pressure of selection intensified during the cladogenetic process (for further detail see supplementary Results, section “Type of Selection Acting on Mitochondrial Protein-Coding Genes: A Phylogeny-Based Strategy”, Supplementary Material online).

Although no specific pattern of selection could be detected for the genes involved in the mitochondrial genomic rearrangements, it is evident that *nad6* is the most dynamic in terms of sequence divergence among the genes involved in the mitogenenome rearrangements (fig. 7). Although our analysis does not indicate that *nad6* has evolved under the pressure of diversifying selection at the gene level in the Nototheniidae (but only in Trematominae), we cannot exclude the possibility that positive selection occurred at single codons as previously suggested by Zhuang and Cheng (2010). Interestingly, Mark et al. (2012) reported evidence of increased protein flexibility, especially in *nad6* compared with*nad1* and *nad2* in Antarctic notothenioids. The analysis of the evolutionary behavior of single codons in the 13 PCGs of Nototheniidae remains an interesting venue for future research based on a larger taxon coverage.

In general, our results indicate that, the cryonotothenioid protein-coding genes, involved or not in mitogenome rearrangements, evolved under a common selective pressure. We can conclude that while purifying selection has been the prevailing selective force shaping the fate of PCGs to maintain function, a relaxation of selection enabled the early phase of Nototheniidae evolution, whereas a stronger selective pressure has shaped the diversification of more recent taxa possibly in parallel with the intensification of the polar environmental conditions. It is also possible that structurally, the genomic selection that modeled the gene order of cryonotothenioid mitogenomes was more relaxed and allowed several rearrangements.

### Mechanisms of Structural Evolution of Notothenioid Mitochondrial Genomes

Our results reveal that the structural evolution of the mitochondrial genome in the Nototheniidae is characterized by high plasticity and diversity and is particularly remarkable considering the young evolutionary age of the nototheniid clade. Based on our reconstruction, at least ten events of rearrangement (figs. 4 and 5) have characterized the evolution of the cryonotothenioid mitochondrial genome since the initial diversification of the family Nototheniidae (∼22 Ma, Dornburg et al. 2017) (fig. 3). The exceptionality of the nototheniid mitochondrial plasticity is further highlighted by the comparison with other fish families like the Bothidae and Myctophidae, for which gene order changes are documented although in fewer instances than in notothenioids. In the Bothidae (more than 160 species), 4 types of GO rearrangements characterize the 13 species sequenced to date (Luo et al. 2019). The Myctophidae (∼ 250 species), regarded as highly dynamic in terms of GO variability, exhibit only four mitochondrial rearrangements (Poulsen et al. 2013) compared with the 8 GOs found in the Nototheniidae which include ∼ 100 species.

The study of intergenic spacers was instrumental to unravel this plasticity, as proved by the characterization of gene orders in *D. eleginoides*, *G. acuticeps*, *A. mitopteryx*, and *P. antarctica* which result from rearrangement events that would have remained undisclosed if the analysis had been restricted to the genomic placements of functional genes. A few mechanisms have been proposed to explain GO rearrangements that involve large genomic regions in fish, as for instance the entire dimerization of the mitochondrial genome and, in some cases, nonrandom loss of genes based on their polarity. In particular, the DMNL mechanism proposed by Shi et al. (2013) for flatfishes requires two CoRe for the dimerization of the entire mitogenome to occur. The lack of direct evidence of a similar intermediate step in our dataset limits our power to exclude an analogous mechanism generating the GOs found in notothenioids with double CoRe. However, mechanisms similar to the DMNL involve the whole mitogenomes and, although often indirect, the evidence provided by the GR-ISPs found in the mitogenomes of Antarctic notothenioids suggests that in many cases the rearrangement occurred via a more parsimonious mechanisms of local duplication of a few genes and a subsequent random loss of the redundant copies.

Current evidence supports the view that the IQM and WANCY mitogenomic regions are hot spots of rearrangement in fish (e.g., Inoue et al. 2003; Miya et al. 2003; Satoh et al. 2016). Our analysis shows that the WANCY region is usually structurally stable in notothenioids and did not contribute to GO diversity. Instead, the IQM is involved in the inversion in Trematominae. Long-range mitogenomic inversions could be generated by intramitochondrial recombination (Dowton and Campbell 2001; Dowton et al. 2002; Li et al. 2019) as suggested to explain reverted GO in several vertebrates and invertebrates (Shi et al. 2013). Mechanisms that generate inversions require the double-stranded breakage of DNA on either side of the inverted region followed by subsequent reintegration of the excised fragment back into the circular genome in opposite orientation (Dowton and Campbell 2001; Brockman and McFadden 2012). If reintegration occurs at the original breakage point a local inversion is generated (Brockman and McFadden 2012) as would be the case of TremaGO. One possible explanation for the evolution of gene rearrangements by inversion of large, conserved blocks of genes is that the junctions between blocks may represent hotspots (IQM and CoRe for TremaGO) that promote the double-stranded breakage necessary to explain gene inversion (Dowton and Campbell 2001; Brockman and McFadden 2012). We speculate that the TDRL events occurred during the evolution of Noto1GO and of the very early ancestor of Trematominae (figs. 3 and 4*a*) may have favored errors in mtDNA replication enhancing the chance of DNA breakage and reconnection. This hypothesis could be tested in future studies by intraspecific mitogenomic surveys to verify the extent of intramitochondrial recombination (as in Xia et al. 2016) and by expanding the taxon coverage to verify whether yet-unsampled intermediate steps may corroborate the transition from Noto1GO to TremaGO via a TDRL event and inversion.

Certainly, a key role in the structural plasticity of the mtDNAs of the Nototheniidae was played by the Control Region since most of the changes affected the genes adjacent to the replication origin. Five of the nototheniid gene orders (Noto1GO, DissoGO, Noto2GO, RacoGO in *R. glacialis*, and ChamGO in *C. gunnari*) carry two copies of the CoRe, both possessing all the sequence motifs that characterize a true active Control Region (Zhuang and Cheng 2010). In this study, the phylogenetic analysis performed with the Control Regions revealed that, despite deriving from a common ancestor, the CoRe sequences are more closely related at intraspecific level than interspecifically because paralogs were always sister sequences with high bootstrap support (supplementary fig. S24, Supplementary Material online).

Two alternative hypotheses can be advanced to explain the phylogenetic output of the cryonotothenioid duplicated Control Regions. In the first hypothesis, the CoRe duplication occurred independently in every species. This explanation is challenged by the parsimonious reconstruction of the mitogenomic rearrangements occurred in the Nototheniidae (fig. 3). Moreover, the co-occurrence of multiple independent duplications of the CoRe is statistically very unlikely given the high number of cryonotothenioid species with two CoRe in their mitogenomes. As an alternative hypothesis, the high level of sequence identity (>90%) in the core CoRe segments (i.e., the conserved parts including the sequence-motifs characterizing an active CoRe, Zhuang and Cheng 2010) could be due to the homogenizing effect of concerted evolution that limits the nucleotide divergence of paralogous Control Regions (Li et al. 2015). However, since in our case duplicated paralogous CoRes are never completely identical, we suggest that a *relaxed* concerted evolution would better define the process at work. This second hypothesis is in perfect agreement with the parsimonious transformational pathway reconstructing the gene order evolution in cryonotothenioid mitogenomes (fig. 3). The concerted evolution hypothesis has been suggested in previous studies reporting duplicated Control Regions in various animal taxa (fish, Li et al. 2015; parrots, Eberhard et al. 2001; sea cucumbers, Arndt and Smith 1998; snakes, Kumazawa et al. 1996; and ticks, Shao et al. 2005). The duplicated CoRes, evolving under the constraints of concerted evolution, have two common features: 1) the high level of sequence identity, especially in their core segments responsible for effective folding, and 2) the similarity among paralogs is higher than among orthologs (Li et al. 2015). As a consequence, duplicated paralogous CoRes cluster together in a phylogenetic tree. Since the duplicated control regions of cryonotothenioids share these features, we consider this to result more likely from a (relaxed) concerted evolution rather than from multiple independent events of duplication.

From a structural point of view, the frequency of transition from a rearrangement with a duplicated CoRe to a single CoRe suggests that the condition with one Control Region may be more stable (fig. 3). A strong selective pressure that reduces the extra copies of duplicated regions and keeps a compact mitogenome with a constant number of genes may explain this tendency (Wolstenholme 1992).

Furthermore, a hotspot of gene rearrangement adjacent to origin of the H-strand replication (i.e., the Control Region) would frequently lead to homoplastic mitochondrial rearrangements (Xia et al. 2016). Under this condition, mitochondrial gene orders appear susceptible to convergent or parallel evolution (Xia et al. 2016). Indeed, notothenioid mitogenomes are characterized by several instances of parallel evolution that generated the same GOs (e.g., Noto2GO and Noto3GO). In this sense, the homoplastic nature of some gene rearrangements limits their use for reconstructing phylogenetic relationships. Nonetheless, our results show that mitogenomic rearrangements in notothenioids can occur also as a molecular signature. Specifically, TremaGO could be a molecular signature shared by all Trematominae. In fact, considering the high number of genes involved and the complexity of the rearrangement, it is highly unlikely that a gene order like TremaGO would be acquired independently and multiple times by unrelated clades.

### Structural Evolution of Mitochondrial Genomes: The Notothenioid Radiation and the Environmental Context

The disruption of the VertGO, possibly occurred in the common ancestor of Nototheniidae, opened up to a series of major GO changes within the Antarctic notothenioids located at the root of minor radiation events that occurred during the evolution of the clade (e.g., Trematominae, Dissostichinae, Channichthyinae). These events are reconstructed in coincidence with some periods of cooling of the Antarctic continent when a boost of speciation rate is also observed (e.g., Near et al. 2012; Dornburg et al. 2017).

This reconstruction brings up the hypothesis that GO rearrangements may have evolved under a relaxation of the structural constraints that usually so effectively prevent rearrangements in vertebrate mitochondrial genomes and that may have had a role in the process of adaptation. Our analysis shows that protein-coding genes of the notothenioid mitogenome experienced purifying selection in both standard and rearranged genomes, strongly preserving mitochondrial gene function. Therefore, the rate of nucleotide substitution seems to be unrelated to the propensity of mitogenomic rearrangements in notothenioids (as in other species, e.g., Irisarri et al. 2012; Kurabayashi and Sumida 2013; Xia et al. 2014). Our analysis also indicates that events of gene duplication are rapidly followed by deletion of the redundant gene-copies (some redundant genes remain only in rare GOs at terminal nodes, e.g., ChamGO, RacoGO, and GymnoGO, and usually remaining duplicated genes are *tRNA*s, e.g., Noto1GO). It may be speculated that a pre-existing genomic flexibility of the ancestor of the Nototheniidae, as proposed by Daane et al. (2019), may have generated a precondition for gene order rearrangement, whereas the strong pressure of purifying selection could have worked over short evolutionary timescales as mechanism for a rapid restoration of the mitochondrial functionality and compactness after each event of rearrangement. Subsequently, genetic drift may have driven the fixation and dispersal of mitogenomic reorganizations (Xia et al. 2016). Studies that investigate the signatures of these putative molecular and demographic mechanisms are required to clarify what factors led to the retention of the mitochondrial GO variability in cryonotothenioids. These studies should also address whether and how the climate history of Antarctica may have impacted the species’ demography and therefore the successful spread of rearrangements. Moreover, future research is also needed to investigate the impact of gene order rearrangements on mitochondrial functionality. Preliminary experiments with mitochondrial extracts of *N. rossii* and *N. coriiceps* lead by Mark et al. (2012) showed that mitochondrial function is preserved despite the rearranged gene orders (i.e., Noto1GO/Noto2GO). Additional studies should test other structural rearrangements to verify whether mitochondrial functionality differs among GO types.

In conclusion, this study documented some unique instances of structural diversity in the Antarctic notothenioid fish and demonstrates that while sequencing large nuclear genomes is still a complex task and not yet state-of-the-art, small compact mitochondrial genomes embed a large amount of unexpected information that advanced analytical approaches can bring to light.

## Supplementary Material

Supplementary data are available at *Genome Biology and Evolution* online.

## Author Contributions

C.P. and E.N. designed the study. C.P., M.B., A.D., A.B., L.H., C.B., F.M.H., and E.N. performed specimens collection, and/or molecular work and/or bionformatic analyses of the data. C.P. and E.N. wrote the first draft. A.D., M.L., L.H., and T.P. revised the manuscript. E.N. prepared the figures. All other authors contributed to edits and discussion.

## Supplementary Material

evab017_Supplementary_Data
