# Supplementary material for: Not Frozen in the Ice: Large and Dynamic Rearrangements in the Mitochondrial Genomes of the Antarctic Fish
Source: Genome Biol Evol. 2021 Feb 11;13(3):evab017. doi: 10.1093/gbe/evab017 (PMC7936035; doi:10.1093/gbe/evab017)
Supplement: evab017_Supplementary_Data [file evab017_supplementary_data.pdf]

Supplementary Material

Not frozen in the ice: large and dynamic rearrangements in the mitochondrial genomes of the Antarctic fish

Chiara Papetti[1,2], Massimiliano Babbucci[3], Agnes Dettai[4], Andrea Basso[3], Magnus Lucassen[5], Lars Harms[5,6], Celine Bonillo[7], Franz Maximilian Heindler[8], Tomaso Patarnello[3], and Enrico Negrisolo[3,9*]

[1]Department of Biology, University of Padova - 35121 Padova, Italy

[2]Consorzio Nazionale Interuniversitario per le Scienze del Mare (CoNISMa) - 00196 Roma, Italy

[3]Department of Comparative Biomedicine and Food Science, University of Padova - 35020 Legnaro, Italy

[4]Institut de Systematique, Evolution, Biodiversité (ISYEB) Muséum national d'Histoire naturelle-CNRS-Sorbonne Université-EPHE, MNHN – 75005 Paris, France

[5]Alfred Wegener Institute – Helmholtz Centre for Polar and Marine Research, Am Handelshafen 12, 27570 Bremerhaven, Germany

[6]Helmholtz Institute for Functional Marine Biodiversity at the University of Oldenburg (HIFMB), Ammerländer Herrstrasse 231, 26129 Oldenburg, Germany

[7]Service de Systématique Moléculaire, UMS2700 "Acquisition et Analyse de Données (2AD), MNHN – 75005 Paris, France

[8]Laboratory of Biodiversity and Evolutionary Genomics, KU Leuven, Leuven, Belgium

[9]CRIBI Interdepartmental Research Center for Innovative Biotechnologies, University of Padova, viale G. Colombo 3, 35121 Padua, Italy

***Corresponding author**: E-mail: enrico.negrisolo@unipd.it

Abstract

The vertebrate mitochondrial genomes generally present a typical gene order. Exceptions are uncommon and important to study the genetic mechanisms of gene order rearrangements and their consequences on mitochondrial function and phylogenetic output. Antarctic notothenioid fish carry some peculiar rearrangements of the mitochondrial gene order. In this first systematic study of 28 species, we analysed known and undescribed mitochondrial genome rearrangements for a total of eight different gene orders within the notothenioid fish. Our reconstructions suggest that transpositions, duplications and inversion of multiple genes are the most likely mechanisms of rearrangement in notothenioid mitochondrial genomes. In Trematominae, we documented an extremely rare inversion of a large genomic segment of 5300 bp that partially affected the gene compositional bias but not the phylogenetic output. The genomic region delimited by *nad5* and *trnF*, close to the area of the Control Region, was identified as the hot spot of variation in Antarctic fish mitochondrial genomes. Analysing the sequence of several intergenic spacers and mapping the arrangements on a newly generated phylogeny showed that the entire history of the Antarctic notothenioids is characterized by multiple, relatively rapid, events of disruption of the gene order. We hypothesised that a pre-existing genomic flexibility of the ancestor of the Antarctic notothenioids may have generated a precondition for gene order rearrangement, and the pressure of purifying selection could have worked for a rapid restoration of the mitochondrial functionality and compactness after each event of rearrangement.

Key words

mitochondrial genome evolution, gene order rearrangements, Notothenioidei, *Trematomus*, icefish, *Dissostichus*

Results and Discussion

The mitochondrial genomes of Notothenioidei

The gene order of all mitochondrial genomes analysed in this study is depicted in the supplementary figures S3-S5 (Supplementary Material online). The figures include all mitochondrial genomes sequenced *de novo* in this study and those available in GenBank reannotated here and retained for our analyses (supplementary table S2, Supplementary Material online). A detailed description of the gene orders and rearrangement pathways is reported in the main text. In the supplementary figures S3-S5 (Supplementary Material online), two types of spacers (STD-ISP and GR-ISP) are graphically differentiated in supplementary figs. S3-S5 (Supplementary Material online).

Determining the reference phylogenetic tree

The best tree obtained from the dataset *28T.Mito + 28T.Nucl*, by applying the heterotachy approach (HET-Tree) (fig. 2), differed from that obtained by applying the best partition scheme (BPS-Tree) for the placement of *Pleuragramma antarctica* (supplementary fig. S1, Supplementary Material online). In the HET-Tree, *P. antarctica* was sister taxon to all other Nototheniidae (fig. 2), while in the BPS-Tree, it was sister taxon of the clade formed by Dissostichinae + Trematominae. This latter alternative placement received limited statistical support (supplementary fig. S1, Supplementary Material online).
We compared our HET-Tree and BPS-Tree with the most recent and taxon rich phylogenies of Notothenioidei (Dornburg et al. 2017; Near et al. 2018). Our BPS-Tree is congruent with the “Maximum likelihood phylogeny of Cryonotothenioidea, inferred from RADseq dataset using IQ-Tree”, provided in figure 1 of Near et al. (2018). Conversely, the placement of *P. antarctica* in the HET-Tree is congruent with the “Species tree inferred using SVD quartets” presented in figure 2 of Near et al. (2018). In the phylogeny provided by Dornburg et al. (2017), and differently from both our topologies, *P. antartica* was sister species of Dissostichinae, and this clade was sister taxon to all other Nototheniidae included the Trematominae (other alternative arrangements can be found in Dettai et al. 2012).
In our study, we resolved to use the HET-Tree (fig. 2) as main reference topology to map the evolution of gene order in Notothenioidei, because we demonstrated that some level of heterogeneity exists in our datasets (supplementary figs. S25-S35, Supplementary Material online). However, given the uncertainty on the branching pattern of the most basal nodes of Nototheniidae (Dornburg et al. 2017; Near et al. 2018, and this study), we also reconstructed the evolution of mitochondrial gene order on the BPS-Tree (supplementary figs. S1-S2, Supplementary Material online) showing no difference with the results obtained by using the HET-Tree.

Intergenic spacers linked to genomic rearrangements

In the 28 mitochondrial genomes considered in this study, we identified several GR-ISPs. GR-ISPs were aligned with the corresponding genes involved in genomic rearrangements (figs. 3-6, and supplementary figs. S3-S5 and figs. S6-S16, Supplementary Material online). The results obtained by the analysis of these alignments provided strong support to the rearrangement mechanisms hypothesized as responsible for the evolution of the GOs observed in cryonotothenioids.

All the cryonotothenioid mitochondrial genomes with a gene order different from the VertGO show patterns of distribution of their GR-ISPs congruent with the hypothesized corresponding transformational pathways (figs. 3-5; supplementary figs. S3-S5, Supplementary Material online). Furthermore, several of the GR-ISPs exhibit, at variable degrees of sequence similarity, relicts of genes involved in the rearrangement (supplementary figs. S6-S16, Supplementary Material online).

The GR-ISPs between the *trnE* and $trnT_b$ (GR-ISPs $trnE\text{-}trnT_b$, hereafter the spacers will be indicated only by the names of the neighbouring genes) in both *Aethotaxis mitopteryx* and *P. antarctica* contain sequence remnants identical to portions of the active *cob* gene. Furthermore, portions of these GR-ISPs *trnE-trnTb* still encode for an amino acidic sequence identical to C-terminal portions of the CYTB protein (fig. 4; supplementary figs. S3 and S6, Supplementary Material online). The occurrence of these remnants strongly supports the hypothesized rearrangement pathway that led to the Noto1GO (fig. 4).

The GR-ISPs *trnP-nad6* found in Trematominae contains the remnant sequence of the 5’ upstream CoRe1, lost in the very early ancestor of this clade (fig. 2 and fig. 4a; supplementary fig. S3 and figs. S7-S9, Supplementary Material online).

The GR-ISP *trnP-nad6* in *Dissostichus eleginoides* contains a highly conserved pseudo-*trnE*, compared to the true *trnE*, but lacks the correct anticodon (TTT vs TTC) and has a mismatch pairing in the DHU arm and a reduced DHU loop (fig. 4b; and supplementary fig. S3 and figs. S10-S11, Supplementary Material online). Again, in *D. eleginoides*, the GR-ISP *trnP-nad6* includes also a relict CoRe2 particularly similar with the 5’ portion of the true CoRe2 (fig. 4b; and supplementary fig. S3 and fig S10, Supplementary Material online). The presence of these gene remnants in the GR-ISP *trnP-nad6* of *D. eleginoides* strongly supports a transformational pathway implying the duplication of the *trnE*-CoRe2 segment and the subsequent loss of its upstream copies (fig. 4b; and supplementary fig. S3 and figs. S10-S11, Supplementary Material online). Of the two *Dissostichus* species, only *D. eleginoides* carries the GR-ISP *trnP-nad6* (supplementary fig. S3, Supplementary Material online).

The GR-ISPs *trnT-nad6* in various Artedidraconinae, Bathydraconinae Cygnodraconinae, and Channichthyinae contains remnants of the CoRe1 (figs. 2, 3 and 5; supplementary fig. S4 and fig. S5, Supplementary Material online) (selected examples are provided in supplementary figs. S12-S14, Supplementary Material online). The level of sequence conservation between the spacer and the Control Region is different among the species considered in this study. The identification of these GR-ISPs as remnants of CoRe1 varies from indisputable (*Artedidraco skottsbergi, Akarotaxis nudiceps* and *Pagetopsis macropterus*)

to very good (*Gymnodraco australis* and *Pseudochaenichthys georgianus*) and plausible (*Dolloidraco longedorsalis*, *Neopagetopsis ionah*, *Chionodraco hamatus* and *Chionodraco rastrospinosus*). The occurrence of remnants of CoRe1 in *A. skottsbergi, A. nudiceps* and *P. macropterus* is unambiguous since the GR-ISP *trnT-nad6* and the CoRe of each species share exclusive sequence motifs: i.e. (1) a 71 bases segment long for *A. skottsbergi*; (2) a 93 bases long segment for *A. nudiceps*; (3) a 85 bases long segment for *P. macropterus*. The presence of these CoRe remnants in the GR-ISP *trnT-nad6* of Artedidraconinae, Bathydraconinae Cygnodraconinae, and Channichthyinae strongly supports the occurrence of a common ancestor exhibiting a Noto2GO in the cladogenetic pathway of these species (fig. 3). Thus, given the phylogenetic relationships among cryonotothenioids and the GOs distribution (figs. 2- 3; supplementary table S1 and supplementary figs. S1-S5, Supplementary Material online), it is evident that the Noto3GO appeared independently in Artedidraconinae and in the clade including Bathydraconinae, Gymnodraconinae, Cygnodraconinae and Channichthyinae (figs. 2-3; supplementary figs. S1-S2, Supplementary Material online).

The GR-ISP *trnT-trnE*$_a$ of *G. acuticeps* is an example of the most complex GO rearrangement in this study (fig. 5; supplementary fig. S4 and figs. S14-S15, Supplementary Material online). This GR-ISP includes (from 5' to 3'): (1) a segment A that is identical to the 5' start of *nad6*; (2) a pseudo-*trnE*$_a$; (3) two segments B-C, which encode peptides exhibiting good level of identity/similarity with portions of the NAD6 protein. The pseudo-*trnE*$_a$ shows a good level of conservation with the true *trnE*$_a$ (supplementary fig. S15, Supplementary Material online). However, the pseudo-*trnE*$_a$ does not contain the right anti codon (TTT vs TTC). Furthermore, this pseudo-tRNA shows multiple mismatch-pairings in the acceptor and TΨC stems. Finally, extra bases, which would prevent the formation of the tRNA secondary structure, are located at the 5' side of the acceptor stem, as well as in the anticodon loop (supplementary fig. S15, Supplementary Material online). The segments B and C encode for peptides that can be reasonably interpreted as remnants of NADH6 (supplementary fig. S15, Supplementary Material online). The distribution pattern of the remnants found in this GR-ISP *trnT-trnE*$_a$ is consistent with, and reasonably explained by the triplication of the genomic segment *trnE-nad6*, and the successive pseudogenization/loss of two *nad6* and one *trnE* copies (fig. 5). Finally, the GR-ISP *trnP*$_b$*-trnF* of *G. acuticeps* shares the unique, eighteen base long, motif TACACACATCAGTACAGC with the CoRe (supplementary fig. S16, Supplementary Material online), therefore strongly supporting the view that this spacer is a degenerated remnant of the Control Region (fig. 5; supplementary fig. S4, Supplementary Material online).

For some GR-ISPs, it was not possible to identify remnants of the gene(s) involved in the genomic rearrangement. The GR-ISP *nad5-cob* is a paradigmatic case: this spacer is present in all cryonotothenioid mitochondrial genomes sequenced so far, but it never carries remnants that can be indisputably identified as relicts of *nad6* and/or *trnE*.

In some other cases, it was not possible to identify the boundaries of GR-ISPs (e.g. GR-ISP *trnT*-CoRe1 in the Noto2GO) usually because these spacers are limited by a CoRe. Thus, we cannot exclude that some GR-ISP are inside the CoRe and the lack of conserved motifs clearly associated to gene remnants has prevented us from finding them.

Type of selection acting on mitochondrial protein-coding genes: a phylogeny-based strategy

To identify the occurrence of taxon-specific changes in the 13 PCGs and detect signatures of episodic diversifying selection or shifts in the stringency of selection (Smith et al. 2015, Wertheim et al. 2015), we tracked the evolution of each gene along selected branches of the two topologies obtained in our analysis (fig. 2; supplementary fig. S1, Supplementary Material online). Since results were fully congruent for the two topologies, we report here only what we obtained for the tree based on the heterotachy approach (fig. 2, supplementary fig. S36, Supplementary Material online).

Thirteen taxa and their associated branches were tested using the programs aBSREL and RELAX (available at the DataMonkey 2.0 web site, Smith et al. 2015; Wertheim et al. 2015; Weaver et al. 2018). The taxa are: (1) the family Nototheniidae (cryonotothenioid clade), (2) *Pleuragramma antarctica*, (3) the subfamily Dissostichinae, (4) *Aethotaxis mitopteryx*, (5) the *Dissostichus* genus, (6) the subfamily Trematominae, (7) *Notothenia coriiceps*, (8) *Harpagifer antarcticus*, (9) the subfamily Artedidraconinae, (10) the subfamily Bathydraconinae, (11) *Gymnodraco acuticeps*; (12) the subfamily Channichthyinae and (13) *Champsocephalus gunnari* (supplementary fig. S36 Supplementary Material online).

A detailed description of the strategy applied to obtain the results presented here is provided in the paragraph "Type of selection acting on mitochondrial protein-coding genes: a phylogeny-based strategy" (supplementary Materials and Methods, Supplementary Material online).

The analysis performed with aBSREL identified the occurrence of four events of episodic diversifying selection (supplementary fig. S36 and tables S6a and S6b, Supplementary Material online). The four events affected: (a) the branch leading to node 18, i.e., the last common ancestor of *Trematomus eulepidotus* + *Trematomus tokarevi* for *cox3*; (b) the branch leading to *Racovitzia glacialis* for *nad*3; (c) the branch leading to node 37, the root of Channichthyinae for *nad5*; (d) the branch leading to node 16, the root of the genus *Trematomus* for *nad6*. Except for the Channichthyinae, the events of episodic diversifying selection were restricted to sub-terminal or terminal branches of the reference tree.

The analyses performed with RELAX identified the occurrence of 18 and 24 events of intensification (int) and relaxation (rel) of selection respectively (supplementary fig. S36 and tables S7a and S7b, Supplementary Material online). At gene level, these events interested, at different extent, all genes except for *cox1*. A single event of intensification interested *cox2*, *atp8* and *nad4L*. *Atp6* (1int/1rel), *nad1* (2rel) and *nad3* (2rel) experienced two events. *Nad5* was interested by three events (2int/1rel). Four events affected both *cox3* (2int/2rel) and *cob* (1int/3rel). Seven events occurred in *nad4* (3int/4rel) and *nad6* (2int/5rel). *Nad2* experienced eight events (4int/4rel).

At taxonomic scale, all 13 taxa analysed with RELAX experienced events of intensification or relaxation of selection (supplementary fig. S36 and tables S7a and S7b, Supplementary Material online). The Artedidraconinae were interested by a single event of intensification. *A. mitopteryx* and *H. antarcticus* experienced a single episode of relaxation. Both *P. antarctica*, and *G. acuticeps* experienced two events of relaxation. The Bathydraconinae were affected by one event of intensification (for *atp8*) and one of relaxation

of selection (for *nad4*). Three events of intensification characterized *C. gunnari*. *N. coriiceps* experienced two events of intensification and one event of relaxation. The genus *Dissostichus* exhibited four events of relaxation. Both Dissostichinae and Nototheniidae experienced five events of relaxation. The Trematominae were affected by three events of intensification and two of relaxation. The subfamily Channichthyinae experienced eight events of intensification (supplementary fig. S36 and table S7a and S7b, Supplementary Material online).

When the occurrence of the intensification or relaxation of selective pressure is analysed at the combined taxonomic and gene level, it becomes evident that genes involved in multiple events experienced opposite types of selection in multiple taxa (supplementary fig. S36 and tables S7a and S7b, Supplementary Material online). For instance, in the evolution of *nad2*, the intensification of selection involved *N. coriiceps,* Artedidraconinae, *C. gunnari* and Channichthyinae, while a relaxation occurred in Nototheniidae, *P. antarctica*, Trematominae and *G. acuticeps*. Nonetheless, no difference in the selective pressure was found for *cob*, *nad1* and *nad6* to explain their role in the GO rearrangements.

When considering comprehensively the results of the analyses carried with aBSREL and RELAX and setting the cryonotothenioid clade (Nototheniidae) as a foreground group, we observed no indication of diversifying selection and a prevalence of relaxation of the selective pressure in comparison with the non-Antarctic notothenioids (for *cox3*, *cob*, *nad2*, *nad4*, and *nad6*; supplementary tables S6a-S6b and S7a-S7b, Supplementary Material online). Similarly, early diverging taxa as the Dissostichinae, *Dissostichus* spp., *P. antarctica*, and *A. mitopteryx* experienced a relaxation of the selective pressure for several genes (supplementary tables S7a and S7b, Supplementary Material online). These results indicate that a relaxation of the selective pressure, under the prevailing direction of purifying selection, characterized the inception of the evolution of Nototheniidae.

On the contrary, events of diversifying selection and intensification of the selective pressure become more relevant in more recently diverged taxa. In particular in the subfamily Channichthyinae, the episodic diversifying selection was detected for *nad5*, while eight (*cox2*, *cox3*, *atp6*, *cob*, *nad2*, *nad4*, *nad5* and *nad6*) of the 13 PCGs were subject to the intensification of the selection. The diversifying selection acting at the root of the Channichthyinae (*nad5*) suggests a boost of differentiation at the onset of the clade while subsequently the pressure of selection intensified during the cladogenetic process. This hypothesised evolutionary scenario is supported by the aBSREL/RELAX scores obtained for the branches of Channichthyinae (supplementary tables S6b and S7b, Supplementary Material online). Indeed, *nad5* appears to have evolved under a relaxation of the selective pressure along the branch leading to node 37, i.e., the root of Channichthyinae, a result fully consistent with the event of episodic diversifying selection identified by aBSREL. Conversely, the set of branches emerging from node 37 (leading to all icefish species analysed in this study, supplementary fig. 36 and tables S7a and S7b, Supplementary Material online) have experienced an intensification of the selective pressure based on analysis with RELAX.

Although no specific pattern of selection could be detected for the genes involved in the mitochondrial genomic rearrangements, it is evident that *nad6* is the most dynamic in terms of sequence divergence among

the genes involved in the mitogenenome rearrangements (fig. 7). Although our analysis does not indicate that *nad6* has evolved under the pressure of diversifying selection at the gene level in the Nototheniidae (but only in Trematominae), we cannot exclude the possibility that positive selection occurred at single codons as previously suggested by Zhuang and Cheng (2010). Interestingly, Mark et al. (2012) reported evidence of increased protein flexibility, especially in *nad6* compared to *nad1* and *nad2* in Antarctic notothenioids. The analysis of the evolutionary behaviour of single codons in the 13 PCGs of Nototheniidae remains an interesting venue for future research based on a larger taxon coverage.

In general, our results indicate that, the cryonotothenioid protein coding genes, involved or not in mitogenome rearrangements, evolved under a common selective pressure. We can conclude that while purifying selection has been the prevailing selective force shaping the fate of PCGs to maintain function, a relaxation of selection enabled the early phase of Nototheniidae evolution while a stronger selective pressure has shaped the diversification of more recent taxa possibly in parallel with the intensification of the polar environmental conditions. It is also possible that structurally, the genomic selection that modelled the gene order of cryonotothenioid mitogenomes was more relaxed and allowed several rearrangements.

Materials and Methods

Biological sample collection and handling

Specimens sequenced in this study (supplementary table S1, Supplementary Material online) were collected in 2012 during the PS79 Polarstern cruise in the Antarctic Peninsula, with the exception of the specimens of *Trematomus eulepidotus* collected in 2013-2014 during the PS82 Polarstern cruise in the Weddell Sea, the *T. borchgrevinki* Pe1912 (CE000005560) collected during the REVOLTA2 survey in Terre Adélie, *A. mitopteryx* 1159 and *Bovichtus angustifrons* collected at Fossil Island Tasmania (Australia). All specimens were obtained by bottom trawl or hand netting and tissues are stored in ethanol 95% at +4°C either at the Biology Department of the University of Padova (Italy), at the Muséum national d'Histoire naturelle (MNHN, Paris) or at the Alfred Wegener Institute Helmholtz Centre for Polar and Marine Research (AWI, Bremerhaven, Germany).

Sample collection conducted on specimens obtained during Polarstern cruises were approved by the competent national authority for Antarctic research (Umweltbundesamt, UBA). Animal experimentation at AWI was in accordance with German law and approved by a competent national authority (Freie Hansestadt Bremen, Germany, permit number AZ: 522-27-11/02- 00 (93)). Ethical approval for all procedures obtained during the fieldwork of the REVOLTA2 program was granted by the ethics committee of the Ministère de l'Environnement and the French Polar Research Institute (Institut Paul Emile Victor – IPEV). The experiments complied with the Code of Ethics of Animal Experimentation in the Antarctic sector. Collection of *B. angustifrons* sample was supported under Australian Antarctic Science Project 4030 'Assessment and monitoring of fisheries in Australia's Antarctic and sub-Antarctic Territories in the Indian Ocean sector of the Southern Ocean', and an Australian Antarctic Ethics Committee permit.

DNA extraction

The final dataset of fifteen complete and two partial mitochondrial genomes (mtDNAs) sequenced *de novo* for this study (supplementary table S1 and table S2, Supplementary Material online) is the result of a collaborative effort. Samples handling, DNA extraction and sequencing were performed in different laboratories and with different kits (supplementary table S1, Supplementary Material online). For the mitochondrial genomes obtained in Italy (at the Department of Comparative Biomedicine and Food Science of the University of Padova), DNA for all samples to be sequenced by Illumina platform was extracted using the ZR Genomic DNATissue Midiprep Kit (Zymo Research corp.), while DNA for all samples to be Sanger sequenced was isolated using the Invisorb DNA extraction kit (Invitec). DNA quality was assessed through electrophoresis. The DNA concentration was determined using the high sensitivity Qubit DNA quantification kit (Invitrogen, USA). Mitochondrial genomes of *T. borchgrevinki* and *A. mitopteryx* were obtained in Paris at the Service de Systématique Moléculaire (UMS2700 OMSI of the Muséum National d'Histoire Naturelle). Extractions were performed using the CTAB protocol of Winnepenninckx et al. (1993). DNA quality was assessed through electrophoresis. The sequences used to *de novo* assemble the mitochondrial genome of *L. nudifrons* were obtained from transcriptomic data published in Papetti et al. (2015).

Genome sequencing

All mtDNA samples extracted in Italy were sent to UCDAVIS Genome Center (http://dnatech.genomecenter.ucdavis.edu/; Davis University, California) at a minimum concentration of 100 ng/ml, where they were sequenced on an Illumina HiSeq 2500 via Rapid Run Mode — PE100 paired-end technology. The partial sequences of *Harpagifer antarcticus* and *Racovitzia glacialis* were generated through standard PCR with universal primers following Papetti et al. (2007) and sequenced by Sanger technology at the BMR Genomics service (http://www.bmr-genomics.it/) on automated DNA sequencers. The complete mitochondrial genomes of *T. borchgrevinki* and *A. mitopteryx* were obtained by long PCR (Hinsinger et al. 2015) with new primers designed for notothenioids (supplementary table S4, Supplementary Material online). Control Region of *A. mitopteryx* was amplified and sequenced in two fragments overlapping on *nad6*. The PCR products overlap by more than 500 bp in the 16S, by the whole *COI* (>1600 bp), >100 bp in *nad6*, and over the whole *nad4*, *nad4L*, and *nad3* (2300 bp). Multiplex sequencing followed the protocol of Hinsinger et al. (2015).

Genome assembly

The Sanger chromatograms were assembled using the DNASTAR package (Madison, WI) and revised by eye. The Illumina reads were assembled *de novo* using the CLC Genomics Workbench 8.5 (Qiagen, Hilden, Germany). The MITObim v. 1.8 program (Hahn et al. 2013) was used to complete the assembly of some

sequences as described in Basso et al. (2017). For *T. borchgrevinki* and *A. mitopteryx* assembly followed Hinsinger et al. (2015) using the assembler of Geneious R10.2.2 (Biomatters Ltd). Both *de novo* and baiting strategies were applied and compared. Separate sequencing of the four long PCR products allowed to check for the gene order. The coverage for the mitochondrial genomes sequenced by the Italian team with the Illumina technology varied from a minimum value of 141X (24,544 reads for *A. skottsbergi*) to a maximum value of 1074X (191,982 reads for *N. ionah*). The coverage for the mitochondrial genomes sequenced by the French team was 157X for *T. borchgrevinki* and 272X for *A. mitopteryx*.

Mitochondrial genome annotation

The last and corresponding author annotated all newly sequenced mtDNAs. The nomenclature of genes follows Montelli et al. (2016). In literature, there is large variability in the names used to indicate mtDNA strands (Basso et al. 2017). In this paper, we adopted the code H-strand to denote the strand encoding most genes (Satoh et al. 2016). The opposite strand is the L-strand (Satoh et al. 2016). Annotation of the new genomes (complete and partial) was performed according to the strategy described in Montelli et al. (2016).
Initially, the mtDNA sequence was translated into putative proteins using the Transeq (Madeira et al. 2019) program available on the EBI website (https://www.ebi.ac.uk/Tools/st/emboss_transeq/). The identity of these polypeptides was verified using the BLAST program available at the NCBI website (Altschul et al. 1990; Tatusova and Madden 1999). The boundaries of genes were determined by defining the 5′ ends of protein-coding genes (PCGs) as the first legitimate in-frame start codon (ATN, GTG, TTG, GTT, following the vertebrate mitochondrial genetic code) in the open reading frame (ORF) that was not located within an upstream gene encoded on the same strand. The only exceptions were *atp6* and *nad4*, which overlap with the final codon of their upstream genes (*atp8* and *nad4L*, respectively) in many mtDNAs (Wolstenholme 1992) and also in the notothenioids. The PCG terminus was defined as the first in-frame stop codon that was encountered. When the stop codon was located within the sequence of a downstream gene encoded on the same strand, a truncated stop codon (T or TA) adjacent to the beginning of the downstream gene was designated as the termination codon. This codon is completed by polyadenylation, thereby producing a complete TAA stop codon after transcript processing. Finally, pairwise comparisons with orthologous proteins were performed using ClustalW (Thompson et al. 1994) to better define the limits of the PCGs. Regardless of the real initiation codon, a formyl-Met was assumed to be the starting amino acid for all proteins as previously reported in other mitochondrial genomes (Smith and Marcker 1968; Fearnley and Walker 1987).
Transfer RNA genes were identified using the tRNAscan-SE program (Lowe and Eddy 1997) or recognized manually as sequences having the appropriate anticodon and capable of folding into the typical cloverleaf secondary structure of tRNAs. The validity of these predictions was further enhanced by comparison to published orthologous fish sequences and based on multiple alignment and secondary structure information. The boundaries of the ribosomal *rrnS* and *rrnL* genes were those defined by the tRNAs adjacent upstream/downstream to these genes (i.e., *trnF* and *trnV* for *rrnS*; *trnV* and *trnL2* for *rrnL*). The Antarctic

notothenioid mtDNAs may present (i) two distinct complete Control Regions (CoRe), both containing a full set of conserved regulatory sequence motifs (TAS, cTAS, ETAS, and the CSBs), or (ii) one complete and one incomplete (Zhuang and Cheng 2010 and this study). The occurrence of the regulatory motifs (Zhuang and Cheng 2010) was assessed for all the genomic portions identified as putative Control Regions and for the intergenic spacers.

To ensure a homogenous annotation for the full set of studied genomes, the mitogenomes already available in GenBank were reannotated following the same approach described above for the mitochondrial genomes sequenced in this study.

Taxon selection and mitochondrial genomes available from GenBank

All taxa analyzed in this work are listed in supplementary table S2 (Supplementary Material online). The 28 species included here cover all major notothenioid clades. Five mitochondrial genomes available in GenBank and belonging to the four species *T. bernacchii*, *T. borchgrevinki* (2 GenBank entries), *T. pennellii* and *Parachaenichthys charcoti* were excluded from the final dataset (supplementary table S2, Supplementary Material online). All Trematominae sequenced in our study share a common and highly peculiar gene order (TremaGO) which is described in detail in the main text. To check for the correctness of the genomic arrangement of the TremaGO, we performed long PCR assays and sequenced all PCR fragments. Several individuals were amplified for *T. borchgrevinki*.

The GenBank mitochondrial genomes of *T. bernacchii* (KU166863), *T. borchgrevinki* (KX025131 and KU951144) and *T. pennellii* (MK007073), available at the time of our analysis, are incomplete and present a gene order characteristic of the non-Trematominae that could not be reproduced through long PCR. In particular (a) the KX025131 sequence of *T. borchgrevinki* is incomplete and the genes *nad6* and *trnE* are missing. (b) Likely owing to known assembly complexity, the CoRe of the KU951144 sequence of *T. borchgrevinki* is incomplete and lacks many of the sequence motifs that characterize a true active Control Region (Zhuang and Cheng, 2010). (c) The KU166863 sequence of *T. bernacchii* presents a genomic arrangement where an incomplete *nad6* is located immediately upstream to a poor quality sequence of *rrnS*, while *trnE*, *trnF* and a true CoRe are lacking. (d) The MK007073 of *T. pennellii* is arranged in a single chimeric consensus and does not include *nad6*, *trnE*, *trnF* and a true CoRe. The first three GenBank mitochondrial genomes (*T. bernacchii* KU166863 and *T. borchgrevinki* KX025131 and KU951144) relied on Sanger sequencing of short PCR products while the last (*T. pennellii* MK007073) is assembled from Illumina reads.

The sequence of *P. charcoti* available in GenBank (KP300644.1) contains a region of 3388 bp (spanning from position 12141 to position 15528) identical to a sequence assigned to *Racovitzia glacialis* (GU214226 in Zhuang and Cheng, 2010). The GU214226 sequence in *R. glacialis* covers a mitochondrial portion ranging from the 3' end of *cob* and 5' end of *rrnS* (12S). In *P. charcoti*, the portion identical to *R. glacialis* extends from *trnT* to 5' end of *rrnS*. The sequence identity could be due to (a) a chimeric assembly of the KP300644 sequence that includes nucleotides from *P. charcoti* and *R. glacialis*, (b) a case of taxon misidentification for

the sequence GU214226, which may belong to *P. charcoti* instead of *R. glacialis*. We sequenced independently an almost complete mitochondrial genome of *R. glacialis*, but for the CoRe region and adjacent genes (i.e. the genomic segment spanning from *cob* to *trnF*; supplementary table S2, supplementary fig. S4, Supplementary Material online). Our specimen belongs to *R. glacialis* based on morphological identification during sampling and further corroborated by the analysis of the *cox1* DNA barcode in the BOLD system. Similarly, a BOLD search, based on the *cox1* from the *P. charcoti* KP300644, assigned unambiguously this sequence to *P. charcoti*. Phylogenetic analyses (data not shown) were performed on single orthologous genes alignments, including data from *P. charcoti* KP300644 and from our new sequenced *R. glacialis* genome (supplementary table S2, Supplementary Material online) with the IQ-TREE 1.6.9 software (Nguyen et al. 2015), to determine the position of *P. charcoti*. Single genes for *P. charcoti* were extracted from the mitochondrial sequence KP300644. These analyses were performed according to the maximum likelihood method. The IQ-TREE 1.6.9 software was used to select the best fitting-evolutionary model for each analysis (Nguyen et al. 2015). In all analyses, with the exclusion of *nad6* alignment, *P. charcoti* KP300644 was sister species of *Gerlachea australis,* while *R. glacialis* was sister taxon of *A. nudiceps* in agreement with other studies (e.g., Near et al. 2018). The phylogenetic analysis performed with *nad6* extracted from KP300644 (*P. charcoti*) and GU214226 (*R. glacialis*), which are identical despite being identified as two different species, resulted in a sister group relationship with *A. nudiceps*. Based on these observations, we assumed that the sequence KP300644 (*P. charcoti*) may have a chimeric origin and we discarded it from our final dataset.

Single gene datasets for phylogenetic analysis

For all 28 taxa listed in supplementary table S2 (Supplementary Material online), we created one orthologous gene dataset for each of the 13 mitochondrial protein coding genes, 2 mitochondrial ribosomal genes and 22 mitochondrial tRNAs. The tRNAs alignments were successively discarded because tRNAs contained limited amount of phylogenetic signal (data not shown but available upon request).

We prepared orthologous gene dataset also for 8 nuclear markers that have been used in several previous phylogenetic studies (e.g. Dettai et al. 2012; Dornburg et al. 2017) (supplementary table S2 and table S3, Supplementary Material online). These markers were selected among the nuclear sequences accessible in GenBank at the time of our phylogenetic analysis, because they were available for most of the target species of this study (table S2, Supplementary Material online). The goal of this approach was to minimize the amount of missing data. There were no sequences available for the 8 nuclear markers for *B. angustifrons* and *B. argentinus* (supplementary table S2, Supplementary Material online). We used instead the corresponding nuclear sequences obtained from congeneric taxa (*Bovichtus diacanthus* and *Bovichtus variegatus*, see supplementary table S3, Supplementary Material online) as a usual strategy used to make up for the absence of species-specific markers (e.g., Geisler et al. 2011). Some nuclear genes belong to the online dataset of Dornburg et al. (2017) as indicated in supplementary table S3 (Supplementary Material online), because at the time of production of our data and results the sequences were not yet available in GenBank. Some mtDNA

sequences were incomplete (supplementary table S2, Supplementary Material online), in this case, the missing genes were coded as question marks.

Single gene/protein multiple alignments

Each set of the orthologous protein-coding genes was aligned using MAFFT (Katoh et al. 2002; Katoh et al. 2005) on the TranslatorX server (Abascal et al. 2010). This web tool ensures that the alignment of DNA sequences is based on the multiple alignment derived from the amino acid translations as a reference. The non-coding genes were instead aligned with the online version of MAFFT ver. 7 (https://mafft.cbrc.jp/alignment/server/). We did not filter alignments to select blocks of conserved positions, because this process can produce incorrect but statistically supported trees (Tan et al. 2015). The single gene multiple alignments were imported in the MEGA5 software for successive bioinformatic manipulations and input file creation (Tamura et al. 2011).

Assessing the compositional biases and substitution pattern

The GC-skew = (G-C)/(G+C) and AT-skew = (A-T)/(A+T) (Perna and Kocher 1995) were used to measure the base compositional difference between the two strands or among genes coded on the alternative strands. The base compositions were computed with MEGA5 (Tamura et al. 2011). The skews were calculated with Excel (Microsoft TM). The software AliGROOVE was used to check for the overall level of compositional heterogeneity, a major source of misleading phylogenetic outputs, for every multiple alignment (Kück et al. 2014). Briefly, AliGROOVE calculates a pairwise mean similarity score between each pair of sequences (-1 ≤ range ≤ 1) and returns a matrix of colours. In the matrix, each square is a pairwise comparison between two sequences and the colour varies from deep blue (i.e. non-random similarity, +1) to deep brown (i.e. full random similarity, -1). A red/brown square indicates that heterogeneous aligned positions dominate between the two sequences, while a blue square indicates homogeneous positions (Kück et al. 2014). The AliGROOVE matrices were computed for the non-coding DNA sequences, for the complete codons, as well as for the first plus the second positions of each codon, for the second positions only, for the third positions only, and for the translated amino acid sequence for each of the marker alignments (supplementary figs. S25-S35, Supplementary Material online).

The third positions of many PCGs exhibited the most heterogeneous substitution patterns, where heterotachous sites were dominant (supplementary figs. S25-S35, Supplementary Material online) (Lopez et al. 2002). The substitution process was the most homogenous for the protein alignments. However, some of the protein alignments were not fully homogenous in their substitution pattern (e.g. ATP8, TBR1) (supplementary fig. S27 and fig. S34, Supplementary Material online). Irrespective to the analysed gene/protein, the heterogeneous substitution patterns were detected mostly in the pairwise-comparisons of the Bovichtidae and the

Eleginopsidae with the Nototheniidae sequences. Among all Nototheniidae analysed in this study, the heterotachy process in third codon position was more evident in the Trematominae.

Estimation of the phylogenetic signal

The phylogenetic signal in each single marker alignment (DNA non-coding, three codon positions, first two positions, second codon positions, last codon position and amino acids) was estimated by a likelihood mapping approach (Strimmer and von Haeseler, 1997) implemented in the IQ-TREE 1.6.9 program (Nguyen et al. 2015). We considered the percentage of Fully Resolved Quartets (%FRQ) over the total number of analysable unique quartets as an estimation of the phylogenetic signal, i.e. the higher the percentage the stronger the signal (Strimmer and von Haeseler 1997).

The percentage of FRQ was very low for the second positions of some nuclear markers (e.g. *myh6*, supplementary fig. S33, Supplementary Material online) due to the extreme level of conservation of these genes within notothenioids. The highest FRQ percentages were obtained for whole codons, proteins and third positions in several mitochondrial genes. In general, all analysed datasets exhibited some phylogenetic signal (supplementary figs. S25-S35, Supplementary Material online) thus all positions and markers were retained in the final concatenated dataset (see next section "Phylogenetic analysis").

Concatenation of genes/proteins

Once estimated the level of heterogeneity of the substitution process and the phylogenetic signal assessed, the single gene multiple alignments were concatenated in two multi-markers datasets. The first dataset, named *28T.Mito*, contained 15 mitochondrial genes (13 PCGs, *rrnS* and *rrnL*) and was 13791 positions long. The second dataset, named *28T.Nucl*, included the eight nuclear markers listed in supplementary table S3 (Supplementary Material online) and was 6619 positions long.

Phylogenetic analyses

Phylogenetic reconstructions were performed on *28T.Mito* and *28T.Nucl* datasets using IQ-TREE 1.6.9 (Nguyen et al. 2015). We ran multiple maximum likelihood analyses with different search strategies. We first worked with *28T.Mito* and *28T.Nucl* datasets separately and ran analyses based on multiple partitioning schemes, to use a big array of evolutionary models, but assuming the homogeneity of the substitution process (Chernomor et al. 2016). Selection of the best fitting evolutionary models to different genes/proteins was performed with the ModelFinder algorithm (Kalyaanamoorthy et al. 2017) implemented in IQ-TREE. Then the phylogenetic analyses were performed with the combined *28T.Mito* + *28T.Nucl* dataset, by applying the best partition scheme identified by IQ-TREE 1.6.9 (supplementary table S5, Supplementary Material online) (Chernomor et al. 2016). To accommodate the level of heterogeneity detected for several markers (as described

above), heterotachous models were applied to the combined dataset for the phylogenetic reconstruction (Crotty et al. 2019). The rationale behind this second tree-search strategy was that even a low level of heterogeneity in the substitution process can produce wrong topologies if the dataset is not properly analyzed through heterotachy models (Crotty et al. 2019). For further details on the final phylogenetic topologies associated to the different tree-search strategies see the ”Determining the reference phylogenetic tree” paragraph in the supplementary Results and Discussion section (Supplementary Material online).
In each analysis, ten runs were performed to minimize the risk of selecting suboptimal trees. Statistical support to the nodes of best trees was computed by ultrafast bootstrap test (10,000 replicates) (Hoang et al. 2018).
Finally, a phylogenetic analysis was performed on a multiple alignment generated with MAFFT ver. 7, including only the Control Regions. The tree search analysis (supplementary fig. S24, Supplementary Material online) was performed by applying the TN+F+I+G4 model selected by the ModelFinder algorithm (Kalyaanamoorthy et al. 2017) implemented in IQ-TREE.

Gene order evolution

The evolution of gene order (GO) in the notothenioid mtDNAs was mapped on the reference phylogenies (fig 2 and fig. 3; supplementary fig. S1 and fig. S2, Supplementary Material online). Since, to our best knowledge, there are no computer programs to infer complex rearrangement pathways in genomes with a variable number of genes as those studied here, the onset of events leading to each GO was reconstructed manually and placed at internal nodes under a maximum parsimony approach. We incorporated the results obtained from the analysis of the sequences of the intergenic spacers linked to genomic rearrangements into the gene order reconstruction (supplementary Results and Discussion, Supplementary Material online). The inclusion of these results provided robust support to the reconstruction of genomic transformational pathways presented in the main text. The available models of mitochondrial rearrangements were used as guidelines to infer the pathways of the genomic rearrangements (Moritz et al. 1987; Boore 2000; Dowton and Campbell 2001; Basso et al. 2017).

Dates of key nodes in the reference phylogeny tree

We associated dates to some of the nodes linked with the onset of the genomic rearrangements described in the main text. The chronogram in Dornburg et al. (2017) was used as reference for the dating. Geological Epoch/Age naming follows Ogg et al. (2016).

Type of selection acting on mitochondrial protein-coding genes: a phylogeny-based strategy

To test for selection across branches of a reference topology based on an alignment of orthologous genes, we applied the phylogeny-based strategy implemented in the programs aBSREL (adaptiveBranch-Site RandomEffects Likelihood) and RELAX (available at the DataMonkey 2.0 web site, https://www.datamonkey.org/, Smith et al. 2015; Wertheim et al. 2015; Weaver et al. 2018). The program aBSREL tests for the occurrence of events of episodic diversifying selection along single selected branches of a topology (Smith et al. 2015). The program RELAX tests whether the strength of natural selection has been relaxed or intensified along a specified set of branches (Wertheim et al. 2015). The aBSREL and RELAX differ in their statistical outputs. aBSREL identifies only the test branch or branches that experienced an episodic diversifying selection and estimates the relative statistical significance while RELAX calculates the p-value and whether the test was significant or not for the entire set of test branches.

Initially, we used as reference topologies the two trees obtained in this study (based on the heterotachy approach and on the best partition scheme, see section "A multi-gene phylogeny for the suborder Notothenioidei") and compared results. Since results obtained for the two topologies were in agreement, the analyses performed with aBSREL and RELAX are described below only for the heterotachy tree (fig. 2 and supplementary fig. S36, Supplementary Material online). Both aBSREL and RELAX require that a set of test branches (the foreground branches) are selected to be compared with some reference branches (the background branches). In this study, the test branches were selected among those along which changes in gene order occurred or that lead to cryonotothenioid clades containing two or more species. Each set of test branches was analysed in independent runs. The tested taxa and the corresponding test branches were (supplementary fig. S36, Supplementary Material online):

(1) The family Nototheniidae (cryonotothenioid clade). Test branches: the branch leading to node 5 and all branches emerging from it (green box in supplementary fig. S36, Supplementary Material online). Reference branches: branches leading to node 3 and to non-Antarctic species *E. maclovinus*, *B. argentinus* and *B. angustifrons*.

(2) *Pleuragramma antarctica*. Test branch: the branch emerging from node 5 and leading to *P. antarctica*. Reference branches: all other branches.

(3) The subfamily Dissostichinae. Test branches: the branch leading to node 9 and all branches emerging from it (orange box in supplementary fig. S36, Supplementary Material online). Reference branches: all other branches.

(4) *Aethotaxis mitopteryx*. Test branch: the branch emerging from node 9 and leading to *A. mitopteryx*. Reference branches: all other branches.

(5) The *Dissostichus* genus. Test branches: the branch leading to node 11 and all branches emerging from it (inside the orange box in supplementary fig. S36, Supplementary Material online). Reference branches: all other branches.

(6) The subfamily Trematominae. Test branches: the branch leading to node 14 and all branches emerging from it (cyan box supplementary fig. S36, Supplementary Material online). Reference branches: all other branches.

(7) *Notothenia coriiceps*. Test branch: the branch emerging from node 21 and leading to *N. coriiceps*. Reference branches: all other branches.

(8) *Harpagifer antarcticus*. Test branch: the branch emerging from node 24 and leading to *H. antarcticus*. Reference branches: all other branches.

(9) The subfamily Artedidraconinae. Test branches: the branch leading to node 26 and all branches emerging from it (purple box in supplementary fig. S36, Supplementary Material online). Reference branches: all other branches.

(10) The subfamily Bathydraconinae. Test branches: the branch leading to node 30 and all branches emerging from it (blue box in supplementary fig. S36, Supplementary Material online). Reference branches: all other branches.

(11) *Gymnodraco acuticeps*. Test branch: the branch emerging from node 33 and leading to *G. acuticeps*. Reference branches: all other branches.

(12) The subfamily Channichthyinae. Test branches: the branch leading to node 37 and all branches emerging from it (light-brown box in supplementary fig. S36, Supplementary Material online). Reference branches: all other branches.

(13) *Champsocephalus gunnari*. Test branch: the branch emerging from node 37 and leading to *C. gunnari*. Reference branches: all other branches.

References

Abascal F, Zardoya R, Telford MJ. 2010. TranslatorX: multiple alignment of nucleotide sequences guided by amino acid translations. Nucleic Acids Res. 38:W7-W13.

Altschul SF, Gish W, Miller W, Myers EW, Lipman DJ. 1990. Basic local alignment search tool. J Mol Biol. 215:403-410.

Basso A, Babbucci M, Pauletto M, Riginella E, Patarnello T, Negrisolo E. 2017.The highly rearranged mitochondrial genomes of the crabs *Maja crispata* and *Maja squinado* (Majidae) and gene order evolution in Brachyura. Sci Rep. 7:4096.

Boore JL. 2000. The duplication/random loss model for gene rearrangement exemplified by mitochondrial genomes of Deuterostome animals. In Sankoff D, Nadeau JH editors. Comparative genomics: Empirical and analytical approaches to gene order dynamics, map alignment and the evolution of gene families. Netherlands: Kluwer Academic Publishers. pp 133-147.

Chernomor O, von Haeseler A, Minh BQ. 2016. Terrace aware data structure for phylogenomic inference from supermatrices. Syst Biol. 65:997-1008.

Crotty SM, Minh BQ, Bean NG, Holland BR, Tuke J, Jermiin LS, Von Haeseler A. 2019. GHOST: Recovering Historical Signal from Heterotachously-evolved Sequence Alignments. Syst Biol. 69:249-264.

Dettai A, Berkani M, Lautredou AC, Couloux A, Lecointre G, Ozouf-Costaz C, Gallut C. 2012. Tracking the elusive monophyly of nototheniid fishes (Teleostei) with multiple mitochondrial and nuclear markers. Mar Genomics 8:49-58.

Dornburg A, Federman S, Lamb AD, Jones CD, Near TJ. 2017. Cradles and museums of Antarctic teleost biodiversity. Nat Ecol Evol. 1:1379-1384.

Dowton M, Campbell NJH. 2001. Intramitochondrial recombination – is it why some mitochondrial genes sleep around? Trends Ecol Evol. 16:269-271.

Fearnley IM, Walker JE. 1987. Initiation codons in mammalian mitochondria: differences in genetic code in the organelle. Biochemistry 26:8247-8251.

Geisler JH, McGowen MR, Yang G, Gatesy J. 2011. A supermatrix analysis of genomic, morphological, and paleontological data from crown Cetacea. BMC Evol Biol. 11:112.

Hahn C, Bachmann L, Chevreux B. 2013. Reconstructing mitochondrial genomes directly from genomic next-generation sequencing reads—a baiting and iterative mapping approach. Nucleic Acids Res. 41:e129

Hinsinger DD, Debruyne R, Thomas M, Denys GPJ, Mennesson M, Utge J, Dettai A. 2015. Fishing for barcodes in the Torrent: from COI to complete mitogenomes on NGS platforms. DNA Barcodes 3:170-186.

Hoang DT, Chernomor O, von Haeseler A, Minh BQ, Vinh LS. 2018. UFBoot2: improving the ultrafast bootstrap approximation. Mol Biol Evol. 35:518-522.

Kalyaanamoorthy S, Minh BQ, Wong TKF, von Haeseler A, Jermiin LS. 2017. ModelFinder: fast model selection for accurate phylogenetic estimates. Nat Methods 14:587-589.

Katoh K, Misawa K, Kuma K, Miyata T. 2002. MAFFT: a novel method for rapid multiple sequence alignment based on fast Fourier transform. Nucleic Acids Res. 30:3059-3066.

Katoh K, Kuma K, Toh H, Miyata T. 2005. MAFFT version 5: improvement in accuracy of multiple sequence alignment. Nucleic Acids Res. 33:511-518.

Kück P, Meid SA, Gross C, Wagele JW, Misof B. 2014. AliGROOVE--visualization of heterogeneous sequence divergence within multiple sequence alignments and detection of inflated branch support. BMC Bioinformatics 15:294.

Lopez P, Casane D, Philippe H. 2002. Heterotachy, an important process of protein evolution. Mol Biol Evol. 19:1-7.

Lowe TM, Eddy SR. 1997. tRNAscan-SE: A program for improved detection of transfer RNA genes in genomic sequence. Nucleic Acids Res. 25:955-964.

Madeira F, Park YM, Lee J, Buso N, Gur T, Madhusoodanan N, Basutkar P, Tivey ARN, Potter SC, Finn RD, et al. 2019. The EMBL-EBI search and sequence analysis tools APIs in 2019. Nucleic Acids Res. 47:W636-W641.

Montelli S, Peruffo A, Patarnello T, Cozzi B, Negrisolo E. 2016. Back to water: signature of adaptive evolution in cetacean mitochondrial tRNAs. Plos One 11:e0158129.

Moritz C, Dowling TE, Brown WM. 1987. Evolution of animal mitochondrial DNA: relevance for population biology and systematics. Annu Rev Ecol Evol S. 18:269-292.

Near TJ, MacGuigan DJ, Parker E, Struthers CD, Jones CD, Dornburg A. 2018. Phylogenetic analysis of Antarctic notothenioids illuminates the utility of RADseq for resolving Cenozoic adaptive radiations. Mol Phylogenet Evol. 129:268-279.

Nguyen LT, Schmidt HA, von Haeseler A, Minh BQ. 2015. IQ-TREE: a fast and effective stochastic algorithm for estimating maximum-likelihood phylogenies. Mol Biol Evol. 32:268-274.

Ogg JG, Ogg GM, Gradstein FM. 2016. A Concise Geologic Time Scale. Amsterdam: Elsevier.

Papetti C, Liò P, Rüber L, Patarnello T, Zardoya R. 2007. Antarctic fish mitochondrial genomes lack ND6 gene. J Mol Evol. 65:519-528.

Papetti C, Harms L, Windisch HS, Frickenhaus S, Sandersfeld T, Jürgens J, Koschnick N, Knust R, Pörtner HO, Lucassen M. 2015. A first insight into the spleen transcriptome of the notothenioid fish *Lepidonotothen nudifrons*: Resource description and functional overview. Mar Genomics 24:237-239.

Perna NT, Kocher TD. 1995. Patterns of nucleotide composition at fourfold degenerate sites of animal mitochondrial genomes. J Mol Evol. 41:353-358.

Satoh TP, Miya M, Mabuchi K, Nishida M. 2016. Structure and variation of the mitochondrial genome of fishes. BMC Genomics 17:719.

Smith AE, Marcker KA. 1968. N-formylmethionyl transfer RNA in mitochondria from yeast and rat liver. J Mol Biol. 38:241-243.

Smith MD, Wertheim JO, Weaver S, Murrell B, Scheffler K, Kosakovsky Pond SL. 2015. Less is more: an adaptive branch-site random effects model for efficient detection of episodic diversifying selection. Mol Biol Evol. 32:1342–1353.

Strimmer K., von Haeseler A. 1997. Likelihood-mapping: a simple method to visualize phylogenetic content of a sequence alignment. Proc Natl Acad Sci. USA 94:6815-6819.

Tamura K, Peterson D, Peterson N, Stecher G, Nei M, Kumar S. 2011. MEGA5: molecular evolutionary genetics analysis using maximum likelihood, evolutionary distance, and maximum parsimony methods. Mol Biol Evol. 28:2731-2739.

Tan G, Muffato M, Ledergerber C, Herrero J, Goldman N, Gil M, Dessimoz C. 2015. Current methods for automated filtering of multiple sequence alignments frequently worsen single-gene phylogenetic inference. Syst Biol. 64:778-791.

Tatusova TA, Madden TL. 1999. BLAST 2 Sequences, a new tool for comparing protein and nucleotide sequences. FEMS Microbiol Lett. 174:247-250.

Thompson JD, Higgins DG, Gibson, TJ. 1994. CLUSTAL W: improving the sensitivity of progressive multiple sequence alignment through sequence weighting, position-specific gap penalties and weight matrix choice. Nucleic Acids Res. 22:4673-4680.

Weaver S, Shank SD, Spielman SJ, Li M, Muse SV, Kosakovsky Pond SL. 2018. Datamonkey 2.0: a modern web application for characterizing selective and other evolutionary processes. Mol Biol Evol. 35:773–777.

Wertheim JO, Murrell B, Smith MD, Kosakovsky Pond SL, Scheffler K. 2015. RELAX: detecting relaxed selection in a phylogenetic framework. Mol Biol Evol. 32:820–832.

Winnepenninckx B, Backeljau T, De Wachter R. 1993. Extraction of high molecular weight DNA from molluscs. Trends Genet. **9**:407.

Wolstenholme DR. 1992. Animal mitochondrial DNA: structure and evolution. Int Rev Cytol. 141:173-216.

Zhuang X, Cheng CHC. 2010. ND6 gene “lost” and found: evolution of mitochondrial gene rearrangement in Antarctic notothenioids. Mol Biol Evol. 27:1391-1403.

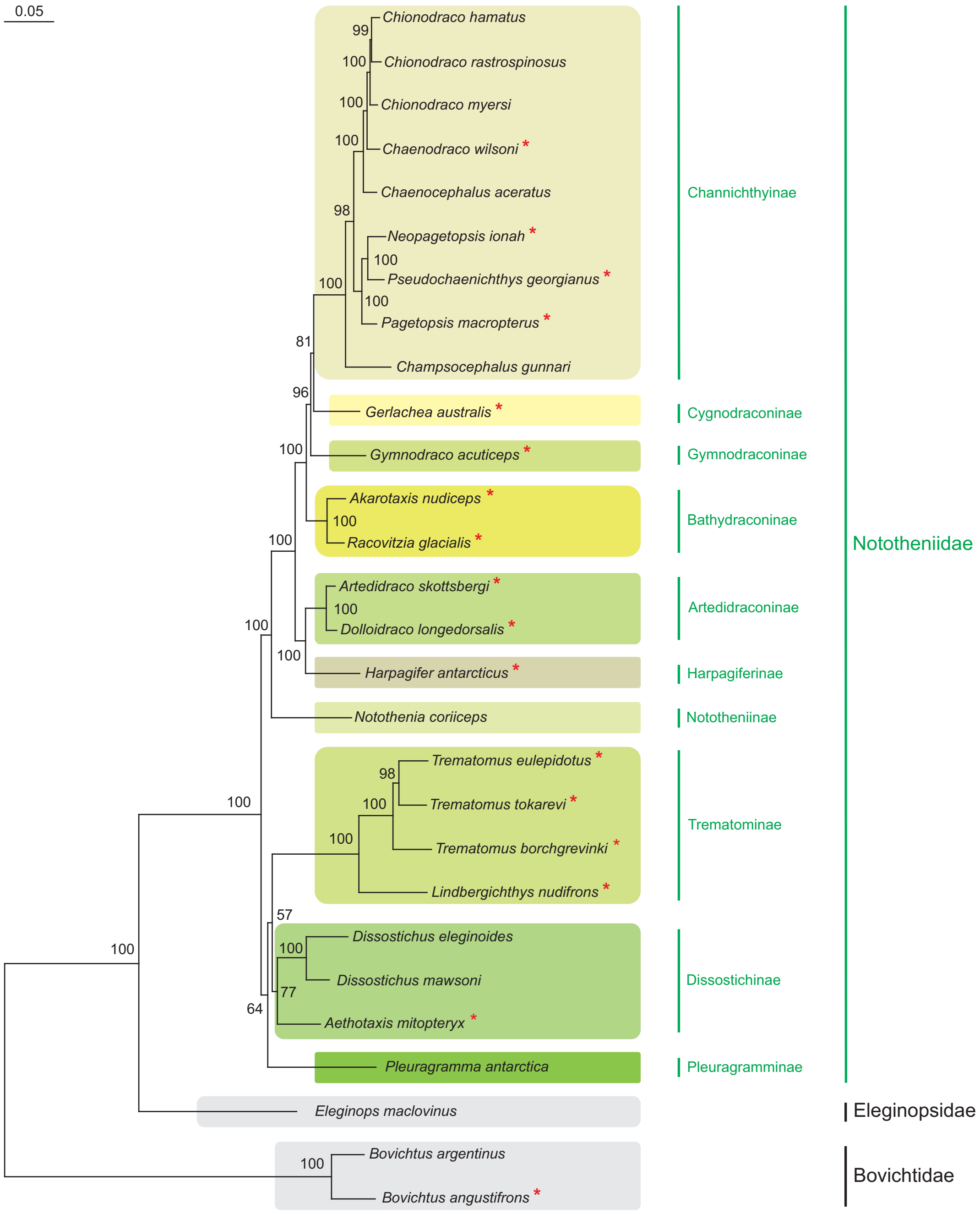

Supplementary Figure S1. Maximum Likelihood tree (-ln = 132097.4294) obtained from the analysis performed on the *28T.Mito* + *28T.Nucl* multiple alignments by applying the best partition scheme to the two data sets. Ultrafast bootstrap values are provided for each node. The scale bar represents 0.5 substitutions/site.* newly sequenced mtDNA. (See Supplementary Materials and Methods for details on the phylogenetic strategy).

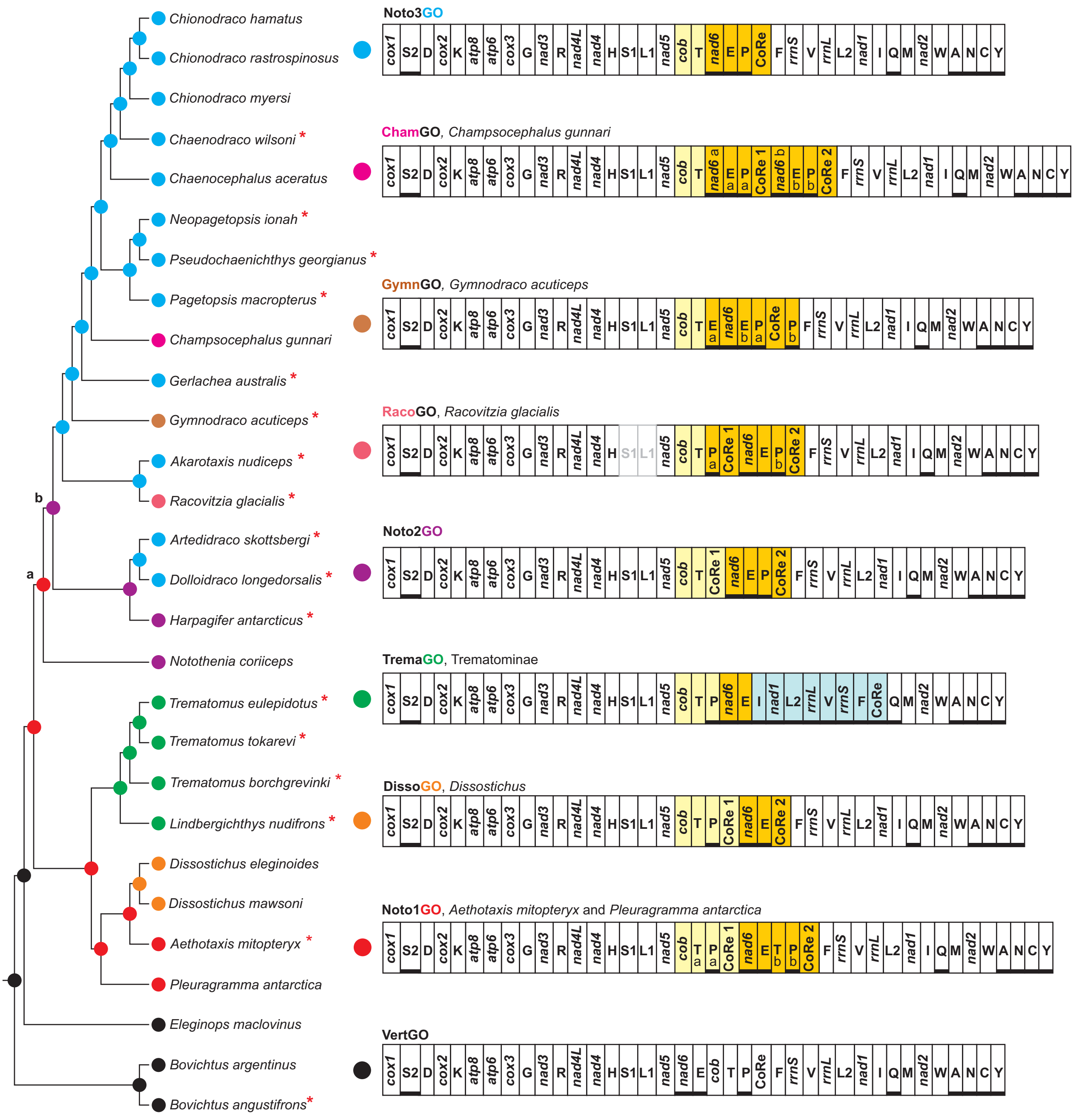

Supplementary Figure S2. Mitochondrial gene order evolution mapped on the phylogeny of Notothenioidei obtained by applying a best partition scheme to *28T.Mito* + *28T.Nucl* multiple alignments (supplementary fig. S1, Supplementary Material online). The evolution of mitochondrial gene order is mapped on the reference phylogenetic tree. Every gene order is indicated with GO and is linearized starting from *cox1*. Genes encoded on the H-strand are black-boxed, while those encoded on the L-strand are underlined and black-boxed. Nomenclature: *atp6* and *atp8*: genes coding for ATP synthase subunits 6 and 8; *cob*: apocytochrome b; *cox1-3*: cytochrome c oxidase subunits 1–3; *nad1-6* and *nad4L*: NADH dehydrogenase subunits 1–6 and 4L; *rrnS* and *rrnL*: small and large subunit ribosomal RNA (rRNA) genes; X: transfer RNA (tRNA) genes, where X is the one-letter abbreviation of the corresponding amino acid, in particular L1 (CTN codon family) L2 (TTR codon family), S1 (AGN codon family) S2 (TCN codon family); CoRe: Control Region. Nodes a and b carry an unresolved gene order. Genes transposed/duplicated with respect to the VertGO have a yellow (gene belonging to the 5' duplicated block) and orange (gene belonging to the 3' duplicated block) background, while genes translocated and moved on the L-strand have a light blue background. Genomes sequenced de novo in this study are indicated by an asterisk. Missing regions in incompletely sequenced genomes (*Racovitzia glacialis*) are in light-gray.

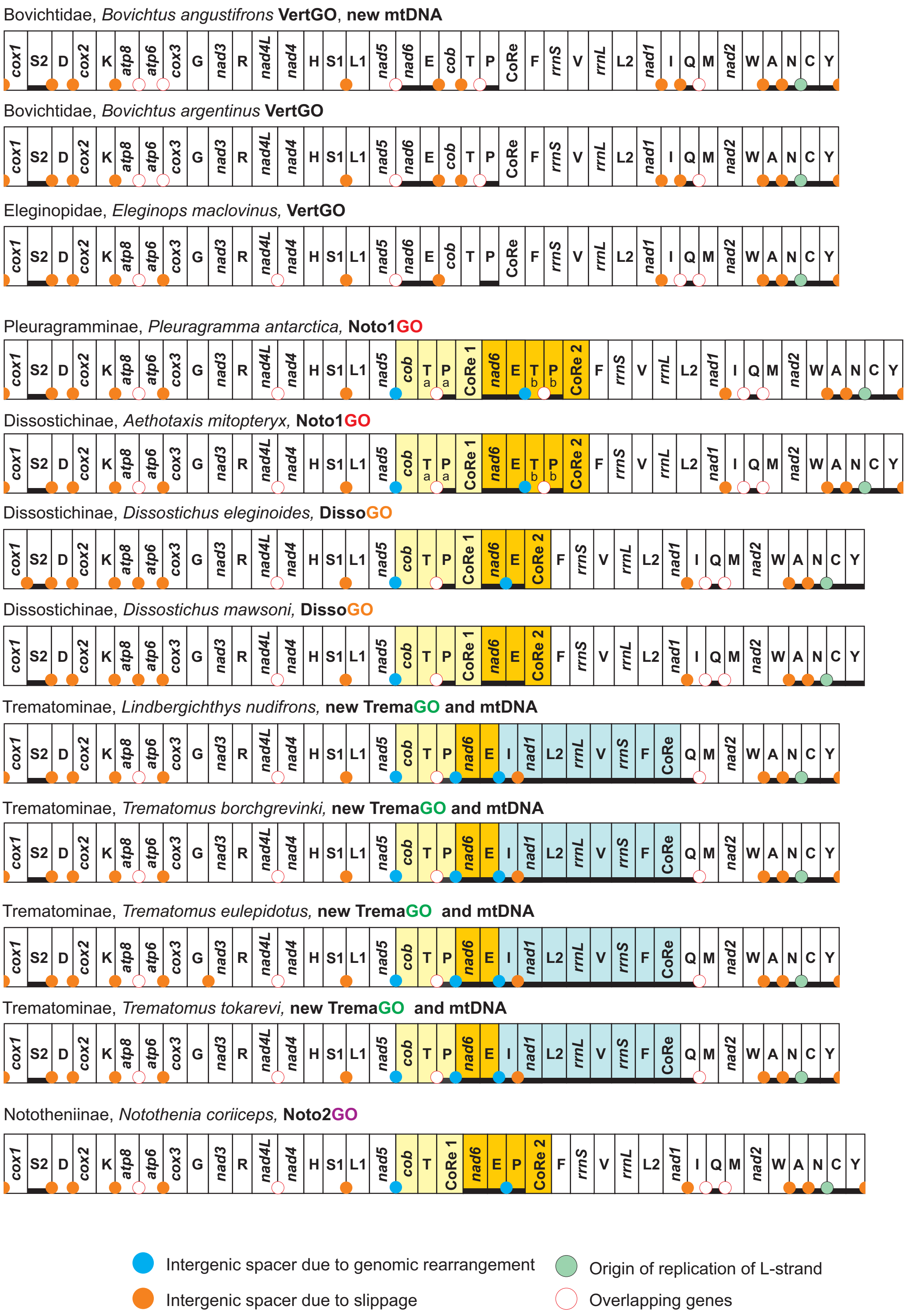

Supplementary Figure S3. Gene Orders in Notothenioidei (1). Every GO is linearized starting from *cox1*. The genes encoded on the H-strand (orientation from left to right in supplementary fig. S3) are black-boxed, while those encoded on the L-strand (orientation from right to left in supplementary fig. S3) are underlined and black-boxed. Nomenclature: *atp6* and *atp8*: ATP synthase subunits 6 and 8; *cob*: apocytochrome b; *cox1-3*: cytochrome c oxidase subunits 1–3; *nad1-6* and *nad4L*: NADH dehydrogenase subunits 1–6 and 4L; *rrnS* and *rrnL*: small and large subunit ribosomal RNA (rRNA) genes; X: transfer RNA (tRNA) genes, where X is the one-letter abbreviation of the corresponding amino acid, in particular L1 (CTN codon family) L2 (TTR codon family), S1 (AGN codon family) S2 (TCN codon family); CoRe: Control Region. Genes transposed/duplicated with respect to the VertGO have a yellow (gene belonging to the 5' duplicated block) and orange (gene belonging to the 3' duplicated block) background, while genes translocated and moved on the L-strand have a light blue background.

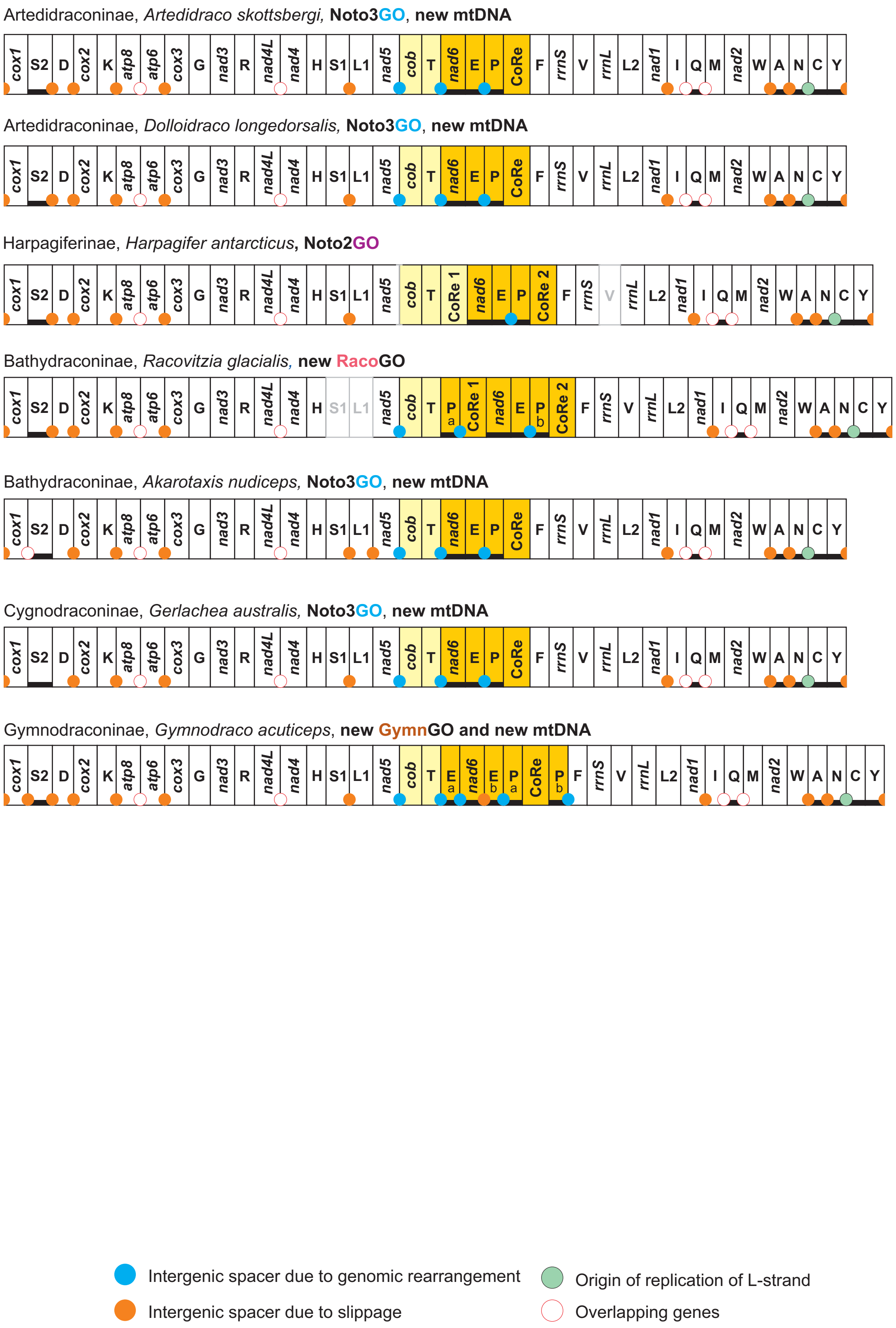

Supplementary Figure S4. Gene Orders in Notothenioidei (2). Every GO is linearized starting from *cox1*. The genes encoded on the H-strand (orientation from left to right in supplementary fig. S4) are black-boxed, while those encoded on the L-strand (orientation from right to left in supplementary fig. S4) are underlined and black-boxed. Nomenclature: *atp6* and *atp8*: ATP synthase subunits 6 and 8; *cob*: apocytochrome b; *cox1-3*: cytochrome c oxidase subunits 1–3; *nad1-6* and *nad4L*: NADH dehydrogenase subunits 1–6 and 4L; *rrnS* and *rrnL*: small and large subunit ribosomal RNA (rRNA) genes; X: transfer RNA (tRNA) genes, where X is the one-letter abbreviation of the corresponding amino acid, in particular L1 (CTN codon family) L2 (TTR codon family), S1 (AGN codon family) S2 (TCN codon family); CoRe: Control Region. Genes transposed/duplicated with respect to the VertGO have a yellow (gene belonging to the 5' duplicated block) and orange (gene belonging to the 3' duplicated block) background, while genes translocated and moved on the L-strand have a light blue background. Missing genes in the incomplete genome of *Racovitzia glacialis* are in light-grey.

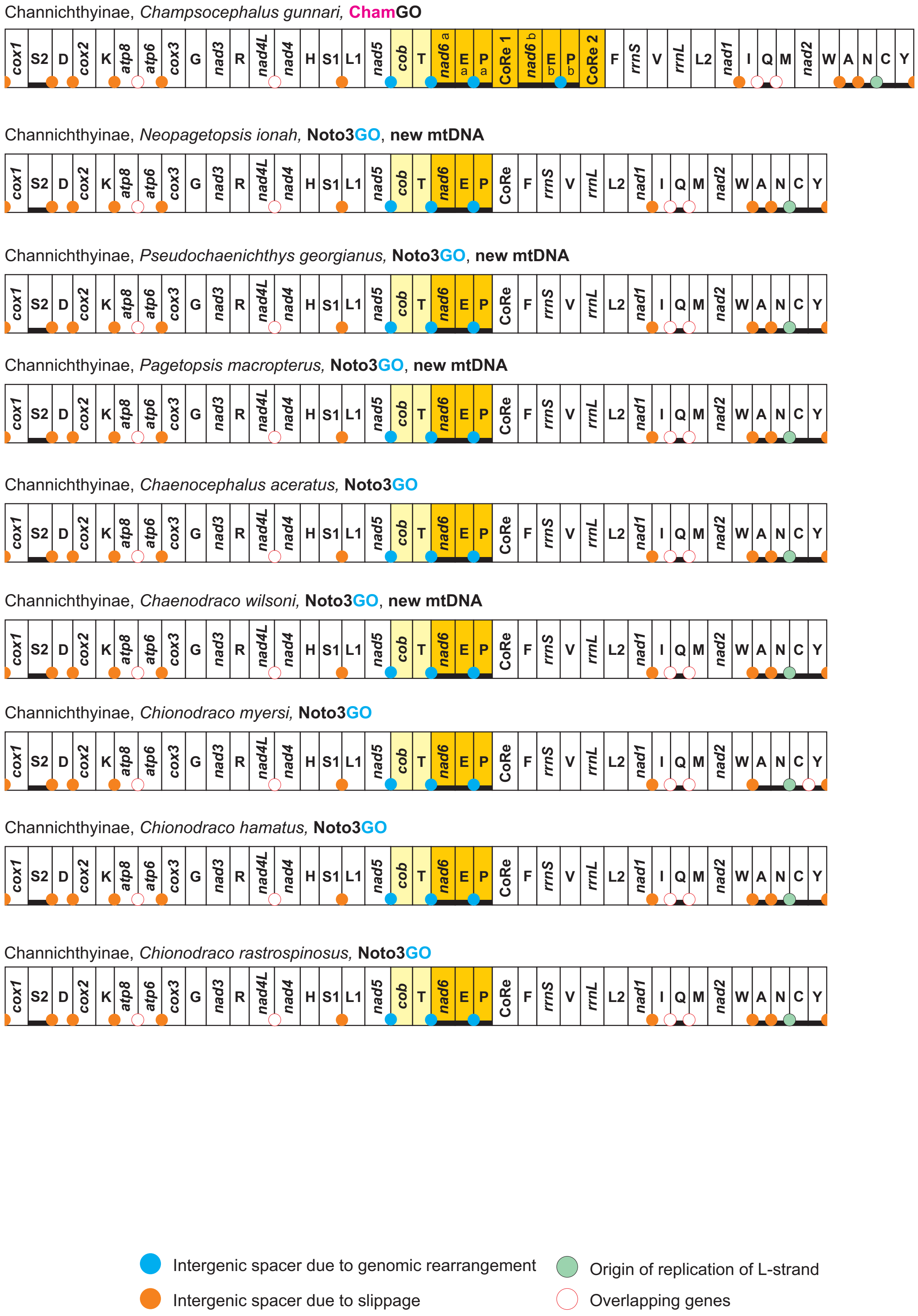

Supplementary Figure S5. Gene Orders in Notothenioidei (3). Every GO is linearized starting from *cox1*. The genes encoded on the H-strand (orientation from left to right in supplementary fig. S5) are black-boxed, while those encoded on the L-strand (orientation from right to left in supplementary fig. S5) are underlined and black-boxed. Nomenclature: *atp6* and *atp8*: ATP synthase subunits 6 and 8; *cob*: apocytochrome b; *cox1-3*: cytochrome c oxidase subunits 1–3; *nad1-6* and *nad4L*: NADH dehydrogenase subunits 1–6 and 4L; *rrnS* and *rrnL*: small and large subunit ribosomal RNA (rRNA) genes; X: transfer RNA (tRNA) genes, where X is the one-letter abbreviation of the corresponding amino acid, in particular L1 (CTN codon family) L2 (TTR codon family), S1 (AGN codon family) S2 (TCN codon family); CoRe: Control Region. Genes transposed/duplicated with respect to the VertGO have a yellow (gene belonging to the 5' duplicated block) and orange (gene belonging to the 3' duplicated block) background, while genes translocated and moved on the L-strand have a light blue background.

GR-ISP ***trnE*-*trnT*$_b$** in *Aethotaxis mitopteryx*
(fig. 4a; supplementary fig. S3, supplementary Material, online)

Pairwise alignment of the 3’ terminal portion of *Aethotaxis mitopteryx* **cob** with the intergenic spacer *Aethotaxis_mitopteryx* ISP ***trnE*-*trnT*$_b$**

```
                                              1090      1100      1110      1120      1130      1140
                                                 |         |         |         |         |         |
Aethotaxis mitopteryx cob             ATTTTCCTCATCCTCTTCCCACTCGCAGGCTGACTAGAAAACAAAGTCCTAGGCTTGGTAT
Aethotaxis_mitopteryx ISP trnE-trnTb  --CCTGGTGGCGAGCGCCATAACC-CTGGCTGACTAGAAAACAAAGTCCTAGGCTTGGTAT
```

N, identical nucleotide/amino acid in an identical DNA segment/polypeptides

Aethotaxis mitopteryx ISP-***trnE*-*trnTb*** (the frame 1 encodes a peptide)
CCTGGTGGCGAGCGCCATAACCCTGGCTGACTAGAAAACAAAGTCCTAGGCTTGGTAT

Aethotaxis mitopteryx ISP ***trnE*-*trnTb*** (peptide encoded by the ISP_**trnE**-**trnTb**)
PGGERHNPGWLENKVLGLV

Aethotaxis mitopteryx **CYTB**
MASLRKTHPLMKIANDALVDLPAPSNISVWWNFGSLLGLCLIAQILTGLFLAMHYTADINTAFSSVAHITRDVNYGWLIRDMHANGASFFFICIYMHVGRGLYYGSYLYKETWNVGVILLLLVMMTAFVGYVL
PWGQMSFWGATVITNLLSAVPYMGNALVQWIWGGFSVDNATLTRFFAFHFLFPFVVAGATMVHLLFLHQTGSNNPLGLNAGGDKIPFHPYFSYKDLLGFAALLVALATIALFTPNLLGDPDNFTPANPLVTPP
HIKPEWYFLFAYAILRSIPDKLGGVLALLASILVLLVVPFLHTCKLRSLTFRPFSQFLFWALIANVAVLTWIGGMPVEDPYIIVGQAASVLYFSIFLILFPLAGWLENKVLGLV

GR-ISP ***trnE*-*trnT*$_b$** in *Pleuragramma antarctica*
(fig. 4a; supplementary fig. S3, supplementary Material, online)

Pairwise alignment of GR-ISP ISP ***trnE*-*trnTb*** with the 3’ terminal portion of *Pleuragramma antarctica* **cob**

```
                                                        1080      1090      1100      1110      1120      1130      1140
                                                           |         |         |         |         |         |         |
Pleuragramma antarctica JF933905 cob               GTACTTCTCTATTTCCTCCTCTTCTTCCCCCTGGCGGGATGACTGGAAAATAAGGTCCTGGATTTGGCAT
Pleuragramma antarctica JF933905 ISP trnE-trnTb    ------CCTTGTAAAAAGCCTCTTCTTCCCCCTGGCGGGATGACTGGAAAATAAGGTCCTGGATTTGGCAT
```

>Pleuragramma_antarctica_JF933905_ISP_***trnE*_*trnT*$_b$** (the frame 2 encodes for a **peptide**)
CCTTGTAAAAAGCCTCTTCTTCCCCCTGGCGGGATGACTGGAAAATAAGGTCCTGGATTTGGCAT

Pleuragramma Antarctica JF933905 ISP ***trnE*-trnT$_b$** (**encoded peptide**)
LVKSLFFPLAGWLENKVLDLA

>Pleuragramma_antarctica_JF933905_**CYTB**
MASLRKTHPLLKIANDALVDLPAPSNISVWWNFGSLLGLCLITQILTGLFLAMHYTADINTAFSSVAHITRDVNYGWLIRDMHANGASFFFICIYMHIGRGLYYGSYLYKETWTVGVILLLLVMVTAFVGYVL
PWGQMSFWGATVITNLLSAVPYVGGTLVQWIWGGFSVDNATLTRFFAFHFLFPFVIAGATMVHLLFLHQTGSNNPLGLNSTGDKIPFHPYFSYKDLLGFATLLVALASIALFTPQLLGDPDNFTPANPLVTPP
HIKPEWYFLFAYAILRSIPDKLGGVLALLASILILLVVPYLHTCKLRSLTFRPLSQFLFWSLVANVAILTWIGGMPVEDPYIMIGQIASVLYFSIFLLFFPLAGWLENKVLDLA

Supplementary Figure S6

Alignments of genes involved in genomic rearrangements and the associated intergenic spacers (GR-ISP). The alignments were performed with the ClustalW program, available at the PRABI/Rhone-Alpes Bioinformatics Center (https://npsaprabi.ibcp.fr/cgi-bin/npsa_automat.pl?page=/NPSA/npsa_server.html). Successively, the alignments were manually improved through visual inspection. rc_sequence_name = reverse complement sequence of a gene encoded on the L-strand.

GR-ISP ***trnP-nad6*** in *Lindbergichthys nudifrons*
(fig. 4a; supplementary fig. S3, Supplementary Material online)

Lindbergichthys nudifrons **CoRe**

```
AAAGTGGGGCCTAAATAGCAGACGCCCCACTTACATTTATGTACTTTTAAGTACATCTATGTACTTATAGCATGCGTTTATATTAAACATTTGACGGGCATTCCTACACAATATTTGTTTGAATAACAAAAAT
TGTTTTAACACATACGTTTATATTAAACATTTGACAGGCATTCCTGCACAATATTTGTTTGAATAACAAAAATTGTTTTAACACATACCATGTACCAAATCTCACGAATAAATGTACATTAGACACCTTGAGA
AATATTCCCCTTCCCCATGTATTAACAGGGGCGAAATTTAATGTAGTACTTATATACCCTTCAATAAAGTTACACGTTTACTCCACTTCCAGGTTAAATCACAAATTCTTAATGTAGTAAGAGCCTACCAACA
AGATCATAGCTTAAGGCTAACGGTTAATGATGGTGAGGTACAAATATAGTAGGGGTTACATATAGTGAATTATTCCTGGCATTAGTTCCTATTTCAGGGCCATTAACTGAAACCACTCCTCATAACTTTACCG
ACGCTTACAAATCTCATGTTGGTGATACATACTCCTCGTTACCCAGCAAGCCGAGCGTTCACTCCAGGGTGTAGGGGGGTTTCCTTTTTTTTTTTTTCCCTTCACTAACATATCAGAGTGCACGCGGGCTTAAA
AACCAAGAGTGCACATCCTTTAGTGTATCGTTGAAACAGTATGAACTATAGTAAGACTCATTATTATGATTTTCATTTTTCTTTTTCACGGGCATAAGGAAACAAGAATTTTCCTCGTAAAAACTTCTGTTAT
GGGAGTTTTTCTAGATTCTTCATCGTTAGATCCCCCTACCCCCTTACTTCAGTAAGGGGTCTAAGACCTTAAGGACGAAGAAAGATTCAGATTACCATCTGGCGCTTGCTTTTAACAAGACGAACAAACATGT
TTTAACATTTACAATGTATTTTATTAAAACTAATACCCCCCCCCCAAAAACTTCTTCAAGTAATTTTAAACAGTATACACTGTAACCAAGCCACAACTCTCCACCCCGTTTTTTCTTCAATAAGAAAAATCAG
ACTTGGGGGGGGGGGCAGACTTATAGTTGTCATCTGGAGCTTAATTTTTAATAAAATGCATCCACATGTTTTAGCATTTATAATGTACTTCATTAGCTCTGTTCGAACTAATGGCCCCCAAAAACTTCAAGTA
ATTTTAAACAGTATACACTGTAACCAAGCCACAACTCTCCCTCCCCATTTCTTCTTCAAAAAGAGAGAGCAGACTTATAGTTATCATATGGAGCTTGCTTTCTAACAAGATGAACGAACATGTTTTAATATAT
ACAATGTACTTTGTAAGCCCTATTTGAACTTACACCCCCCCCCCAAGAAACTTTTTTCAAATAATTTTGAACACTATACACTGTAGCAGTGTCCCAACTCTCTGCCCCCTTTCAATGTATTAGTAGCCTCACA
CTCATATACCAGTGGTGGTCTCAGTGAATGCGCTGGAAGCGCATGGGGGGGGGTATTTTAAACACCCCAAAAAACCCTAATTAATTACCATTTTGAGAGTTAAAAAACTACAGCCTTAAGTCACCATGACTTA
ACAAATCCCCTGAAACTTGA
```

Lindbergichthys nudifrons ISP ***trnP-nad6*** (intergenic spacer between ***trnP*** and ***nad6***)

```
ATCCATTTTTGTACTTGTAAGTACACATATGTATTATCAAGACCCATTTATATTAAGCACTTGATGGGTGTCCTGGTCTAACATCTGATTAAATGATAAAATTGTTTTTTATACAGGTTTATCAGAACTATTA
GAAGATAGACATTGAGCGTTTAAGAAAAATGTCTCAACCCCGTGTAATAGCAGGCGAAATTTAAGACTATACTTTTACACTAATAGGCAGGCCCATGTTTACTTAACTACCAAGCCCCCCCACCAACAAGACC
AGACCTTAAGGCTACCGGCTAATAGGGTAAAAAGGTATAAATACTGCAGGGGTTACACACAGTGAACTACCCGCGGCCCCCCCCAAGAGCCCCTTTCTTTCTTTTTTCTTTTTTTTTTTCCCCTCCCTGAAAGC
TTATGTTTGTGGTGTATACTCGTCGATACACATGCTGTTGGGTGTGGGGGGGGTAGTACAAGTTACAATAAGATTTGTCAGTTTACCGCTTTTATTCCTTCTTATGAAGCTTAGGGGTAATAAACCCCTATCT
AAATGACATAAATAAGTACGTTTTGGTCCTTACGCGCCTGAGCCCTCATCTCTGC
```

Pairwise alignment of GR-ISP ***trnP-nad6*** (remnant of CoRe1, see fig. 4a of main text) with a 5' portion of *Lindbergichthys nudifrons* **CoRe**

```
                                                    90        100       110       120       130       140       150       160       170
                                                     |         |         |         |         |         |         |         |         |
Lindbergichthys nudifrons CoRe             TATTAAACATTTGACGGGCATTCCTACACAATATTTGTTTGAATAACAAAAATTGTTTTAACAC-ATACGTTTATATTAAACATTTGACA
Lindbergichthys nudifrons ISP trnP-nad6    -------------------ATCCATTTTTG-TACTTGT---AAGTACACATA-TGTATTATCAAGACCCATTTATATTAAGCACTTGATG

                                                   180       190       200       210       220       230       240       250       260
                                                     |         |         |         |         |         |         |         |         |
Lindbergichthys nudifrons CoRe             GGCATTCCTGCACAATATTTGTTTGAATAACAAAAATTGTTTTAACACATACCATGTACCAAATCTCACGAATAAATGTACATTAGACAC
Lindbergichthys nudifrons ISP trnP-nad6    GGTGTCCTGGTCTAACATCTGATTAAATGAT-AAAATTGTTTTT-TATACAG-GTTTATCAGAACT-ATTAGAAGATAGACATTGAGCGT

                                                   270       280       290       300       310       320       330       340       350
                                                     |         |         |         |         |         |         |         |         |
Lindbergichthys nudifrons CoRe             CTTGAGAAATATTCCCCTTCCCCATGTATTAACAGGGGCGAAATTTAATGTAGTACTTATATACCCTTCAATAAAGTTACACGTTTACTC
Lindbergichthys nudifrons ISP trnP-nad6    -TTAAGAAAAATGTCTCAACCCCGTGTAATAGCA--GGCGAAATTTAAGACTATACTTTTACACTAAT-AGGCAGGCC-CATGTTTACTT

                                                   360       370       380       390       400       410       420       430       440
                                                     |         |         |         |         |         |         |         |         |
Lindbergichthys nudifrons CoRe             CACTTCCAGGTTAAATCACAAATTCTTAATGTAGTAAGAGCCTACCAACAAGATCATAGCTTAAGGCTAACGGTTAAT-G-ATGGTGAGG
Lindbergichthys nudifrons ISP trnP-nad6    AACTACCAAGC-----CCC---------------------CCCACCAACAAGACCAGACCTTAAGGCTACCGGCTAATAGGGTAAAAAGG

                                                   450       460       470       480       490       500       510       520       530
                                                     |         |         |         |         |         |         |         |         |
Lindbergichthys nudifrons CoRe             TACAAATATAGTAGGGGTTACATATAGTGAATTATTCCTGGCATTAGTTCCTATTTCAGGGCCATTAACTGAAACCACTCCTCATAACTT
Lindbergichthys nudifrons ISP trnP-nad6    TATAAATACTGCAGGGGTTACACACAGTGAACTACCCGCGGCCCC---CCCCA----AGAGCCCCTTTCTT---TCTTTTTTCTTTTTTT

                                                   540       550       560       570       580       590       600       610       620
                                                     |         |         |         |         |         |         |         |         |
Lindbergichthys nudifrons CoRe             TACCGACGCTTACAAATCTCATGTTGGTGATACATACTCCTCGTTACCCAGCAAGCCGAGCGTTCACTCCAGGGTGTAGGGGGTTTCCTT
Lindbergichthys nudifrons ISP trnP-nad6    TTTCCCCTCCCTGAAAGCTTATGTTTGTGGTGTATACTCGTCGATACACA---TGCTG-----TT------GGGTGTGGGGGGGGTAGTA

                                                   630       640       650       660       670       680       690       700       710
                                                     |         |         |         |         |         |         |         |         |
Lindbergichthys nudifrons CoRe             TTTTTTTTTTTCCC-TTCACTAACATATCAGAGTGCACGCGGGCTTA-AAAACC-AAGAGTGCACATCCTTTAGTGTATCGTTGAAACAG
Lindbergichthys nudifrons ISP trnP-nad6    CAAGTTACAATAAGATTTGTCAGTTTACC-GCTTTTATTCCTTCTTATGAAGCTTAGGGTA-ATAAACCCC--T--ATC-T--AAATGA

                                                   720       730       740       750       760       770       780       790       800
                                                     |         |         |         |         |         |         |         |         |
Lindbergichthys nudifrons CoRe             TATGAACTATAGTAAGACTCA-TTATTATGATTTTCATTTTTCTTTTTCACGGGCATAAGGAAACAAGAATTTTCCTCGTAAAAACTTCT
Lindbergichthys nudifrons ISP trnP-nad6    CATAAA-TA-AGTACGTTTTGGTCCTTACGCGCCTGAGCCCTCATCTCTGC---------------------------------------
```

Supplementary Figure S7

Alignments of genes involved in genomic rearrangements and the associated intergenic spacers (GR-ISP). The alignments were performed with the ClustalW program, available at the PRABI/Rhone-Alpes Bioinformatics Center (https://npsaprabi.ibcp.fr/cgi-bin/npsa_automat.pl?page=/NPSA/npsa_server.html). Successively, the alignments were manually improved through visual inspection. rc_sequence_name = reverse complement sequence of a gene encoded on the L-strand.

GR-ISP ***trnP-nad6*** in *Trematomus borchgrevinki*
(fig. 4a; supplementary fig. S3, Supplementary Material online)

Trematomus borchgrevinki **CoRe**

GGCAGTCTACTTTTAGACTTTTCAAGTCTGCCGAGCTTACCCACACGGATAATTCAACGCAACCTGGCCCCGAACCCACGCCCTTAATGTACATACAAGTACACCTATGTACGTGACATAGTTTATATTAAAC
ATTTATATGGGTATTAAGGGTCAATATGTCTATGTATTATCACCATAGATTTATATTAAACACTTCATGGACATTCAGGGTCAATACATGGCGAAAATAGCAAAAGTTGATTTAATACATACAAGCATCAAAA
TCAATAGAAGGTGAACATTAAGCAATAAGTAACTATCAACAGATCCTAAATCTGGAGAAAAAAGAAACTTAAGGTGACGCTATTGGGCCATATAACAAGTTACACTTCTGCTCAACTTTCAATTATTTACAAA
TTCTTAATGTAGTAAGAACCGACCAACAAGATCATAGCTTAATGCTAACGGTTAATGAAGGTGAGGTACAAATATAGTAGGGGTAACAAACAGTGAATTATTCCTGACATTTGGTTCCTATTTCACGGCCATT
AATTGGTGGCATTCCTCCAAACTTAACCGACACTTACAAATCTCATGTTTGTAATACATACTCCTCGTTACCCAGCAAGCCGGGCGTTCACTCCAGCGAGCCAGGGGTTCCTTTTTTTTTTTTTCCCTTCATT
TTGCATAACAGAGTGCACACGGGCTTAACAGACAAGCGTGAGCACTTTCCTTGCTTGAACGTAAAGTAGTATGAATTATAGTAAGACCCTTTACCTATAACTTTCATTTTATTTTTTCACGGGCATAAGGGGT
CAACACTCTCTCGAGAGAGCTCCATAAGGAGTTTATAATAGGTATCTATCGGTAGATTCCCCTACCCCCTTAATTACCAGACATATCTAACGCTTTTAAGACTCAACAAGAGCAACAAGTTTGAAAAACCAGA
CAGGCCAAACAGGACAGACAGGCCTCGAAAAAGAGCATGATCTAAACAGGACAAATTACCCTTAACAAGACAAGCGAGACAGACAGGCCAAACAGGGCTGTTCTAGAACATTGCGGAGTTCTTTTAAATATTA
TAGTGTCATAAAACAAAAACTTATTTTATCAAGTATTTTTAAATATTATATAGTGTCATAAGACAAAAACTTATTTTATCAAGTATTTTTAAATATTACATAGTGTCATAAAACAAAAACTTATTTTATCAAG
TATTTTTAAATATTACATAGTGTCATAAAACATAAACTTCTTTTATGAAGTGCTTTTAAATATTACACAGCCTAGCTAGGTCCCAAAATTCTTTTATGAAGTACTTTTAAACGTTACACAATGTCATAAAACA
TAAACTTCTTTTATGAAGTGCTTTTAGATATTACACAGCCTAGCCAGGTCCCAAAATTCTTTTATGAAGTACTTTTAAACGTTATACAGTGTGATAAAAACACTATACAGTGTAGTGAAGCCCTGACCCCATT
TTTTAGGAAACTATTTTTTTTGATGCACACACCTTGTTATAGTCTCAGTGAGGGCAGCTCATTCCCCCCCCCCAGATCACTAATGGTTTTTAACCCGTCTATTCGGGCTAAATGCCTCTCATAATGTACCATGC
TCACGCAGTTGAGCAAGCGGGAATTAATTGCGGACTCACAATTAGCATTTATTTTATACGCCCGGTAATAACCGTTTTTGAAAGTAGCCCTACTGTTAACCTAG

Trematomus_borchgrevinki ISP ***trnP-nad6*** (intergenic spacer between ***trnP*** and ***nad6***)

GCCCCCTTATATACATAATCATATACTTGTATAAATCATCTCATGGGTAATACATAATGAAAGTGGTAAAAATTGATCCTATTCACACACGTGTGTCAAAATTAATAAAAGGTAAACATCACCGGGCCCTAAA
TCTGGCGAAAAAGAAACTTAGGGTGACGCTATTGACGTGTTTAACAAGTTACACTTTTACATATACTCTCCTTCCCCCGTTGCCCAGCAAGCTGGGCGGGGGGGGGGGTGAGTTATAATAAGACTTGATACTT
TTTTATCCGGGGGGGTCAAGGGGATGTAAACTACCCTTTAAATAAGATAAAAGAGATTACACGGATCCCCAAGCGCCTGAGCCCAACCTTCACCCGTTTGTGGGGGGGGGGCC

Pairwise alignment of GR-ISP ***trnP-nad6*** (remnant of CoRe1, see fig. 4a of main text) with a 5' portion of *Trematomus borchgrevinki* **CoRe**

```
                                                   160       170       180       190       200       210       220       230
                                                     |         |         |         |         |         |         |         |
Trematomus borchgrevinki CoRe                GGGTCAATATGTCTATGTATTATCACCATAGATTTATATTAAACACTTCATGGACATTCAGGGTCAATACATGGCGAAAA
Trematomus borchgrevinki ISP trnP-nad6       ------GCCCCCTTATATAC-ATAATCATATACTTGTATAAATCATCTCATGG---------GT-AATACATAATGAAAG

                                                   240       250       260       270       280       290       300       310
                                                     |         |         |         |         |         |         |         |
Trematomus borchgrevinki CoRe                TAGCAAAAGTTGATTTAAT--ACATACAAGCATCAAAATCAATAGAAGGTGAACATTAAGCAATAAGTAACTATCAACAG
Trematomus borchgrevinki ISP trnP-nad6       TGGTAAAAATTGATCCTATTCACACACGTGTGTCAAAATTAATAAAAGGTAAACAT------------------CACCGG

                                                   320       330       340       350       360       370       380       390
                                                     |         |         |         |         |         |         |         |
Trematomus borchgrevinki CoRe                ATCCTAAATCTGGAGAAAAAAGAAACTTAAGGTGACGCTATTGGGCCATATAACAAGTTACACTTCTGCTCA-ACTTTCA
Trematomus borchgrevinki ISP trnP-nad6       GCCCTAAATCTGGCGAAAAA-GAAACTTAGGGTGACGCTATTGACGTGTTTAACAAGTTACACTTTTACATATACTCTC-

                                                   400       410       420       430       440       450       460       470
                                                     |         |         |         |         |         |         |         |
Trematomus borchgrevinki CoRe                ATTATTTACAAATTCTTAATGTAGTAAGAACCGACCAACAAGATCATAGCTTAATGCTAACGGTTAATGAAGGTGAGGTA
Trematomus borchgrevinki ISP trnP-nad6       ----CTT-CC--CCCGTTGCCCAGCAAGC-TGGGCGGGGGGGGGGGTGAGTTA-TAATAA-GACT--TGATACT----TT

                                                   480       490       500       510       520       530       540       550
                                                     |         |         |         |         |         |         |         |
Trematomus borchgrevinki CoRe                CAAATATAGTAGGGGTAACAAACAGTGAATTATTCCTGACATTTGGTTCCTATTTCACGGCCATTAATTGGTGGCATTCC
Trematomus borchgrevinki ISP trnP-nad6       TTTATCCGGGGGGGTCAAGGGGATGTAAACTACCCTTTAAATAAGA----TA----AAAGAGATTACAC---GG-ATCCC

                                                   560       570       580       590       600       610
                                                     |         |         |         |         |         |
Trematomus borchgrevinki CoRe                TCCAAACTTAACCGACACTTACAAATCTCAT--GTTTGTAATACATACTCCTCGTTACCC
Trematomus borchgrevinki ISP trnP-nad6       --CAAGC--GCCTGA-GCC--CAACCTTCACCCGTTTGTGGGGGGGGGGCC---------
```

Supplementary Figure S8

Alignments of genes involved in genomic rearrangements and the associated intergenic spacers (GR-ISP). The alignments were performed with the ClustalW program, available at the PRABI/Rhone-Alpes Bioinformatics Center (https://npsaprabi.ibcp.fr/cgi-bin/npsa_automat.pl?page=/NPSA/npsa_server.html). Successively, the alignments were manually improved through visual inspection. rc_sequence_name = reverse complement sequence of a gene encoded on the L-strand.

GR-ISP ***trnP-nad6*** in *Trematomus eulepidotus*
(fig. 4a; supplementary fig. S3, Supplementary Material online)

Trematomus eulepidotus **CoRe**

```
GGGGGGGGGCGGGAACTTAAGGCCACTTGCCTTTTCAAAGCTGCCGAGCTTACCCACACGCCAGAAAATGGCGCGGGGTGGTGACTCAACGCAGCCTGAGCCCGCCCTAATGTACCCACAAGTACAATTTTAC
ACAAATATGTCTATGTATTATCACCATAAATTTATATTAAACACTTCATGGATATTCAGGGTCAATACATGACGAAAATAGCAAAAGTTGATTTTACACAAATATGTCTATGTATTATCACCATAAATTTATA
TTAAACACTTCATGGATATTCAGGGTCAATACATGACGAAAATAGCAAAAGTTGATTTTACACAAACAAACATCACAACTAATAAAAGGTACACATTAAGCATTTAGTAGACTTCACCAGATCCTAAATATGA
GGACAAAAGAAACTTAAGGTAGTACTAGAAAATCATTTAACAAGTTACACTTTTACTCAACTTTCAGTTATCCTCAAATTCTTAATGTAGTAAGAACCGACCAACAAGATCATAGCTTAATGCTAACGGTTAA
TGAGGGTCAGGTACAAATATAGTAGGGGTTTCACATAGTGAATTATTCCTGGCATTTGGTTCCTATTTCAGGGCCATTAATTGGTGGCACTCCTCCAAACTTAACCGACACTTACAAATCTCATGTTTGTAAT
ACATACTCTTATGTTACCCAGCAAGCCGGGCGTTCACTCCAGCGAGCAAGGGGTTCCTTTTTTTTTTTTCCCTTCATTTTGCATATCAGAGTGCACACGGGCTTAACAAACAAGCGAGAGCACTTTCCTTGCC
TAATATAAATAGTATGAACTATAGTAAGACCCTGAACCTATAACTTTCATTTTTTTTTTTTCACGGGCATAAGGGGTCAAAATTCTTTCGGAAGAACTCCATAAGGGAGTTATAACAGGTGTTCATCGGTAGAT
TCCCCCTACCCCCTTACTTACGAGACATGTCTAAGGCTTTCAAGAGGGGCCAAACCTCAACAAGACAAGTTTATTTCAACAAGACAGACAGACTAAATAGGACAAACGAGCCTCAATAAAATCCACGGGCCAA
ACAGGACAAATAAACCTCAACAAGATGAACAAGATGAACAAGCCTTTAAAAACACTATACAGTGTAATAAAAAACACTATACAGTGTAATAAAAACACTATAAACAGTGTAATAAAAACACTATACAGTGTAA
TAAAAACACAAACTTCTTTTAAACACTATATAGTGTAATAAAAACACAAACTTCTTTTAAACACTATACAGTGTGGTGAGGCCCGCTACCGGGCAGCTCACTTCCCCCAAAGCATGAAAATTTTTATGGCGGC
TATCCGAGTTATATACATCTTACACAATTCAACAAGCGGGGCCCCCACTCAATTAATTGTGGACTCACAATTAACAGTTATTTTACACACCTAACAACAACCTAAAGTCTTCTAAAACTAG
```

Trematomus eulepidotus ISP ***trnP-nad6*** (intergenic spacer between ***trnP*** and ***nad6***)

```
ACCCCTTTGTGTTCAAAACCATAAATTTATATTTAAACTTTGGGGGGCACAGATATAGTAGGGGCCCCCAAAGTGAATTACTCCTGACATTTGGTTTTTATCTCAGGGCCATTAATTGGTGCCACCCCCCCC
CCCAAAAAAAAATAATATAAGAGAAGTTATACGGATTCCCAAGCGCCTGAGCCCCACCTTCACCCGTTTGGGGGCC
```

Pairwise alignment of GR-ISP ***trnP-nad6*** (remnant of CoRe1, see fig. 4a of main text) with a portion of *Trematomus eulepidotus* **CoRe**

```
                                            480       490       500       510       520       530       540       550       560
                                              |         |         |         |         |         |         |         |         |
Trematomus eulepidotus CoRe           CCTCAAATTCTTAATGTAGTAAGAACCGACCAACAAGATCATAGCTTAATGCTAACGGTTAATGAGGGTCAGGTACAAATATAGTAGGGG
Trematomus_eulepidotus ISP trnP-nad6  -----ACCCCTT--TGTGTTCAAAACC-----ATAAATTTATATTTAA-----AC--TT--TGGGG----GGCACAGATATAGTAGGGG

                                            570       580       590       600       610       620       630       640       650
                                              |         |         |         |         |         |         |         |         |
Trematomus eulepidotus CoRe           TTTCACATAGTGAATTATTCCTGGCATTTGGTTCCTATTTCAGGGCCATTAATTGGTGGCACTCCTCCAAACTTAACCGACACTTACAAA
Trematomus_eulepidotus ISP trnP-nad6  CCCC-CAAAGTGAATTACTCCTGACATTTGGTTTTTATCTCAGGGCCATTAATTGGTGCCACCCCCCCC--CCCAAAAAA-AAT----AA

                                            660       670       680       690       700       710       720
                                              |         |         |         |         |         |         |
Trematomus eulepidotus CoRe           TCTCATGTTTGTAATACATACTCTTATGTTACCCAGCAAGCCGGGCGTTCACTCCAGCGAGCAAGGGGTT
Trematomus_eulepidotus ISP trnP-nad6  TATAAGAGAAGTTATACGGATTCCCAAGC-GCCT---GAGCCCCACCTTCACCC--GT---TTGGGGGCC
```

GR-ISP ***trnP-nad6*** in *Trematomus tokarevi*
(fig. 4a; supplementary fig. S3; Supplementary Material online)

Trematomus tokarevi **CoRe**

```
GGTTGGAGGACTTTGGGCCACCAACCTTTTCAAAGCTGCCGAGCTTATCCACACGCCATTTTTATGCAGTGACATAATGGGGGCAGACGAACTAAATCGATAACAAAGCCCTTTACCCCGCACTACACCCTGA
ATGTACTTACAAGCACGTCTATGTATTATCACCATAAATTTATATTAAACACTTCATGGATATTCACGGGCAATATATGACAAAAATAGCAAAAGTTGATTTTACACAAATATGTCTATGTATTATCACCATA
AATTTATATTAAACACTTCATGGATATTCACGGGCAATATATGACAAAAATAGCAAAAGTTGATTTTACACAAACAAATATCAAAACTAATAAAAGATATACATTAAGCATTTAGTAGACATCACCCGATCCT
AAATATGAGGACAAAAGAAACTTAAGATAACACTATTGGGCCATATAACAAGTTACACTTTTATTCAACTTTCAGTTATTCACAAATTCTTAATGTAGTAAGAACCGACCAACAAGATCATAGCTTAATGCTA
ACGGTTAATGAGGGTGAGGTACAAATATCGTAGGGGTTTCACTCAGTGAATTATTCCTGGCATTTGGTTCCTATTTCAGGGCCATTGATTGGTAACAGTCCTCCAAACTTAACCGACACTTACAAATCTCATG
TTTGTAATACATACTCCTCGTTACCCAGCAAGCCGGGCGTTCACTCCAGCGAGCCAGGGGTTCCTTTTTTTTTTTTCCCTTCATATTGCATATCAGAGTGCACACGGGCTTAACAAACAAGCGTGAGCACTTT
CCTTGCCTAAATATAAATAGTATGAGTTATAGTAAGACCCCTACCTATAACTTTCATTTTATTTTTTCACGGGCATAAGGGGTCAAAACTCTCTCGGAGAAACTCCATAAGGGAGTTATAATAGATCTTCATC
GGTAGATTCCCCCTACCCCCTTACTTACGAGACATGTCTAAGGCTTTCAAGAGGGGTCAAACCTCAACAAGACAAGTTTATCTCGACAAGACAAACAGACCGATCGGGACAAAAAAACTTTTACAAAACACAT
GGACCAGGCGGGACAAAAAACTTTTACAAAACACATGGACCAGCCGGGACAAAAAAGCCTTAACAAGACTAATCTAGCACATTACGAAGTACTTTTATATATTATACAGTGTTATAAAAACACAAACTTATTT
CATCGAAGTACTTTTATACACTAAAAGGGGTTATAAAAACACAAACTTCTTTTACGAAGTACTTTTAAACACTATACAATGTAGTGAAAACACAAACTTCTTTTACGAAGTACTTTTAAACACTATACAATGT
AGTGAAAACACAAACTTCTTTTACGAAGTACTTTTAATAACAACTATTCGGGTTATGTACATCTCACATTGTACTATACCCACGCAGTGGGAAAGCGGGGATCAAGGTAAACTAATTGTGGACTCACAATTAG
CAATTATTTTATACACATGAAAACAACCTTAAGTCTCTTAAAACTAG
```

Trematomus tokarevi ISP ***trnP-nad6*** (intergenic spacer between ***trnP*** and ***nad6***)

```
ACCCCCCCCCCATGTTCAAAACCATAAATTTATATTAAACACTTCATGGATATTCACGGGCAATACATGACGAAAATAGCAAAAGTTGATTTTATATAAACATATGTCAAAACTAATAAAAGATAGACATTAA
GCATTTAATATAAATTTCCACACAGATAATATAAAAGAAGTTATATGGACCCCCCCAAGCGCCTGAGCCCCGCCTTCACCCGTCGGGGGCC
```

Pairwise alignment of GR-ISP ***trnP-nad6*** (remnant of CoRe1, see fig. 4a of main text) with a portion of *Trematomus tokarevi* **CoRe**

```
                                          240       250       260       270       280       290       300       310       320
                                            |         |         |         |         |         |         |         |         |
Trematomus tokarevi CoRe            GATTTTACACAAATATGTCTATGTATTATCACCATAAATTTATATTAAACACTTCATGGATATTCACGGGCAATATATGACAAAAATAGC
Trematomus tokarevi ISP trnP-nad6   ------ACCC---CCCCCCATGT-TCAAAACCATAAATTTATATTAAACACTTCATGGATATTCACGGGCAATACATGACGAAAATAGC

                                          330       340       350       360       370       380       390       400       410
                                            |         |         |         |         |         |         |         |         |
Trematomus tokarevi CoRe            AAAAGTTGATTTTACACAAACAAATATCAAAACTAATAAAAGATATACATTAAGCATTTAGTAGACATCACCCGATCCTAAATATGAGGA
Trematomus tokarevi ISP trnP-nad6   AAAAGTTGATTTTATATAAACATATGTCAAAACTAATAAAAGATAGACATTAAGCATTTAATATAAATT-TCCA--CACAGATA---ATA

                                          420       430       440       450       460       470       480
                                            |         |         |         |         |         |         |
Trematomus tokarevi CoRe            CAAAAGAAACTTAAGATAACACTATTGGGCCATATAACAAGTTACACTTTATTCAACTTTCAGTTATTC
Trematomus tokarevi ISP trnP-nad6   TAAAAGAAG-TTATATGGACCCCCCAAGC-GCCTG---AGCCCGCCTTCACCCG----TCGGGGCC-
```

Supplementary Figure S9

Alignments of genes involved in genomic rearrangements and the associated intergenic spacers (GR-ISP). The alignments were performed with the ClustalW program, available at the PRABI/Rhone-Alpes Bioinformatics Center (https://npsaprabi.ibcp.fr/cgi-bin/npsa_automat.pl?page=/NPSA/npsa_server.html). Successively, the alignments were manually improved through visual inspection. rc_sequence_name = reverse complement sequence of a gene encoded on the L-strand.

GR-ISP *trnP*-*nad6* in *Dissostichus eleginoides* (pseudo-trnE (ψtrnE) + pseudo-CoRe2 (ψCoRe2))

(fig. 4b; supplementary fig. S3, Supplementary Material online)

Dissostichus eleginoides AB723627 ISP ***nad6-trnE*** (intergenic spacer between ***nad6*** and ***trnE***)

```
AACTCTTGCCAGGACTTTAACCGGGATTGGTGGCCTAAAAAGCCATCGTTGTTGAACTACAGGAACCCTGGAAGCAATTGCCTCCAGCCGACCCCGCTTCTAGTGTACTTGTAAGTACATTCCTCTTGTTACT
AATATTCATATAAATTAAACACCTCAATGGGCAATCCTGGACAAGGGTGATTTTATGACAGGATTTGACTTGACACACAAACCTAACAGTAAGTACACACAAAACATTTGAGAAGCCTTACATAACCCAGATT
AATGACAGGCGAGACTTAAGACCAGAACTAAATATTAATTCTTTAACAAACAGAGCAAATACTTTTGCGTTCGGGGGCCTCCCCGGAGCGATCTGGTCTATACATGCGTTAATTTCGGCACACCCTCGAACAC
TCCCGGTTTCACATGGTGAACTATTCCTGACATTTGGCTCCTACTTCAGGGCCATTGATTGGTGTCATCCCCCCCCTGACGTGTCTATGACCTACATACAACTCACAAGAACAAGGGGGCCCGTCTAACACAT
ACTTATCGGCAAGACCAGAACTAAATATTAATTCTTTAACAAACAGAGCAAATACTTTTGCGTTCGGGGGCCTCCCCGGAGCGATCTGGTCTATACATGCGTTAATTTCGGCACACCCTCGAACACTCCCGTA
ACCCCCATTTTATACCCCCTGTTGCTCGTGTCTTATCTAGGACTGCAAATTCCATAATTCTCAGCTCGAAAATAATTACTGAGCTGGAACCCCCCCCCCACAAACATGATAAACCCACACCCCATTGCTGCCA
TCACAGCCAACCCAAGGCCGCATAAAGGGGCGAGGGATTAGCGGCAACCGCCGCTATCCCCACTACTACACCCAGCATAAGTAGGTACCCGCAATATAACATA
```

Dissostichus eleginoides AB723627 **ψ*trnE*** (**pseudo-trnE, TTT wrong anticodon** (***trnK***))

```
GTTCCTGTAGTTCAACAACGATGGCTTTTTAGGCCACCAATCCCGGTTAAAGTCCTGGCAAGAGTT
AACTCTTGCCAGGACTTTAACCGGGATTGGTGGCCTAAAAAGCCATCGTTGTTGAACTACAGGAAC
```

Dissostichus eleginoides AB723627 **ψCoRe2** (**pseudo-CoRe2**)

```
CCTGGAAGCAATTGCCTCCAGCCGACCCCGCTTCTAGTGTACTTGTAAGTACATTCCTCTTGTTACTAATATTCATATAAATTAAACACCTCAATGGGCAATCCTGGACAAGGGTGATTTTATGACAGGATTT
GACTTGACACACAAACCTAACAGTAAGTACACACAAAACATTTGAGAAGCCTTACATAACCCAGATTAATGACAGGCGAGACTTAAGACCAGAACTAAATATTAATTCTTTAACAAACAGAGCAAATACTTTT
GCGTTCGGGGGCCTCCCCGGAGCGATCTGGTCTATACATGCGTTAATTTCGGCACACCCTCGAACACTCCCGGTTTCACATGGTGAACTATTCCTGACATTTGGCTCCTACTTCAGGGCCATTGATTGGTGTC
ATCCCCCCCCTGACGTGTCTATGACCTACATACAACTCACAAGAACAAGGGGGCCCGTCTAACACATACTTATCGGCAAGACCAGAACTAAATATTAATTCTTTAACAAACAGAGCAAATACTTTTGCGTTCG
GGGGCCTCCCCGGAGCGATCTGGTCTATACATGCGTTAATTTCGGCACACCCTCGAACACTCCCGTAACCCCCATTTTATACCCCCTGTTGCTCGTGTCTTATCTAGGACTGCAAATTCCATAATTCTCAGCT
CGAAAATAATTACTGAGCTGGAACCCCCCCCCCACAAACATGATAAACCCACACCCCATTGCTGCCATCACAGCCAACCCAAGGCCGCATAAAGGGGCGAGGGATTAGCGGCAACCGCCGCTATCCCCACTAC
TACACCCAGCATAAGTAGGTACCCGCAATATAACATA
```

Dissostichus eleginoides AB723627 ***trnE***

```
GTTCCTGTAGTTGAATAACAACGATGGCTTTTCATGCCGCCAGTCCCGGTTAAAGTCCTGGCAAGAGTT
AACTCTTGCCAGGACTTTAACCGGGACTGGCGGCATGAAAAGCCATCGTTGTTATTCAACTACAGGAAC
```

Dissostichus eleginoides AB723627 **CoRe2**

```
CCTGAAAGCAATTGCCTCCGGCCGGCCGCGCGCCCCCTTAATGTACTTGTAAGTGTATTCATATATATATATATTAAGCACCTCATAGGCAATCCTGGACAAGAGTGATTTTATGACAGGATTTGACTTGATA
CACAAACCTAACAGTAAGTACACACAAAACATTTGAGAAGCCTTACATAACCCAAATTAATGACAGGCGAGACTTAAGACCGAACTCACACTTCATTAGTCAAGTTATACGTTTATTTAACCTCCTGCCAATT
CACAGATTCTTAATGTGATAAGAGCCTACCAACAAGCCCGTATCTTAAGGCTAACGGTTATTGAGGGTAAGGGACAAGTATTTGTACGGGTTTCACATGGTGAACTATTCCCGGCATTTGGCTCCTACTTCAG
GGCCAATGATTGGTGTCATTCCCCCCCCTGACGTGTCTATGACCTACATACAACTCACAAGAACAAGGAGGCCCCGGAAGGGCCGTCTAACACATACTTATTAACAAAGCAAGCAAGTATATAATAACGTTTA
AAAGTACTTTTATTGACTTTATAGAGAACTATACCGCAGGTATTCACGGGCCACCCTGCTCTTAATAATACCTTGACAGTAACAGTATACAGTGACATTTAAAGGTGCTTTTATTGACTTTATAGAGAACTAT
ACCCCAGGTATTCACGGACCACCCTACTCTTAATAATACCTTACCCCCCCCACGACCTTCAGCAACTTCTCTACAATTTTTAAACACCCCTTTGTTGCCTCAACCCCCTTGAAAGCCCCCCCCAGCCTTGGCG
GGAGTTGATCTATACAGTGTTAATTTTGCGCACCTCAAGTACCTACTTCTTTCTGACCCTGCACCATTTTTTGTTGAACAAGCCTGAGCAATATTAGTTGCTTGTGGCCACCGCCCCCTGTTTGTGCCCATCT
ATACACTCTTTGATGCCCCAAGCACTGGCATTTTAGAGACCCCCAATGTACCTATGTTATTAAGACCCTCCAACCTCCTTGTTTCGGAACCTCTCGCAATTTACACGTGTATACAACTCTTGAATTACCTATA
CTCTTTAACACCTCCTTGTAGAGACTAACAACCCTGTGGATATGTCC
```

cTAS, TAS, CSB-F, CSB-E, CSB-D, CSB1, CSB2

The sequence motifs that characterize a true active control region (Zhuang and Cheng 2010)

Multiple alignment of *trnE* and ψ*trnE* of the *Dissostichus* species, based on the secondary structure predicted with tRNA-scan program.

```
                                                 10        20        30        40        50        60
                                                  |         |         |         |         |         |
                                         1234567  1234     4321 12345  ant  54321    12345       543217654321d
Dissostichus eleginoides AB723627 ψtrnE  GTTCCTGTAGTTCAA---CAACGATGGCTTTTTAGGCCACCAATCCCGGTTAAAGTCCTGGCAAGAGTT
Dissostichus eleginoides AB723627 trnE   GTTCCTGTAGTTGAATAACAACGATGGCTTTTCATGCCGCCAGTCCCGGTTAAAGTCCTGGCAAGAGTT
Dissostichus mawsoni LC138011 trnE       GTTCCTGTAGTTGAA-AACAACGATGGCTTTTCATGCCGCCAGTCCTGGTTAGAATCCTGGCAAGAATT
                                             *                  *               *      *           *    *
                                         1234567  1234     4321 12345  ant  54321    12345       543217654321d
                                         0000000  1111     1222 22222  333  33334    44444       5556666666666
                                         1234567  0123     9012 45678  123  67890    56789       7890123456789
```

Legend

N, the background base for the position.
N, half-compensatory base change in the stem pair (e.g. T – G vs C – G; A-T vs G-T).
N, a mismatch in the stem pair.
*, position in the stem characterized by a mismatch.
X, position 1-7 in the acceptor stem; **X** position 1-4 in the DHU stem; **X** position 1-5 in the anticodon stem; **X** position 1-5 in the TΨC stem; **ant**, anticodon; d, discriminator nucleotide
A compensatory base change implies the substitution of a nucleotide with a different base that does not disrupt the pairing in the stem.
(See Montelli et al. 2016)

Montelli S, Peruffo A, Patarnello T, Cozzi B, Negrisolo E. 2016. Back to water: signature of adaptive evolution in Cetacean mitochondrial tRNAs. PLoS ONE 11:e0158129.

Zhuang X, Cheng C-HC. 2010. ND6 Gene "Lost" and Found: Evolution of mitochondrial gene rearrangement in Antarctic notothenioids. Mol Biol Evol 27:1391-1403.

Supplementary Figure S10

Alignments of genes involved in genomic rearrangements and the associated intergenic spacers (GR-ISP). The alignments were performed with the ClustalW program, available at the PRABI/Rhone-Alpes Bioinformatics Center (https://npsaprabi.ibcp.fr/cgi-bin/npsa_automat.pl?page=/NPSA/npsa_server.html). Successively, the alignments were manually improved through visual inspection. rc_sequence_name = reverse complement sequence of a gene encoded on the L-strand.

Pairwise alignment of *D. eleginoides* **CoRe2** and *D. eleginoides* **ψCoRe2**

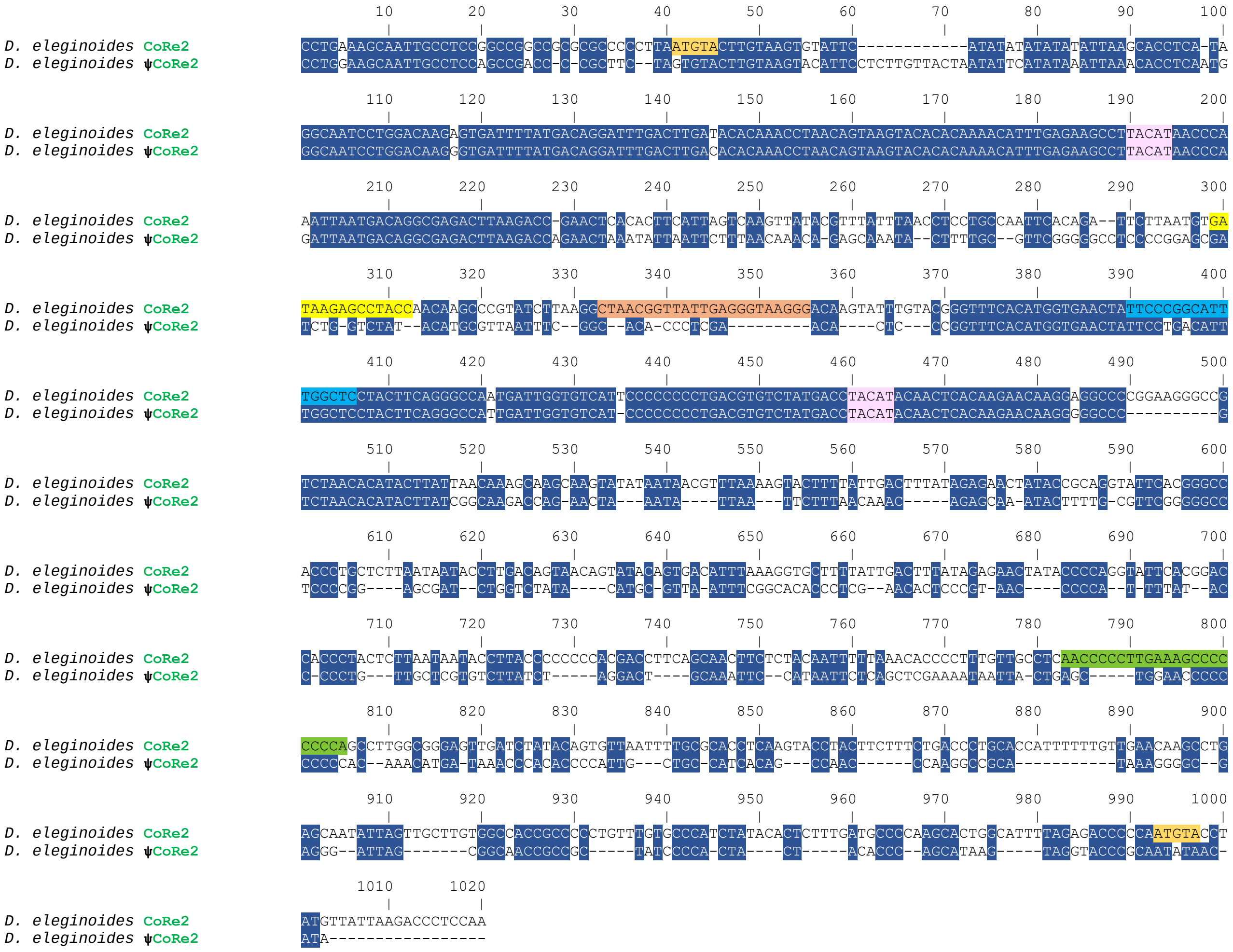

Supplementary Figure S11

Alignments of genes involved in genomic rearrangements and the associated intergenic spacers (GR-ISP). The alignments were performed with the ClustalW program, available at the PRABI/Rhone-Alpes Bioinformatics Center (https://npsaprabi.ibcp.fr/cgi-bin/npsa_automat.pl?page=/NPSA/npsa_server.html). Successively, the alignments were manually improved through visual inspection. rc_sequence_name = reverse complement sequence of a gene encoded on the L-strand.

GR-ISP *trnT*-*nad6* in *Artedidraco skottsbergi*

(fig. 5a; supplementary fig. S4, Supplementary Material online)

Pairwise alignment of GR-ISP **trnT-nad6** with a portion of *Artedidraco skottsbergi* **CoRe**

```
                                                 790       800       810       820       830       840       850       860       870
                                                   |         |         |         |         |         |         |         |         |
Artedidraco skottsbergi CoRe              ATGTCTGTCAGATTCACCAGACCCCA-AGACCAGAAGAGCCGAAGAAAGTGGGCTAACACTATGCCTCCTCACAAGAGCACCACCTGCTT
Artedidraco skottsbergi ISP trnT-nad6     --------CAAAAAGACAAAGTTTTATACCCCCCCCCCCCCAGAGTTACTAAACTCGC-CTGGGTTTTTCTAACGCCTCTACACTTCCGA

                                                 880       890       900       910       920       930       940       950       960
                                                   |         |         |         |         |         |         |         |         |
Artedidraco skottsbergi CoRe              TTCACAAGCACTTTTTTACGCCCCTTAAACACTCCCCCCCCGCGCCTGAGCCCTTATCCGCGCCTAATTCATAATACGCTCCCGCCCCTT
Artedidraco skottsbergi ISP trnT-nad6     CCTAGAGGCG-----TTACGCCCCTTAAACACTCCCCCCCCGCGCCTGAGCCCTTATCCGCGCCTAATTCATAATACGCTCCCGCC----
```

GR-ISP *trnT*-*nad6* in *Dolloidraco longedorsalis*

(fig. 5a; supplementary fig. S4, Supplementary Material online)

Pairwise alignment of GR-ISP ***trnT*-*nad6*** with a portion of *Dolloidraco longedorsalis* **CoRe**

```
                                                  1060      1070      1080      1090      1100      1110      1120
                                                     |         |         |         |         |         |         |
Dolloidraco longedorsalis CoRe              TCACAACAACTCAACAAGTATTTCACCCCCCCCCCCACCAGTTACAGCACCTAGCTTGTGTATATATAG
Dolloidraco longedorsalis ISP trnT-nad6     ------CAAAGAGACGAAATTTTATGCCCCCCCCCAGAGTTATTAAGCTGCCTGGGTTTTCTA-ACGCCT

                                                  1130      1140      1150      1160      1170      1180      1190
                                                     |         |         |         |         |         |         |
Dolloidraco longedorsalis CoRe              ATACATATTTTGATCAAAACACCCCCCCCCCCAGCGCTCTAACCCCCCCCCCCCCCCAGCCCCCACCTATT
Dolloidraco longedorsalis ISP trnT-nad6     CTACAC-TTCCGACCAAGG-------------GGCGTTACGCCCCCCCCCTCCT---------------
```

GR-ISP *trnT*-*nad6* in *Akarotaxis nudiceps*

(fig. 5a; supplementary fig. S4, Supplementary Material online)

Pairwise alignment of GR-ISP ***trnT*-*nad6*** with a portion of *Akarotaxis nudiceps* **CoRe**

```
                                          710       720       730       740       750       760       770       780       790
                                            |         |         |         |         |         |         |         |         |
Akarotaxis nudiceps CoRe           CATCGGAATCCCCCCCACCCCCTTACTCCCCTGACATACCTAACACCCACCAAATCCCACAAAGCCGGGGGGGGCCCGGGGAAAGCGGGC
Akarotaxis nudiceps ISP trnT-nad6  --T-GAAAACAGGCTCCCCCCCCCCCGCGCTTAA-A-GCCGAGCAGGGCCTGCAGCGCGGGGGTGCTAAAACTAGCCGCGGAAAGCGGGC

                                          800       810       820       830       840       850       860       870       880
                                            |         |         |         |         |         |         |         |         |
Akarotaxis nudiceps CoRe           CACACCCGTCCCGTCCAACACTTTTACTGGCCCCACAAGTGCTTCTCTCCCCCCAATCTCGGGGGTGCCCCACGCCTGCGCCTTTTTTTT
Akarotaxis nudiceps ISP trnT-nad6  CACACCCGTCCCGTCCAACACTTTTACTGGCCCCACAAGTGCTTCTCTCCCCCCAATCTCGGGGGTGCCCCACGCCTGCGCCCACTCCCG

                                          890       900       910
                                            |         |         |
Akarotaxis nudiceps CoRe           ATAGGTCAGGTGCATACCTGCCGTAAAAAC
Akarotaxis nudiceps ISP trnT-nad6  CTGCAT-AAAAACAGTCCCGCC--------
```

GR-ISP *trnT*-*nad6* in *Gerlachea australis*

(fig. 5a; supplementary fig. S4, Supplementary Material online)

Pairwise alignment of GR-ISP ***trnT*-*nad6*** with a portion of *Gerlachea australis* **CoRe**

```
                                          850       860       870       880       890       900       910       920       930
                                            |         |         |         |         |         |         |         |         |
Gerlachea australis CoRe           CGCGCACCCCCCCCCCACCCCCCACAAACACTTTTCTTTGCTTATAGGAGCTCCTATACAGTAATGCCAGCTTATATACCCCTCCTTAAC
Gerlachea australis ISP trnT-nad6  -------------CAAAACAGTTTTTAACACCCCTCCC--CTCCCCACACTTCCGACCAAAAACCTCCAACTT-T-TACCACTTTTTACC

                                          940       950       960       970       980       990      1000      1010      1020
                                            |         |         |         |         |         |         |         |         |
Gerlachea australis CoRe           CCCCCCCCCCCCATATTTACCCCAGCTAACAAGATAAACTAATATGGCCCACCCCCCCCCCAGCTAACAAGATAAACTAATATGACCCGC
Gerlachea australis ISP trnT-nad6  ACTTTCCACCATATATTTACCCCGGCTAACAAGATAAACTAATATGGCCCACCCCATCTCAGG-----AGTACCTACCCCCGCGCCTGAG

                                         1030      1040      1050      1060      1070
                                            |         |         |         |         |
Gerlachea australis CoRe           CCCCAAGCATCAGCTGCTCTCAAAA-ACACTTCTCACCAATTTCAACTGG
Gerlachea australis ISP trnT-nad6  CCCTTGCCTTCGCCCCCTTACAACACAAACCCATCCCC------------
```

Supplementary Figure S12

Alignments of genes involved in genomic rearrangements and the associated intergenic spacers (GR-ISP). The alignments were performed with the ClustalW program, available at the PRABI/Rhone-Alpes Bioinformatics Center (https://npsaprabi.ibcp.fr/cgi-bin/npsa_automat.pl?page=/NPSA/npsa_server.html). Successively, the alignments were manually improved through visual inspection. rc_sequence_name = reverse complement sequence of a gene encoded on the L-strand.

GR-ISP *trnT*-*nad6* in *Neopagetopsis ionah*
(fig. 5a; supplementary fig. S5, Supplementary Material online)

Pairwise alignment of GR-ISP *trnT*-*nad6* with a portion of *Neopagetopsis ionah* CoRe

```
                                        1470      1480      1490      1500      1510      1520      1530
Neopagetopsis ionah CoRe             TGTAACACCTAAACCCCACACACCCAGTAAAAACCCGCCACTTTTTAACTGTGTCTACTGGTTTTTAACG
Neopagetopsis ionah ISP trnT-nad6    ----ACACCTAAACCTCACAAACTCATTAAGCCAAAAGCACCTAAGAGAATCGCCTACATACGCCT----

                                        1540      1550      1560      1570      1580      1590      1600
Neopagetopsis ionah CoRe             GCCCCCCCCACTTTCTTACTCACGCACACCTTTCTTCAACATGTATCGCCCCTATACAGTGATACAAAT
Neopagetopsis ionah ISP trnT-nad6    -CCTCACAACACTTTCTCTAGCCCCCCGACGGGTGGCAACAAATGA-ATTTACATGCAACACCCCTTAT

                                        1610      1620      1630      1640      1650      1660
Neopagetopsis ionah CoRe             TTACACGCCCCCTTAATTACCCCCCCCCCACTTTCTTACTCATACACCTTTGTTCAACAT
Neopagetopsis ionah ISP trnT-nad6    T-ATAGCTCTCAAAAGTCCCCCCGCGCCTGGGCCCTTATTCTCACCTCTCACCTCC----
```

GR-ISP *trnT*-*nad6* in *Pseudochaenichthys georgianus*
(fig. 5a; supplementary fig. S5, Supplementary Material online)

Pairwise alignment of GR-ISP *trnT*-*nad6* with a portion of *Pseudochaenichthys georgianus* CoRe

```
                                                     1070      1080      1090      1100      1110      1120
Pseudochaenichthys georgianus CoRe             CATACCCCACTTTCTTACTCACATGCCCCTTTGTTCAACATATGCCGCCCCTATACAGTG
Pseudochaenichthys georgianus ISP trnT-nad6    --------------ACCCCCCCCCCCATTTTCTCTACTTTAAACCAGGC--GCGCTGGG

                                                     1130      1140      1150      1160      1170      1180
Pseudochaenichthys georgianus CoRe             ATACAAATTTACACGCCCCCTTAACTGCCCCCCCCCCAGACGGGCACGTTTAAAAGCCT
Pseudochaenichthys georgianus ISP trnT-nad6    A-ACAAATTTGTCCGCCCCCTTAACT-CCCCCCCCCCGGATGGG-AGG---AAACAACA

                                                     1190      1200      1210      1220      1230      1240
Pseudochaenichthys georgianus CoRe             CGCACAATTTATACATATATAAGACCCCCTAACCACACAAATCTGTGTCCGTAACCAATG
Pseudochaenichthys georgianus ISP trnT-nad6    --AGTGA---ACTTACACGCAACACCCCATTATAATTCTCAAAGTCCCCCGCGCC--TG

                                                     1250      1260      1270
Pseudochaenichthys georgianus CoRe             TGCCACGAACACAAACAACCCCCCCCCCCC
Pseudochaenichthys georgianus ISP trnT-nad6    GGCCCTTATTCTCACCTTCCCCCCCCCC--
```

GR-ISP *trnT*-*nad6* in *Pagetopsis macropterus*
(fig. 5a; supplementary fig. S5, Supplementary Material online)

Pairwise alignment of GR-ISP *trnT*-*nad6* with a portion of *Pagetopsis macropterus* CoRe

```
                                                1080      1090      1100      1110      1120      1130      1140      1150
Pagetopsis macropterus CoRe             AAACCCCCCCCCCCACTTTTTACTCACCCACACCTTTATTCAATATATGCCGCCCCTATACAATGATACAAATTTATGC
Pagetopsis macropterus ISP trnT-nad6    GCCCCCCCCCCCCCACTTTTTACTCACCCACACCTTTATTCAATATATGCCGCCCCTATACAATGATACAAATTTATGC

                                                1160      1170      1180      1190      1200      1210      1220      1230
Pagetopsis macropterus CoRe             GCACCCTCCTCAACTGCCCCCCCCAACGGGGCAGAACAGCATGGTTAAACGCCTCACACAATTTATGCATATATAAGTCC
Pagetopsis macropterus ISP trnT-nad6    GCACCCTC---AACTGGCCTCC----CGGGGCAGAACAACA--GATAAAC------------TTACACATATACCC-CTT

                                                1240      1250      1260      1270      1280      1290
Pagetopsis macropterus CoRe             ATCTGACCACACAAATATATGTACTCATACCCAATGTACCACAAGCACAAACCAACCCCCCC
Pagetopsis macropterus ISP trnT-nad6    ATTGTAGTTCTCAAGAGTCCCCCAGCGCCTGAGCCCTTATTCTCACCTACATACCTCC--
```

GR-ISP *trnT*-*nad6* in *Chionodraco hamatus*
(fig. 5a; supplementary fig. S5, Supplementary Material online)

Pairwise alignment of GR-ISP *trnT*-*nad6* with a portion of *Chionodraco hamatus* CoRe

```
                                          890       900       910       920       930       940       950       960       970
Chionodraco hamatus CoRe             TTACTTCCCTATAATACCTAAGCCCTCACAAGCCACAAGAGCTAAAACTACTTGTACGTACTAGCACACCCCACTTACTTCCCTATAAT
Chionodraco_hamatus ISP trnT-nad6    --AACCCCCACAAT--TTCATCCCCTTTAA-CC--AGTCTCCACTGGTT-TTTACACG-GCCCCCACTTCCGACTAACC-CCCCACAAT

                                          980       990      1000      1010      1020      1030      1040      1050      1060
Chionodraco hamatus CoRe             ACCTAAGGCCTCACAAGCCACAAGAGCTAAAAACTACTTGTACGTACTAGCACACCCCCACTTACTTCCCTATAATACCTAA-GGCCTCA
Chionodraco_hamatus ISP trnT-nad6    TTC--A-TCCCCTTTAACCA----GTCT-----CCACTGGT--TT-TTA-CACGCCCCC---CACTTCCGACTAACCCCCCACAATTTCA

                                         1070      1080      1090      1100      1110      1120      1130      1140      1150
Chionodraco hamatus CoRe             CAAGCCACAAGAGCTAAAAACTACTTGTACGTACTAGCACACCCCACTTACTTCCTCCCGCCCACTTTTGT--TTGACATACGCCCCCTA
Chionodraco_hamatus ISP trnT-nad6    T---CCCCTTTAACCAGTCTCCACTGGTTTTTAC--ACGGCCCCCACTT-CCCCCCCCACCC-CTATTGTACCTCTCA-AGGGCCCCCA

                                         1160      1170      1180      1190      1200
Chionodraco hamatus CoRe             TACAG-TGATACAATTTACCCTCACCCTTACTGCACTTCCACAGCCCCTA
Chionodraco_hamatus ISP trnT-nad6    CACGCCTGAGCC--CTTACCCTCGCCTCCA----ACCCCCC--CCCCT-
```

Supplementary Figure S13

Alignments of genes involved in genomic rearrangements and the associated intergenic spacers (GR-ISP). The alignments were performed with the ClustalW program, available at the PRABI/Rhone-Alpes Bioinformatics Center (https://npsaprabi.ibcp.fr/cgi-bin/npsa_automat.pl?page=/NPSA/npsa_server.html). Successively, the alignments were manually improved through visual inspection. rc_sequence_name = reverse complement sequence of a gene encoded on the L-strand.

GR-ISP ***trnT*-*nad6*** in *Chionodraco rastrospinosus*

(fig. 5a; supplementary fig. S5, Supplementary Material online)

Pairwise alignment of GR-ISP ***trnT*-*nad6*** with a portion of *Chionodraco rastrospinosus* **CoRe**

```
                                                790       800       810       820       830       840       850       860       870
                                                  |         |         |         |         |         |         |         |         |
Chionodraco rastrospinosus CoRe         TATACGTACTAGTGCCCCCCACTTACTTCCCTATAATACCTAAGGCCTCACAGGCCCCAAAAGCTAAAAACTACTGGTACGTACTAATAC
Chionodraco rastrospinosus ISP trnT-nad6 ----------------------------------ACCCCCCACAATTTCAT---CCCCTTTAACCAGTCTCCACTGGTTTTTAC----AC

                                                880       890       900       910       920       930       940       950       960
                                                  |         |         |         |         |         |         |         |         |
Chionodraco rastrospinosus CoRe         ACCCCCCACTTACTTCCCTGTAATACCTAAGCCCTCACAAGCCACAAGAGCTAAAAACTACTTGTACGTACTAGCACACC-CCACTTACT
Chionodraco rastrospinosus ISP trnT-nad6 GGCCCCC-C--ACTTCC--GA----C-TAACCCCCCACAA-TTTCATCCCCTTTAACCAGTCTCCACTGGTTTTTACACGGCCCCCCACT

                                                970       980       990      1000      1010      1020      1030      1040      1050
                                                  |         |         |         |         |         |         |         |         |
Chionodraco rastrospinosus CoRe         TCCCTATAATACCTAAGGCCTCACAAGCCACAAGAGCTAAAAACTACTTGTACGTACTAGCACACCCCCCACTTACTTCCCTATAATACC
Chionodraco rastrospinosus ISP trnT-nad6 TCC-------GACTAACCCCCCACAA-TTTCATCCCCTTTAACCAGTCTCCACTGGTTTTTACACGGCCC-CCCACTTCCGACTAACCCC

                                               1060      1070      1080      1090      1100      1110      1120      1130      1140
                                                  |         |         |         |         |         |         |         |         |
Chionodraco rastrospinosus CoRe         TAAGGCCTCACAAGCCACAAGAGCTAAAAACTACTTGTACGTACTAGCACACCCCCCACTTACTTCCTCCCCCCCACTTTTGTTTGACA
Chionodraco rastrospinosus ISP trnT-nad6 CCACAATTT-C-ATCCCCTTGAACCAGTCTCCACTGGTTTTTAC----ACGGCCCCCCACTT-C--CCCCCCCACCCCTTTTGTTCCTCT

                                               1150      1160      1170      1180      1190      1200
                                                  |         |         |         |         |         |
Chionodraco rastrospinosus CoRe         TAC-GCCCCCTATACAGTGATACAATTTACCCTCACCCTTACTGCACTTCCACAGCCCCT
Chionodraco rastrospinosus ISP trnT-nad6 CAAGGGCCCCCACACGCCTGAGCC-CTTACCCTCGCCTCCA----ACCCCCCCCCCCT-
```

GR-ISP ***trnT*-*trnEa*** in *Gymnodraco acuticeps*

(fig. 5b; supplementary fig. S4 and fig. S15, Supplementary Material online)

Gymnodraco acuticeps ISP ***trnT*-*trnEa***

CAAAAACAAAACCAAGCCCCCCCCCCCACATTCCCGGCCCCCACCCCTACCCCCCCCCCACACTCCCGACCCCTGCCCCCCAATTTTTACCACTTTTTATCTATTTCAACTGGTGCTTTAAATATTCCCCCCA
CATTACCGGCACCCCCACCCCTATTCCCACCACCACGCCCAACATAAATAGATATCCGCAGTATAACATAATCCTTGCAAGGGCTCTAACCAAGACTAAGAGCATAAATATAGCCCTCGTTGTATTCAACTAC
AGTGAGCCCCCATCATACCCATGTATGACACACAAGCTCTTTCACTGCGATTGTTTTTTCACCCCTCCGTCATGCCCTGCCCCCACCAATTATTCACGTATCCCAAGCCCACAGTAGTAAGATAAACCCCCAT
AGTACCCCGAAGTACCCCCCAATTCACTTATATACCCACCACTCCCCCCCGCAAACCTGATGAGCCTTAGCATATAAATCTTCTCAACCAGTAAAAACGGCCAACATGGTCCCTAAAAAAGTTAAAAGACAAA
GCTACTCCTAAAAGAGCACCTCACAGTCAAGTAATAAATCCTCCACCTGCTCCCGCTACCAAGTACATTCCTAGTAACGGTCAAAGTAGGGTGGGATGGGTTTCTACTACTATCAATCCAATCGACGCGCTCA
TTATGAAGAAAAACAGAAAGTCTAACAT

rc_Gymnodraco_acuticeps_***nad6***

CCTTTTAATGGGCCGTACCGCTCCCCGCCCGCTGCCCCGCAACACTTCCAAAGCAACACATAACGCCACCAGCAAAGCTCACCCACAAACAACTAAAAAAATACCCCCACTTCCATATGCTTCTGACACCCCA
TCGATCTCCCCTTGCACTACCCCCACCTCTGTCTCCTCCCCCACAAACCACCAATACGGAGGGGCCTCCAAACTCGCCAGAGAAAAAGTTACCCCCATTACTGCCCCAAAGTACCCGACTATTCACTTAAACA
CCGACCCCTTGGTCAAACCTGTTGGGTAAGCTTCCGCACATAACGCTGCCGAGTAAGCAAACACAACCAGCATCCCCCCTAGATAAATTAAGAATAGGACTAAGCATAAAAAAGTGCCCCCAACCCATATGAT
AAACCCACACCCCGCTGCTGCAACCGAAACCACCCCTAGCGCCGCATAGAAGGGGGACGGATTAGCGGCAACCACCACTATTCCCACCACCACGCCCAACATAAATAGATATCCGCAGTATAACAT

Gymnodraco acuticeps ***nad6***

ATGTTATACTGCGGATATCTATTTATGTTGGGCGTGGTGGTGGGAATAGTGGTGGTTGCCGCTAATCCGTCCCCCTTCTATGCGGCGCTAGGGGTGGTTTCGGTTGCAGCAGCGGGGTGTGGGTTTATCATAT
GGGTTGGGGGCACTTTTTTATGCTTAGTCCTATTCTTAATTTATCTAGGGGGGATGCTGGTTGTGTTTGCTTACTCGGCAGCGTTATGTGCGGAAGCTTACCCAACAGGTTTGACCAAGGGGTCGGTGTTTAA
GTGAATAGTCGGGTACTTTGGGGCAGTAATGGGGGTAACTTTTTCTCTGGCGAGTTTGGAGGCCCCTCCGTATTGGTGGTTTGTGGGGGAGGAGACAGAGGTGGGGGTAGTGCAAGGGGAGATCGATGGGGTG
TCAGAAGCATATGGAAGTGGGGGTATTTTTTTAGTTGTTTGTGGGTGAGCTTTGCTGGTGGCGTTATGTGTTGCTTTGGAAGTGTTGCGGGGCAGCGGGCGGGGAGCGGTACGGCCCATTAAAAGG

Gymnodraco acuticeps **NAD6**

MLYCGYLFMLGVVVGMVVVAANPSPFYAALGVVSVAAAGCGFIMWVGGTFLCLVLFLIYLGGMLVVFAYSAALCAEAYPTGLTKGSVFKWMVGYFGAVMGVTFSLASLEAPPYWWFVGEETEVGVVQGEIDGV
SEAYGSGGIFLVVCGWALLVALCVALEVLRGSGRGAVRPIK

Gymnodraco acuticeps ISP ***trnT*-*trnEa*** (**segment A**)

TATTCCCACCACCACGCCCAACATAAATAGATATCCGCAGTATAACAT

Peptide encoded by segment A (reverse complement)

MLYCGYLFMLGVVVGM

Pairwise alignment of the peptide encoded (reverse complement) by segment A with a portion of the **NAD6** of *Gymnodraco acuticeps*

```
                                  10        20
                                   |         |
Gymnodraco acuticeps NAD6       MLYCGYLFMLGVVVGMVVVA
Peptide encoded by segment A    MLYCGYLFMLGVVVGM----
```

Identical amino acid; strongly similar amino acid; weakly similar amino acid.

Supplementary Figure S14

Alignments of genes involved in genomic rearrangements and the associated intergenic spacers (GR-ISP). The alignments were performed with the ClustalW program, available at the PRABI/Rhone-Alpes Bioinformatics Center (https://npsaprabi.ibcp.fr/cgi-bin/npsa_automat.pl?page=/NPSA/npsa_server.html). Successively, the alignments were manually improved through visual inspection. rc_sequence_name = reverse complement sequence of a gene encoded on the L-strand.

GR-ISP *trnT*-*trnEa* in *Gymnodraco acuticeps*
(fig. 5b; supplementary fig. S4 and fig. S14, Supplementary Material online)

Gymnodraco acuticeps ISP ***trnT*-*trnEa*** (**segment B**)
ACCAATTATTCACGTATCCCAAGCCCACAGTAGTAAGATAAACCCCCATAGTACCCCGAAGTACCCCCCAATTCACTTATATACCCACCACTCCCCCCCGCAAACCTGATGAGCCTTAGCATATAAATCTTCT
CAACCAGTAAAAACGGCCAACATGGTCCCTAAAAAAGTTAAAAGACAAAG

Peptide encoded by segment B (reverse complement)
LCLLTFLGTMLAVFTGWEDLYAKAHQVCGGEWWVYKWIGGYFGVLWGFILLLWAWDTWMIG

Pairwise alignment of the peptide encoded (reverse complement) by segment B with a portion of the **NAD6** of *Gymnodraco acuticeps*

```
                                       10        20        30        40        50        60
                                        |         |         |         |         |         |
Gymnodraco acuticeps NAD6        MLYCGYLFMLGVVVGMVVVAANPSPFYAALGVVSVAAAGCGFIMWVGGTFLCLVLFLIYL
Peptide encoded by segment B     --------------------------------------------------LCL---LTFL

                                       70        80        90       100       110       120
                                        |         |         |         |         |         |
Gymnodraco acuticeps NAD6        GGMLVVFAYSAALCAEAYPTGLTKGSVFKWMVGYFGAVMGVTFSLASLEAPPYWWFVGEE
Peptide encoded by segment B     GTMLAVFTGWEDLYAKAHQVCGGEWWVYKWIGGYFGVLWGFILLLWAWDT----WMIG--
```

Identical amino acid; strongly similar amino acid; weakly similar amino acid.

Gymnodraco acuticeps ISP ***trnT*-*trnEa*** (**segment C**)
CAGTCAAGTAATAAATCCTCCACCTGCTCCCGCTACCAAGTACATTCCTAGTAACGGTCAAAGTAGGGTGGGATGGGTTTCTACTACTATCAATCCAATCGACGCGCTCATTATGAAGAAAAACAGAAAGTCT
AACAT
>peptide_encoded_by_segment_C (reverse complement)
MLDFLFFFMMSASIGLMVVETHPTLLWPLLGMYLVAGAGGGFITWL

Pairwise alignment of the peptide encoded (reverse complement) by segment C with a portion of the **NAD6** of *Gymnodraco acuticeps*

```
                                       10        20        30        40        50        60
                                        |         |         |         |         |         |
Gymnodraco acuticeps NAD6        MLYCGYLFMLGVVVGMVVVAANPSPFYAALGVVSVAAAGCGFIMWVGGTFLCLVLFLIYL
Peptide encoded by segment C     MLDFLFFFMMSASIGLMVVETHPTLLWPLLGMYLVAGAGGGFITWL--------------
```

Identical amino acid; strongly similar amino acid; weakly similar amino acid.

Gymnodraco acuticeps **ψ*trnEa*** (**pseudo-*trnE*, TTT wrong anticodon (*trnK*)**)
GCTCACTGTAGTTGAATACAACGAGGGCTATATTTATGCTCTTAGTCTTGGTTAGAGCCCTTGCAAGGATT
AATCCTTGCAAGGGCTCTAACCAAGACTAAGAGCATAAATATAGCCCTCGTTGTATTCAACTACAGTGAGC

Multiple alignment of *trnE* and ψ*trnE* of selected species of Notothenioidei, based on the secondary structure predicted with tRNA-scan program.

```
                                                     10        20        30        40        50        60
                                                      |         |         |         |         |         |
                                             1234-567 1234     4321 12345  ant 54321   12345     543217654321d
Bovichtus angustifrons trnE                  GTTC-TGGTAGTTGAAGT-ACAACGATGGTT-T-TTCAAGCCATTAGTCTTGGTTAAAGTCCTGGCTGGAATA
Eleginops maclovinus KY038381 trnE           GTTT-TTGTAGTTGAA-TAACAACGATGGTT-T-TTCAAGCCATTAGTCCTGGTTAAAGTCCTGGGAGAAACC
Pleuragramma antarctica JF933905 trnE        GTTC-TTGTAGTTGAA-TAACAACGATGGCT-T-TTCATGCCGTTAGTCCTGGTTAAAATCCTGGCAAGAATT
Dissostichus eleginoides AB723627 trnE       GTTC-CTGTAGTTGAA-TAACAACGATGGCT-T-TTCATGCCGCAGTCCCGGTTAAAGTCCTGGCAAGAGTT
Dissostichus mawsoni LC138011 trnE           GTTC-CTGTAGTTGAA--AACAACGATGGCT-T-TTCATGCCGCAGTCCTGGTTAGAATCCTGGCAAGAATT
Lindbergichthys nudifrons trnE                GTTC-TTGTAGTTGAA-T-ACAACAATGGCT-T-TTCATGCCATGGGTCCGGGTTAAAGCCCTGGCAAGAATT
Akarotaxis nudiceps trnE                     GTTC-CTGTAGTTGAA-TAACAACGATGGCT-T-TTCGTGCCTTTAGTCCTGGTTAGAGTCCTGGTAAGAATT
Notothenia coriiceps JF933906 trnE           GTTC-TTGTAGTTGAA-TAACAACGATGGCT-T-TTCATGTCCTTAGTCCTGGTTAGAGTCCTGGTGAGAATC
Racovitzia glacialis GU214222 trnE           GTTC-CTGTAGTTGAA-TAACAACGATGGCT-T-TTCATGTCTTTAGTCCTGGTTAGAGTCCTGGCAAGAATT
Gerlachea australis trnE                     GTTC-CTGTAGTTGAA-T-ACAACGATGGCT-C-TTCATGCCTTTAGTCCTGGTTAGAGTCCTGGCAAGAATT
Gymnodraco acuticeps trnEb                   GTTC-TTGTAGTTGAA-T-ACAACGATGGCT-T-TTCATGCCTTTAGTCCTGGTGAGAGTCCTGGGGAGAATT
Gymnodraco acuticeps trnEa                   GTTC-TTGTAGTTGGA-T-ACAACGATGGCT-T-TTCATGCTTTTAGTCCTGGTGAGAGTCCTGGGGAGAATT
Gymnodraco acuticeps ψtrnEa                  GCTCACTGTAGTTGAA-T-ACAACGAGGGCTATATTTATGCTCTTAGTCTTGGTTAGAGCCCTTGCAAGGATT
Pagetopsis macropterus trnE                  GTTC-TTGTAGTTGAA-CGACAACGATGGCT-T-TTCATGTCTTTAGTCCTGGTTAAAACCCTGGCAAGAATT
Chaenodraco wilsoni trnE                     GTTC-CTGTAGTTGAA-TAACAACGATGGCT-T-TTCATGCCATTAGTCCTGGTTAGAGTCCTGGCAAGAATC
                                                                                      *         *
                                             1234-567 1234     4321 12345  ant 54321   12345     543217654321d
                                             0000-000 1111     1222 22222  333 44444   45555     6666666667777
                                             1234-567 0123     9012 45678  123 01234   90123     1234567890123
```

Legend

N, the background base for the position.
N, half-compensatory base change in the stem pair (e.g. T – G vs C – G; A-T vs G-T).
N, N half-compensatory base change in the stem pair exhibiting a mismatch (e.g. T-A vs A-A).
N, N, fully compensatory base change in the stem pair exhibiting a mismatch (e.g. C-G vs T-T).
N, N, type I fully compensatory base change in the stem pair (i.e. purine – pyrimidine vs purine – pyrimidine, e.g. G – C vs A – T).
N, N, type II fully compensatory base change in the stem pair (i.e. purine – pyrimidine vs pyrimidine – purine, e.g. A – T vs T – A).
Different colors are used to better differentiate the changes.
N, a mismatch in the in the stem pair; N, a mismatch in a pair not always fully consistent (e.g. 5 5).
N, substitution pattern not modelled.
*, position in the stem characterized by a mismatch.
N, N, molecular signature for a taxon.
X, position 1-7 in the acceptor stem; X position 1-4 in the DHU stem; X position 1-5 in the anticodon stem; X position 1-5 in the TΨC stem; ant, anticodon; d, discriminator nucleotide
A compensatory base change implies the substitution of a nucleotide with a different base that does not disrupt the pairing in the stem.

(See Montelli et al. 2016)

Montelli S, Peruffo A, Patarnello T, Cozzi B, Negrisolo E. 2016. Back to water: signature of adaptive evolution in Cetacean mitochondrial tRNAs. PLoS ONE 11:e0158129.

Supplementary Figure S15

Alignments of genes involved in genomic rearrangements and the associated intergenic spacers (GR-ISP). The alignments were performed with the ClustalW program, available at the PRABI/Rhone-Alpes Bioinformatics Center (https://npsaprabi.ibcp.fr/cgi-bin/npsa_automat.pl?page=/NPSA/npsa_server.html). Successively, the alignments were manually improved through visual inspection. rc_sequence_name = reverse complement sequence of a gene encoded on the L-strand.

GR-ISP ***trnP*$_b$-*trnF*** in *Gymnodraco acuticeps*
(fig. 5b; supplementary fig. S4, Supplementary Material online)

Pairwise alignment of GR-ISP ***trnP*$_b$-*trnF*** with a portion of *Gymnodraco acuticeps* **CoRe**

```
                                                10        20        30        40        50        60        70        80        90
                                                 |         |         |         |         |         |         |         |         |
Gymnodraco acuticeps CoRe                 TACACACATCAGTACAGCTATGTATTATCAACACATCAGTACAGCTATGTATTATCAACACTCATTTATATT-A-ACCATTTCATGAT-C
Gymnodraco acuticeps ISP trnPb-trnF       TACACACATCAGTACAGCC---------CAACACTTTTGCTTA--TATTAAATTCCTCCTCCCTTTATAGTGATACCAAATCATAATAC

                                               100       110       120       130       140       150       160       170       180
                                                 |         |         |         |         |         |         |         |         |
Gymnodraco acuticeps CoRe                 ATTCGTGAACAAT----CCGTGATTTTTTTGTTAAACTGGCTTTAACACTAACACACATCAAAACCGACAGTAGGCATACAT-TAAGCAT
Gymnodraco acuticeps ISP trnPb-trnF       ACCCCTAAACACCCGCCCCACTACCCCCTTACTAAGCTG--TTCAA-A--AAC-CTCCT-ACAATTTATA-CATGTATACACACACACAT

                                               190       200       210       220       230       240       250       260       270
                                                 |         |         |         |         |         |         |         |         |
Gymnodraco acuticeps CoRe                 TTGAGAA-GCCTCCATTGACCTAGCTTTAAGAGCAGACGAAAATTTAAGACCGAACACTTTATCCATAAGTTAAGTTATACGTTTATTTA
Gymnodraco acuticeps ISP trnPb-trnF       TTGAGTGTGCCCACAT-AAACTA----TATGT-C--ACGAATAC--AC-ACC---CGCCCACCCCCTTTT---TTTGGCGGCCTTCTC

                                               280       290       300       310       320
                                                 |         |         |         |         |
Gymnodraco acuticeps CoRe                 GCATCCGACAGACTCTAACCTTATAAGCGCAGTAAGAGCCGACCTACAAG
Gymnodraco acuticeps ISP trnPb-trnF       ACATC----AGCCCCTAAACC-A-AAGCACC-T--GGGCTATTCT-C---
```

Supplementary Figure S16

Alignments of genes involved in genomic rearrangements and the associated intergenic spacers (GR-ISP). The alignments were performed with the ClustalW program, available at the PRABI/Rhone-Alpes Bioinformatics Center (https://npsaprabi.ibcp.fr/cgi-bin/npsa_automat.pl?page=/NPSA/npsa_server.html). Successively, the alignments were manually improved through visual inspection. rc_sequence_name = reverse complement sequence of a gene encoded on the L-strand.

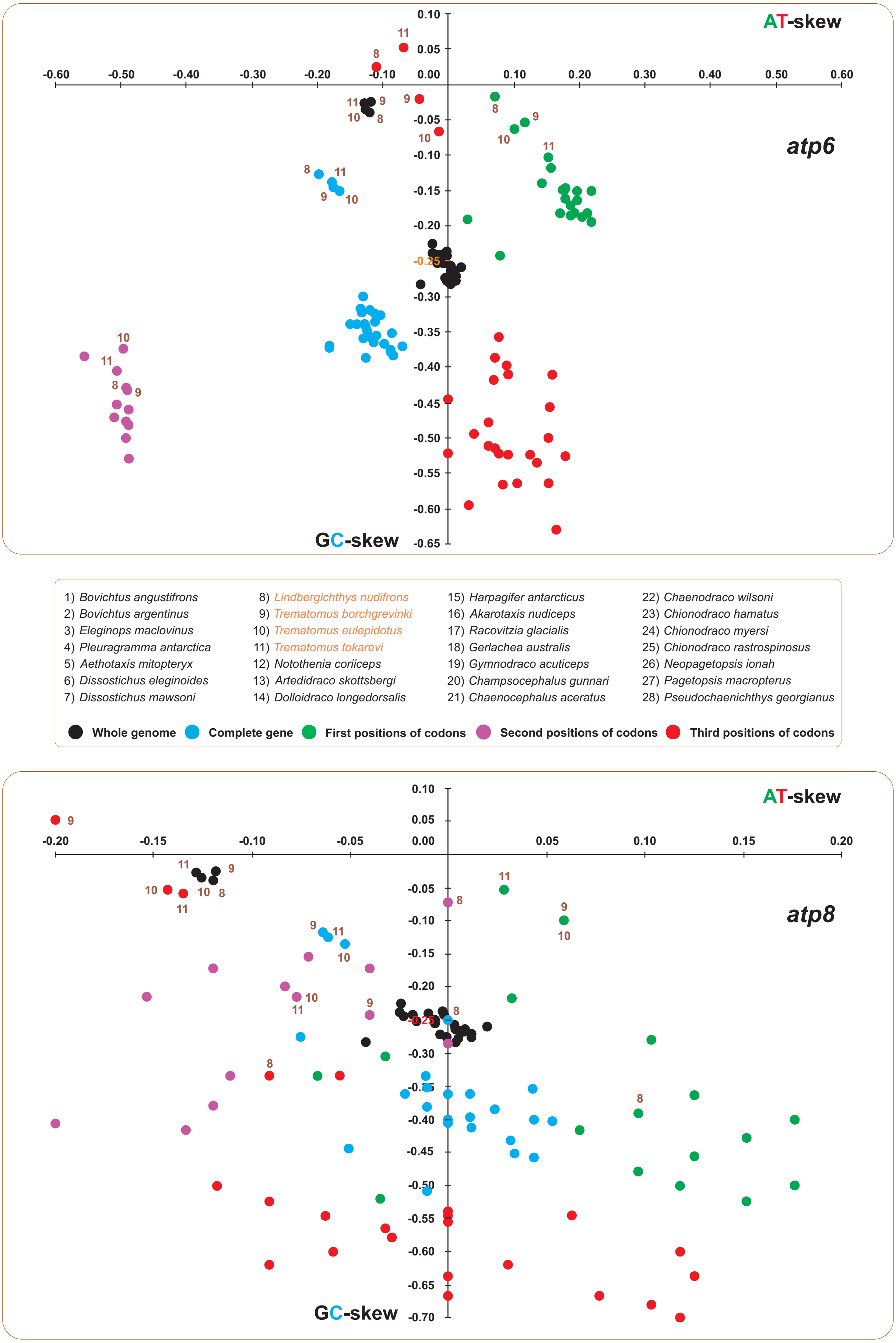

Supplementary Figure S17. **AT**-skew (x axis) vs **GC**-skew (y axis) scatter plot for the genes ***atp6*** and ***atp8***. **AT**-skew = (**A**-**T**)/(**A**+**T**); GC-skew = (**G**-**C**)/(**G**+**C**). Numbers identify species belonging to Trematominae.

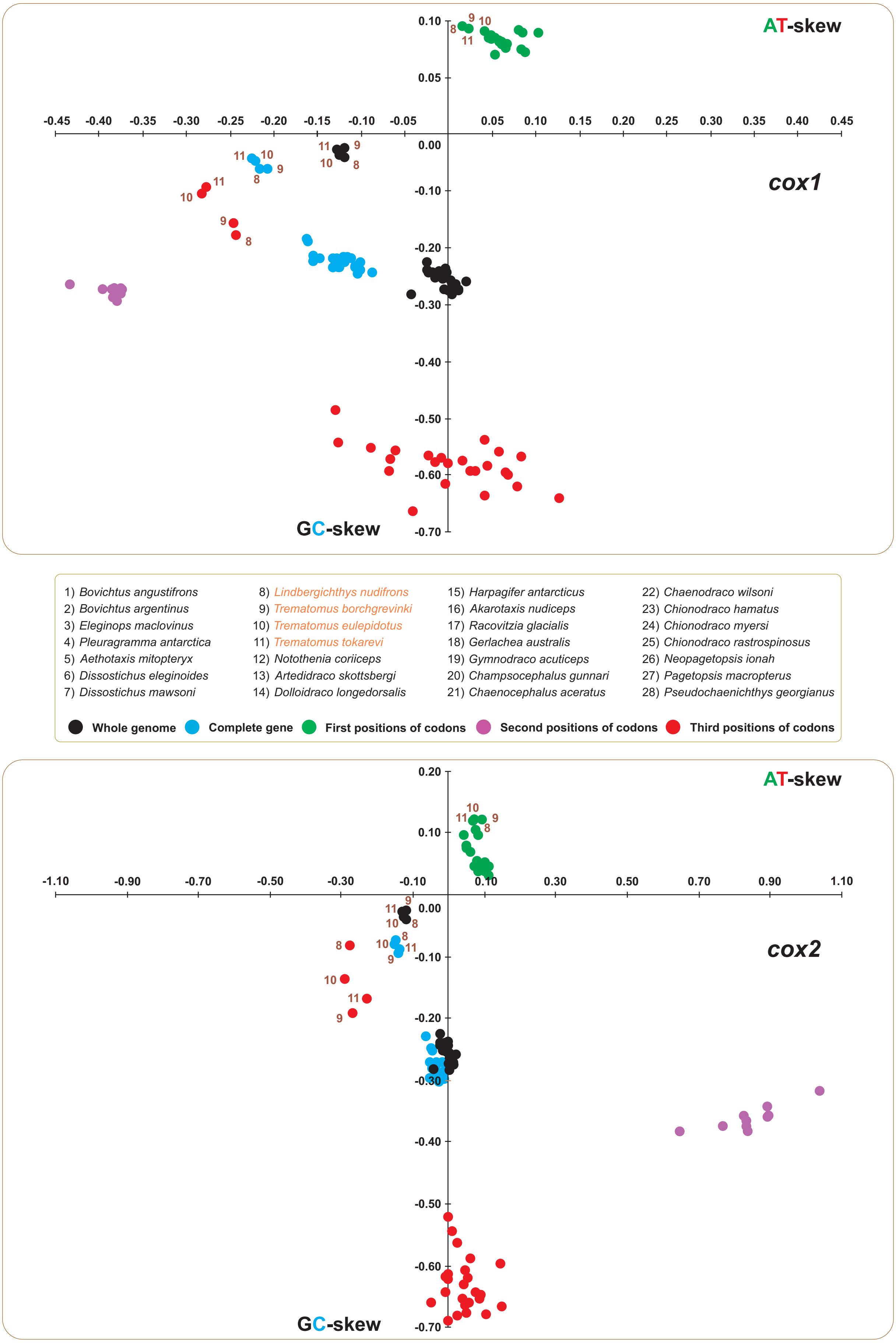

Supplementary Figure S18. AT-skew (x axis) vs GC-skew (y axis) scatter plot for the genes ***cox1*** and ***cox2***.
AT-skew = (**A**-**T**)/(**A**+**T**); GC-skew = (**G**-**C**)/(**G**+**C**). Numbers identify species belonging to Trematominae.

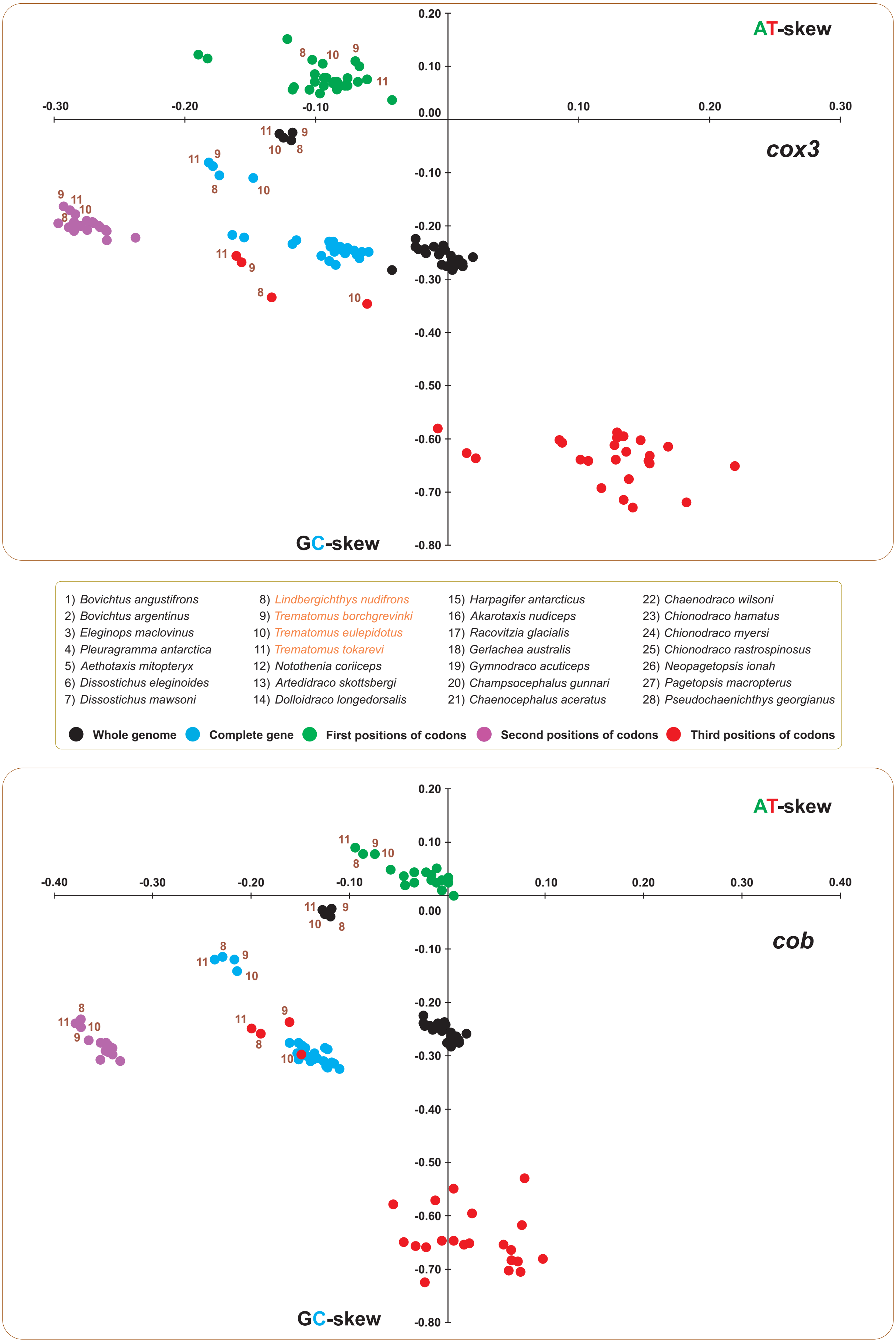

Supplementary Figure S19. AT-skew (x axis) vs GC-skew (y axis) scatter plot for the genes ***cox3*** and ***cob***. AT-skew = (**A**-**T**)/(**A**+**T**); GC-skew = (**G**-**C**)/(**G**+**C**). Numbers identify species belonging to Trematominae.

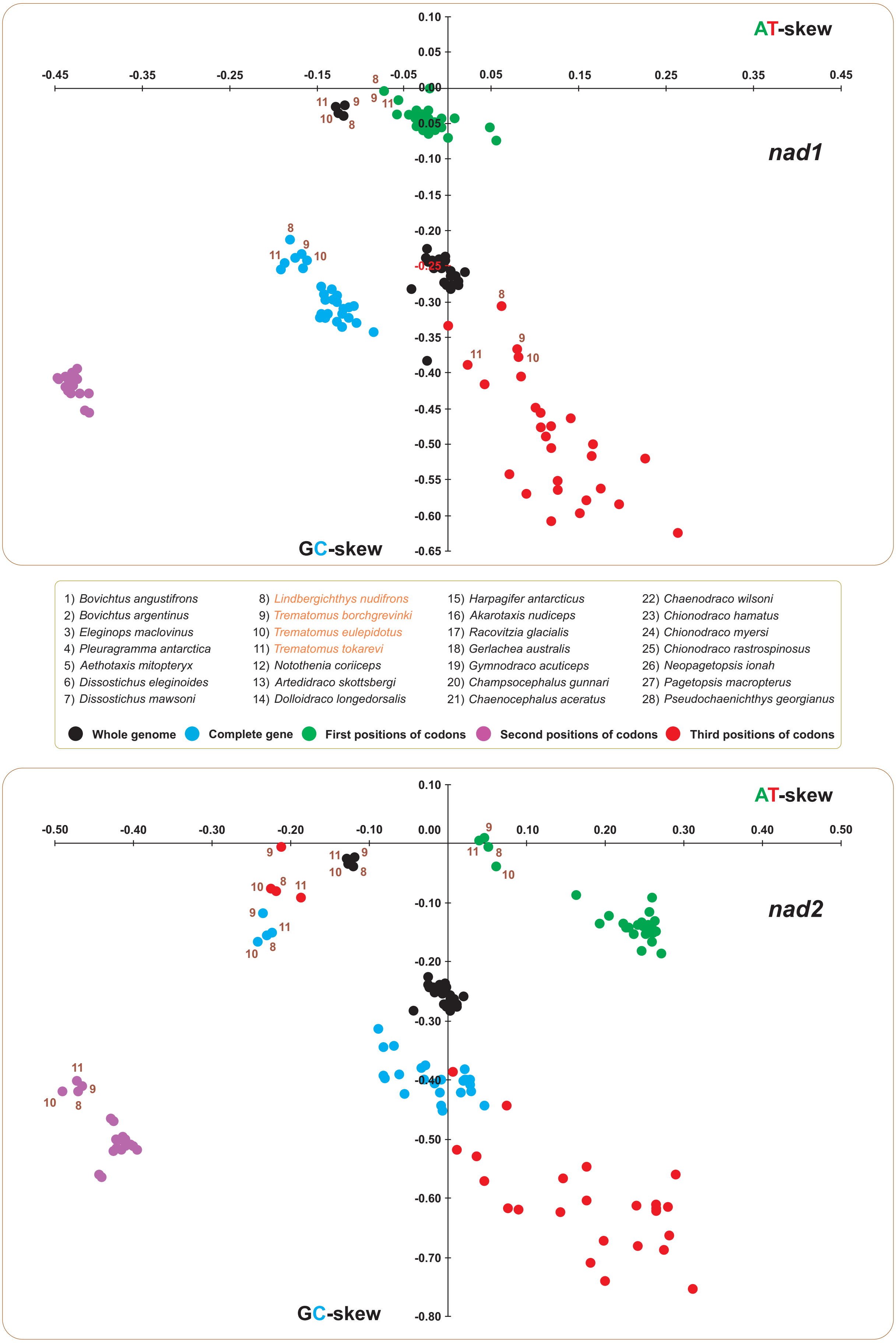

Supplementary Figure S20. AT-skew (x axis) vs GC-skew (y axis) scatter plot for the genes ***nad1*** and ***nad2***. AT-skew = (A-T)/(A+T); GC-skew = (G-C)/(G+C). Numbers identify species belonging to Trematominae.

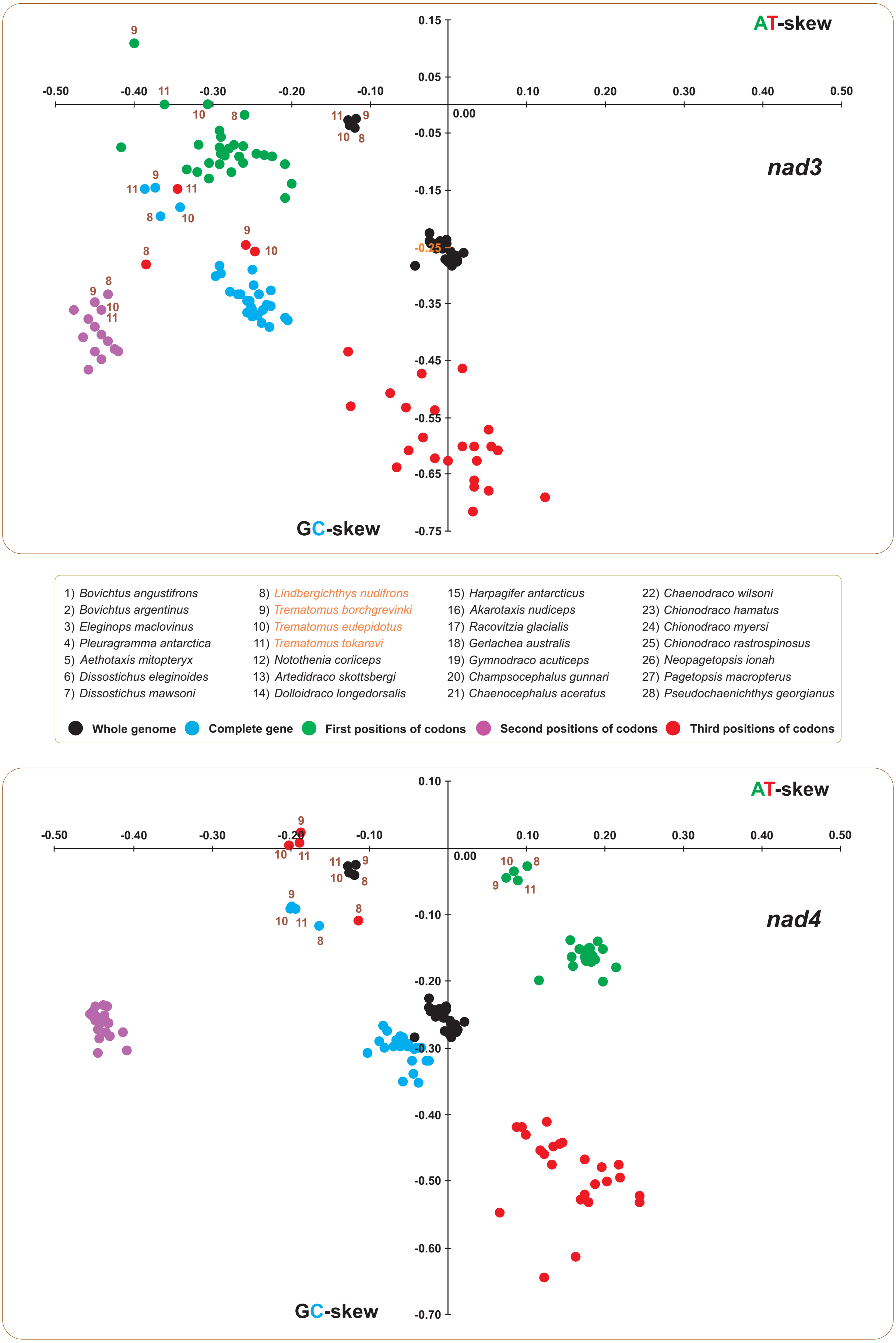

Supplementary Figure S21. AT-skew (x axis) vs GC-skew (y axis) scatter plot for the genes ***nad3*** and ***nad4***. AT-skew = (A-T)/(A+T); GC-skew = (G-C)/(G+C). Numbers identify species belonging to Trematominae.

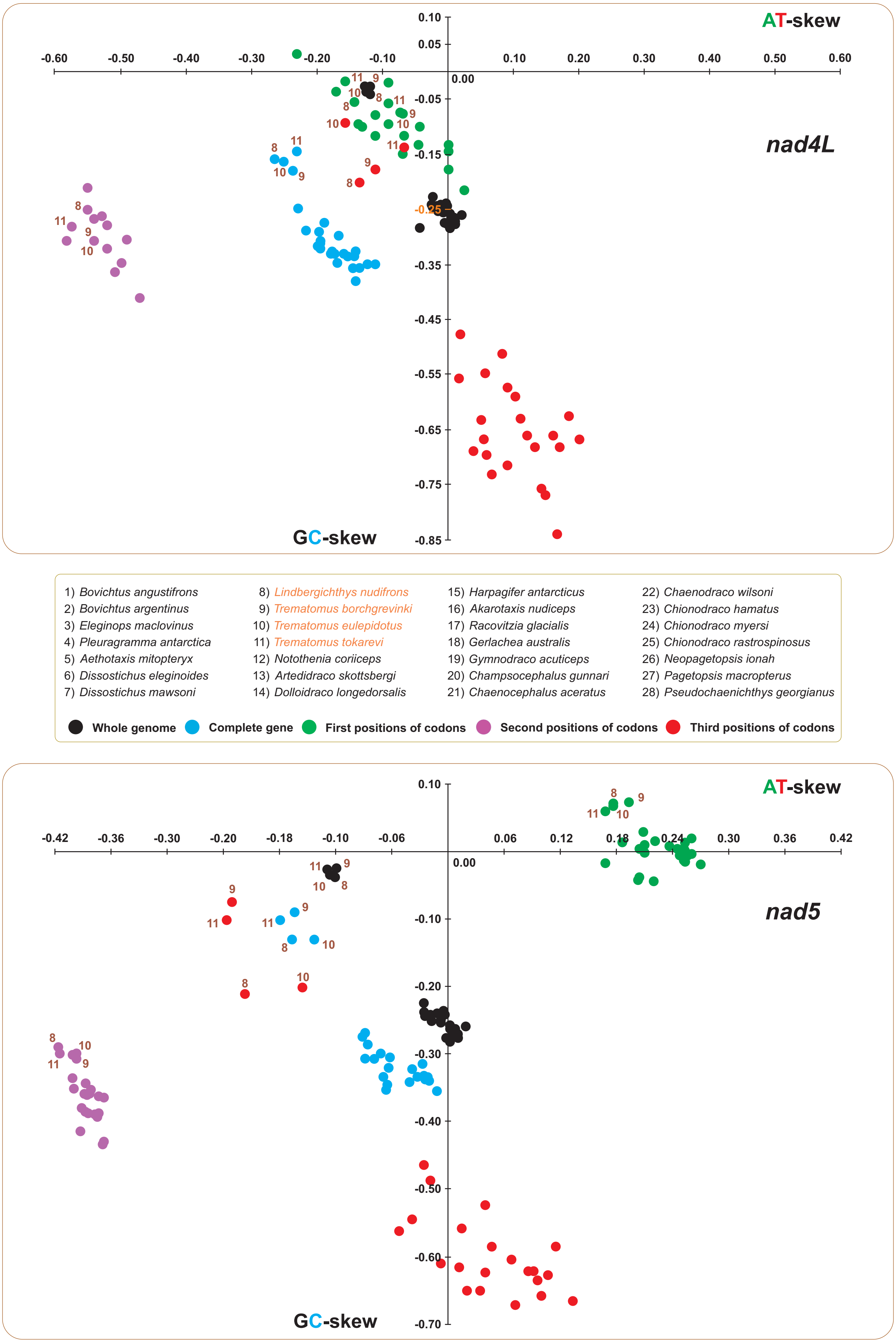

Supplementary Figure S22. AT-skew (x axis) vs GC-skew (y axis) scatter plot for the genes ***nad4L*** and ***nad5***. AT-skew = (A-T)/(A+T); GC-skew = (G-C)/(G+C). Numbers identify species belonging to Trematominae.

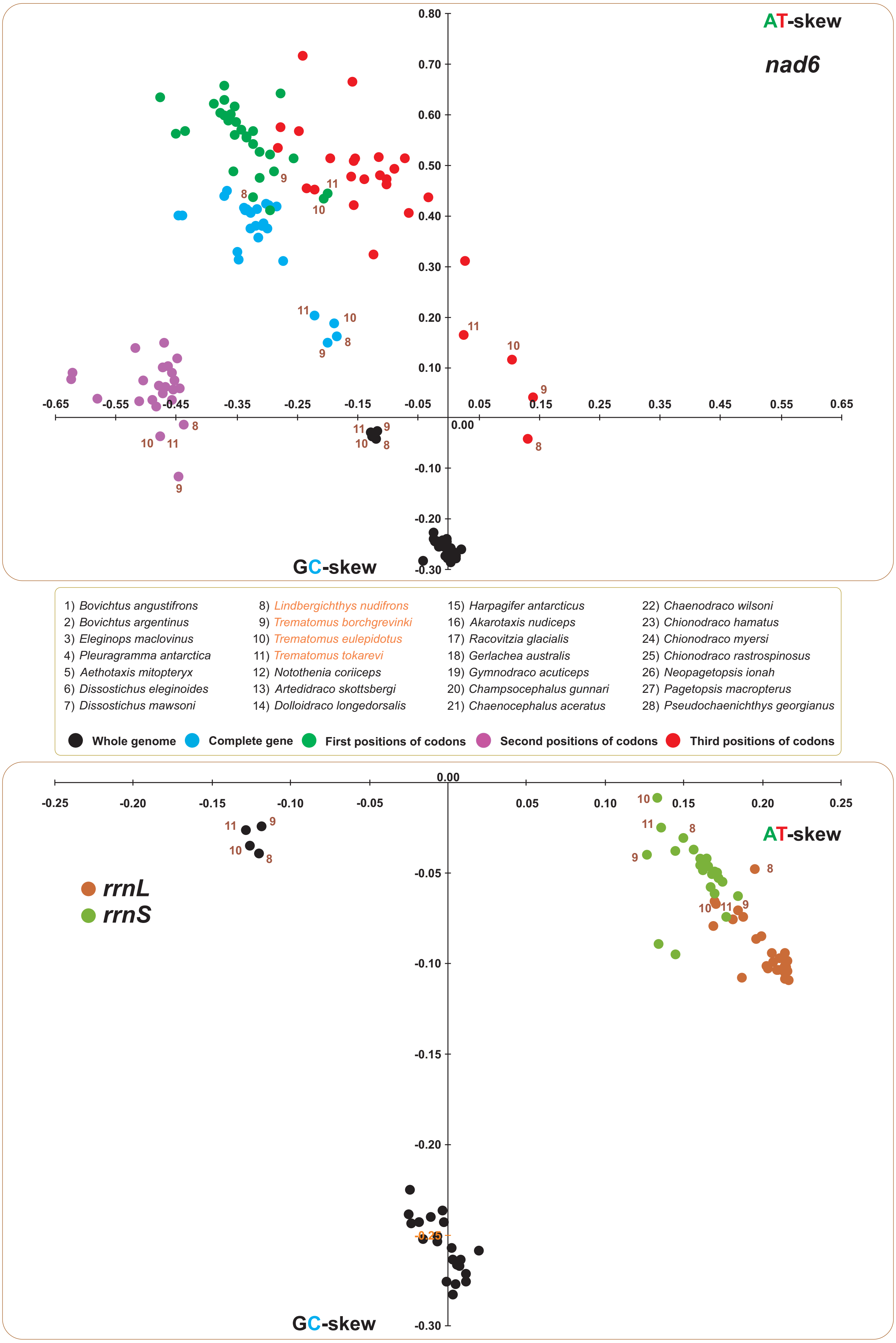

Supplementary Figure S23. AT-skew (x axis) vs GC-skew (y axis) scatter plot for the genes ***nad6, rrnL*** and ***rrnS***. AT-skew = (**A**-**T**)/(**A**+**T**); GC-skew = (**G**-**C**)/(**G**+**C**). Numbers identify species belonging to Trematominae.

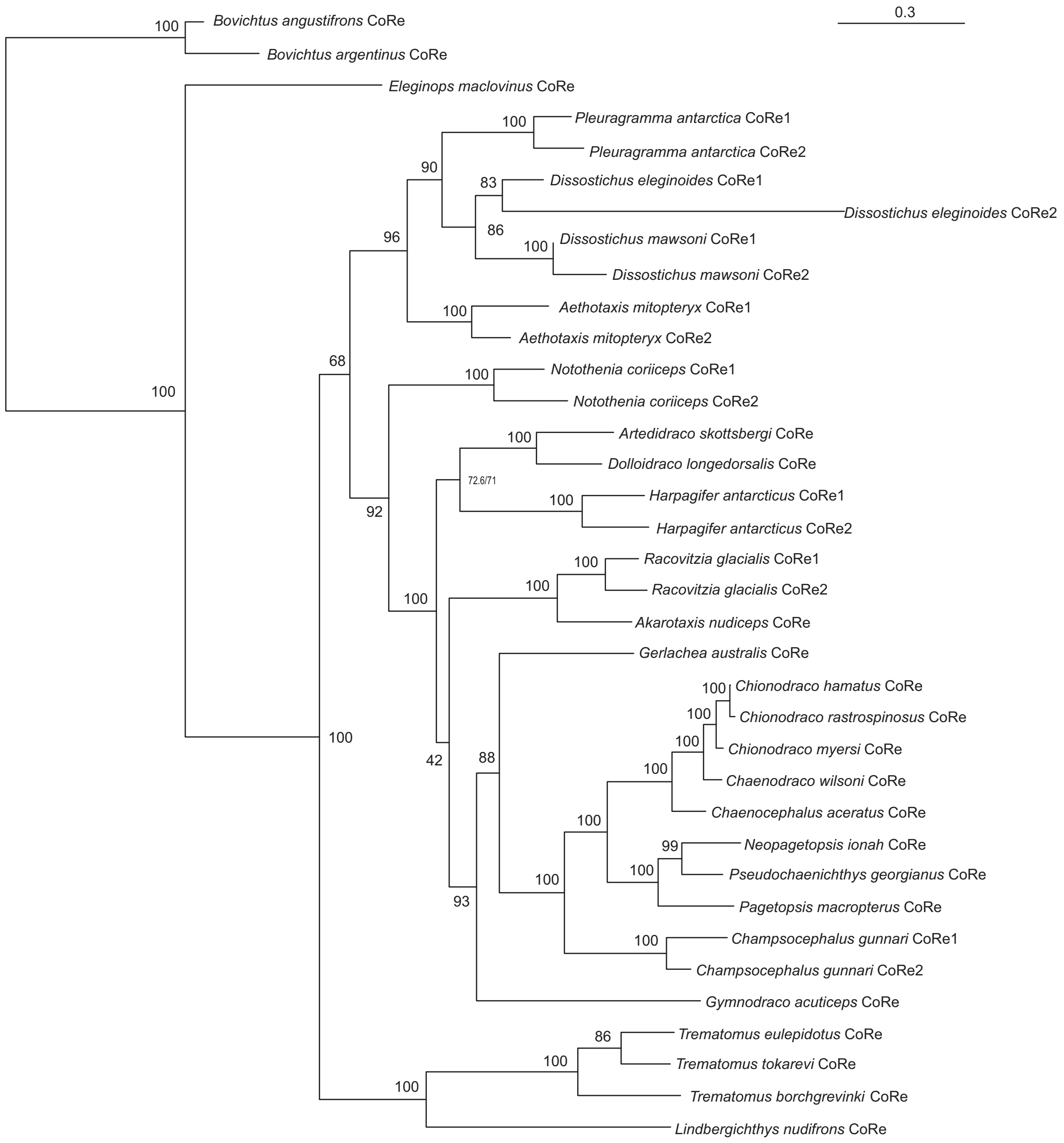

Supplementary Figure S24. Maximum Likelihood tree (-ln = 32500.1830) obtained from the analysis performed on the CoRe multiple alignment by applying the TN+F+I+G4 model. Ultrafast bootstrap values are provided for each node. The scale bar represents 0.3 substitutions/site. For details on the applied phylogenetic strategy, see supplementary Materials Methods, Supplementary Material online.

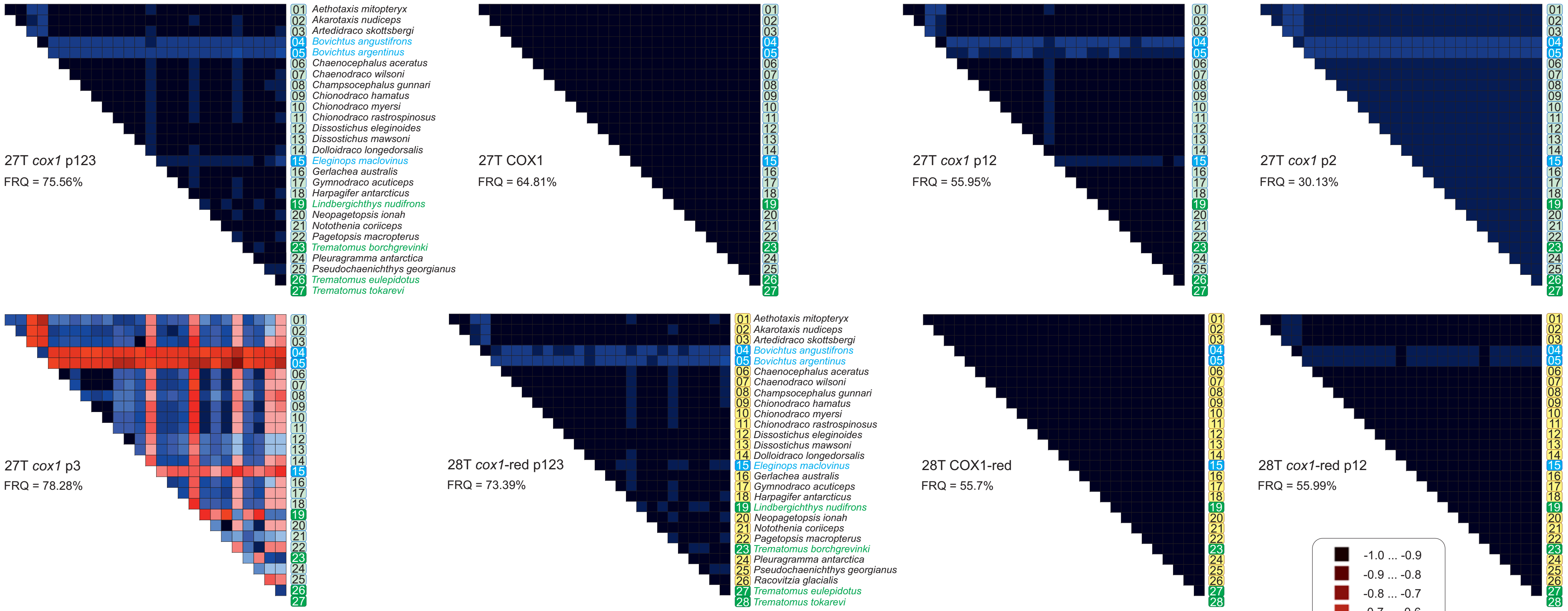

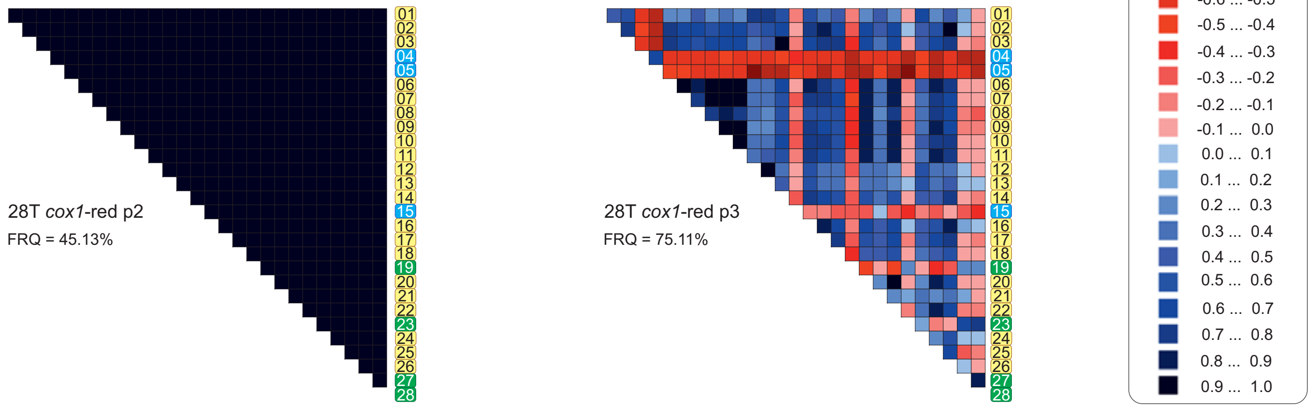

Supplementary Figure S25. AliGROOVE matrices and phylogenetic signal for *cox1* and *cox1*-red multiple alignments. p123 (positions 1-3 of the codons), p12 (positions 1-2 of the codons), p2 (positions 2 of the codons) and p3 (positions 3 of the codons), codon position/s included in the analysed multiple alignment. FRQ, percentage of **F**ully-**R**esolved **Q**uartets. AliGROOVE calculates a pairwise mean similarity score between each pair of sequences (-1 ≤ range ≤ 1), and returns a coloured matrix. In the matrix, each pairwise comparison is represented by a coloured square, which varies from deep blue (i.e. non-random similarity, +1) to deep-brown (i.e. full random similarity, deep-brown coloured; -1). A red square indicates that heterogeneous aligned positions dominate between the two sequences, while a blue square indicates that the opposite is true.

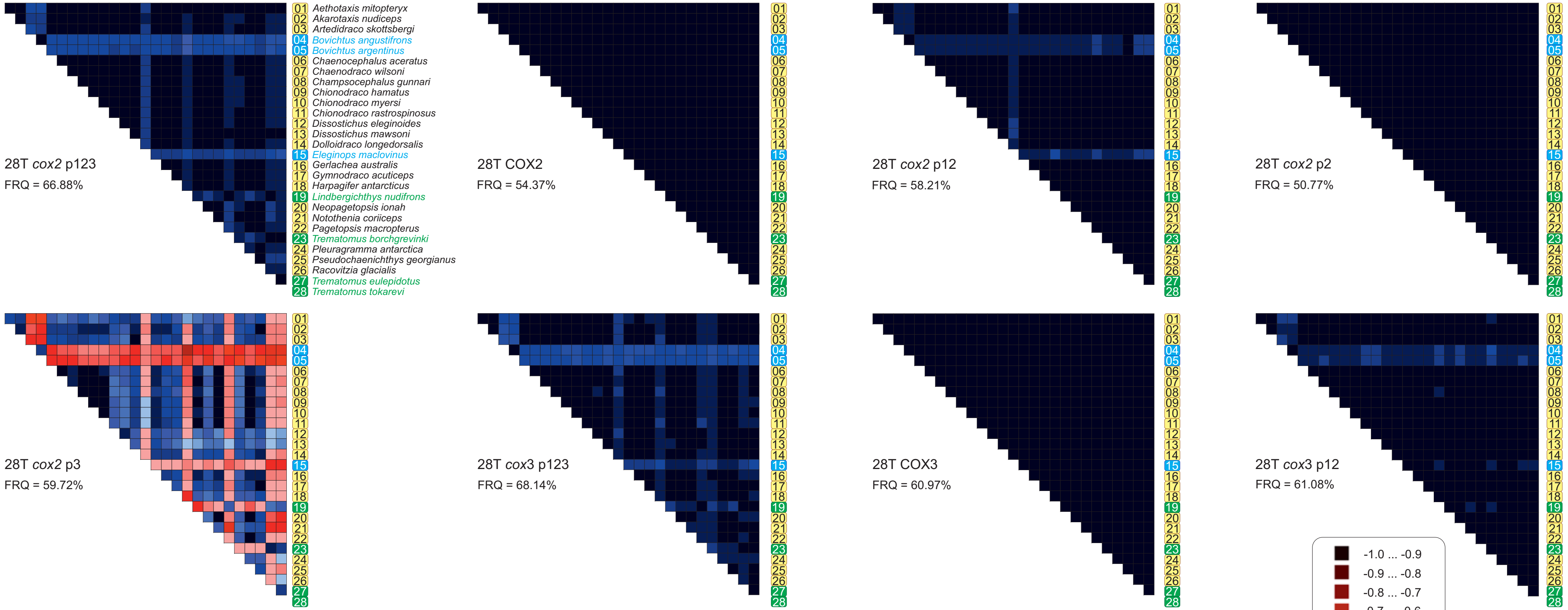

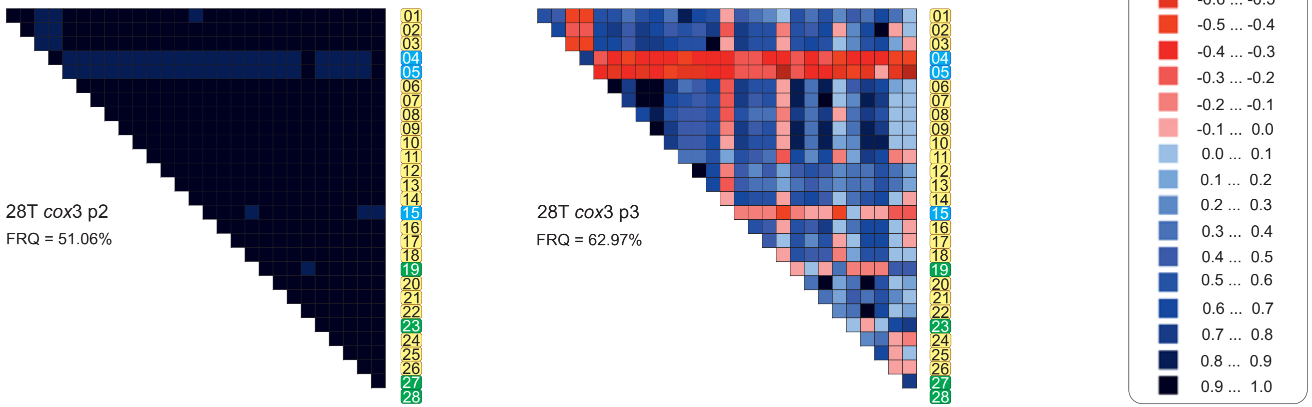

Supplementary Figure S26. AliGROOVE matrices and phylogenetic signal for *cox2* and *cox3* multiple alignments. p123 (positions 1-3 of the codons), p12 (positions 1-2 of the codons), p2 (positions 2 of the codons) and p3 (positions 3 of the codons), codon position/s included in the analysed multiple alignment. FRQ, percentage of **F**ully-**R**esolved **Q**uartets. AliGROOVE calculates a pairwise mean similarity score between each pair of sequences (-1 ≤ range ≤ 1), and returns a coloured matrix. In the matrix, each pairwise comparison is represented by a coloured square, which varies from deep blue (i.e. non-random similarity, +1) to deep-brown (i.e. full random similarity, deep-brown coloured; -1). A red square indicates that heterogeneous aligned positions dominate between the two sequences, while a blue square indicates that the opposite is true.

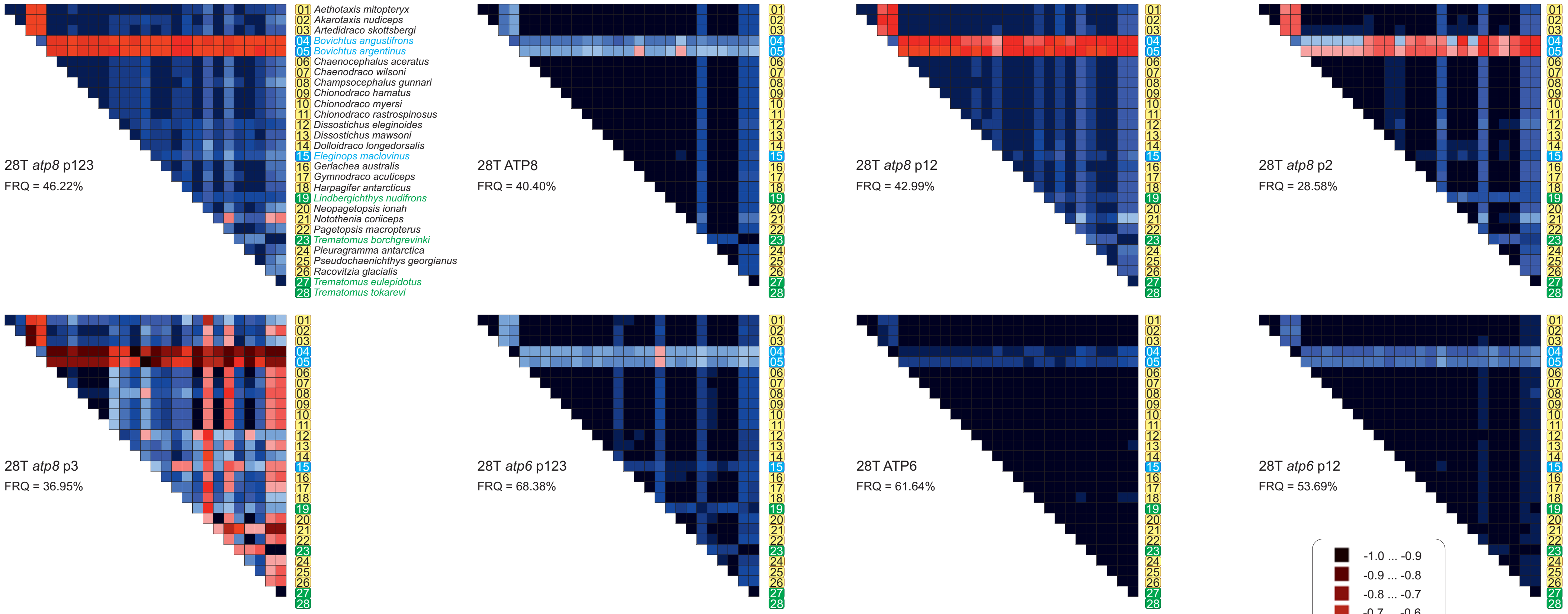

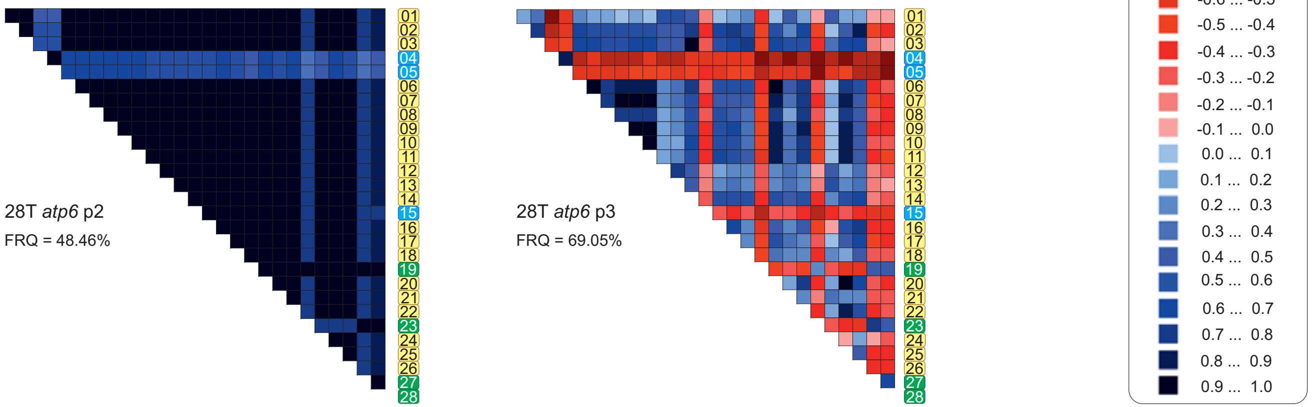

Supplementary Figure S27. AliGROOVE matrices and phylogenetic signal for *atp8* and *atp6* multiple alignments. p123 (positions 1-3 of the codons), p12 (positions 1-2 of the codons), p2 (positions 2 of the codons) and p3 (positions 3 of the codons), codon position/s included in the analysed multiple alignment. FRQ, percentage of **F**ully-**R**esolved **Q**uartets. AliGROOVE calculates a pairwise mean similarity score between each pair of sequences (-1 ≤ range ≤ 1), and returns a coloured matrix. In the matrix, each pairwise comparison is represented by a coloured square, which varies from deep blue (i.e. non-random similarity, +1) to deep-brown (i.e. full random similarity, deep-brown coloured; -1). A red square indicates that heterogeneous aligned positions dominate between the two sequences, while a blue square indicates that the opposite is true.

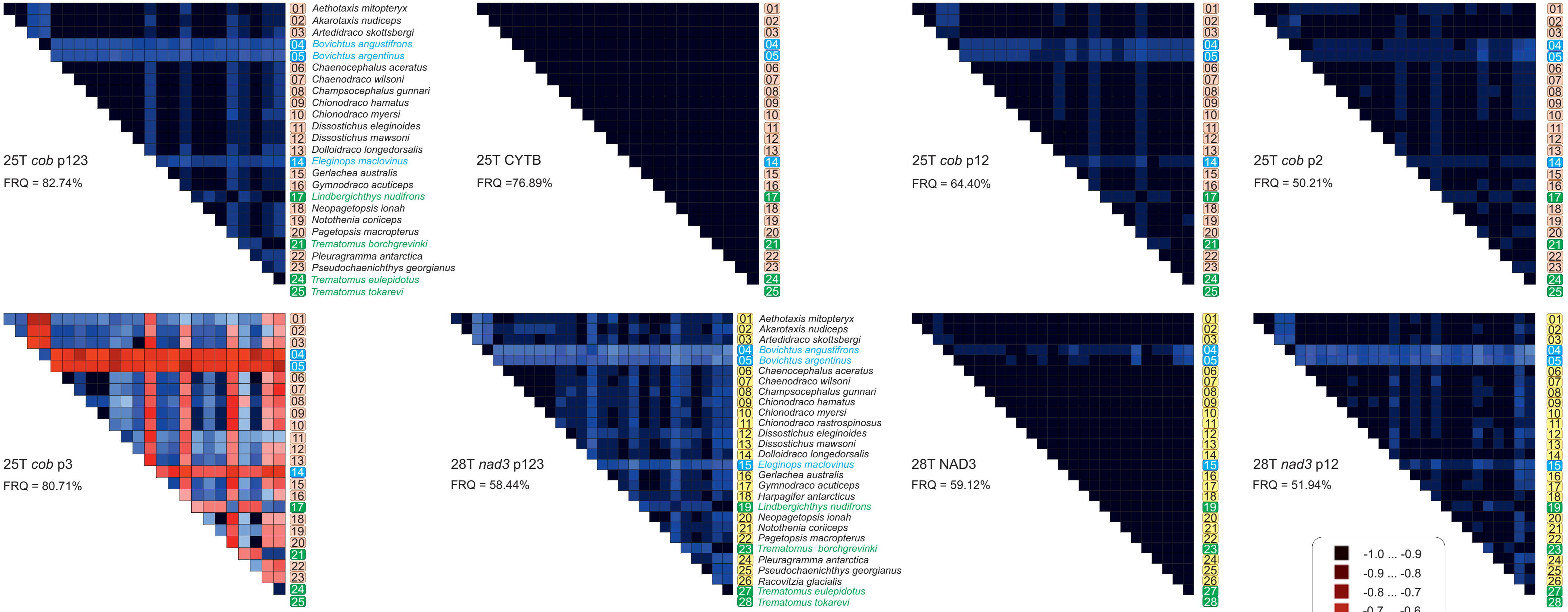

Supplementary Figure S28. AliGROOVE matrices and phylogenetic signal for *cob* and *nad3* multiple alignments. p123 (positions 1-3 of the codons), p12 (positions 1-2 of the codons), p2 (positions 2 of the codons) and p3 (positions 3 of the codons), codon position/s included in the analysed multiple alignment. FRQ, percentage of **F**ully-**R**esolved **Q**uartets. AliGROOVE calculates a pairwise mean similarity score between each pair of sequences (-1 ≤ range ≤ 1), and returns a coloured matrix. In the matrix, each pairwise comparison is represented by a coloured square, which varies from deep blue (i.e. non-random similarity, +1) to deep-brown (i.e. full random similarity, deep-brown coloured; -1). A red square indicates that heterogeneous aligned positions dominate between the two sequences, while a blue square indicates that the opposite is true.

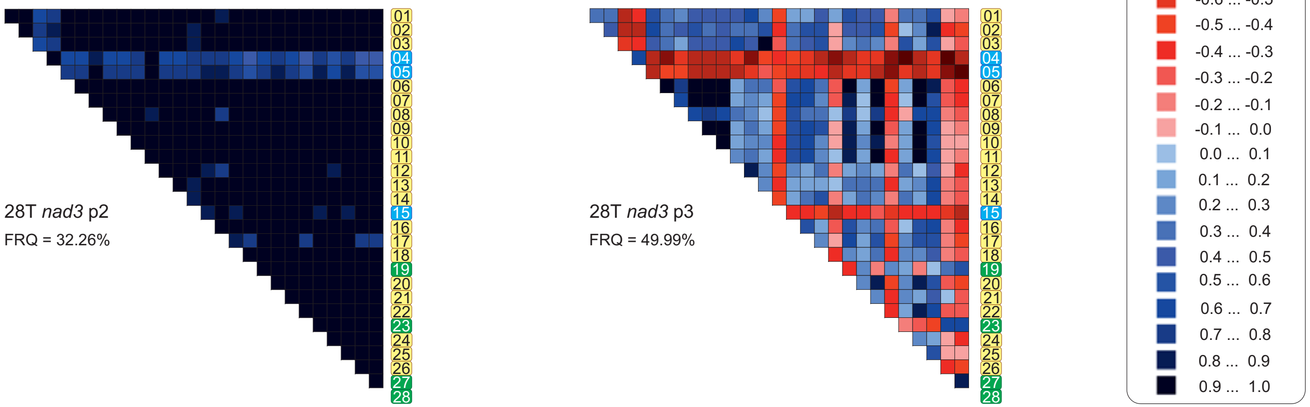

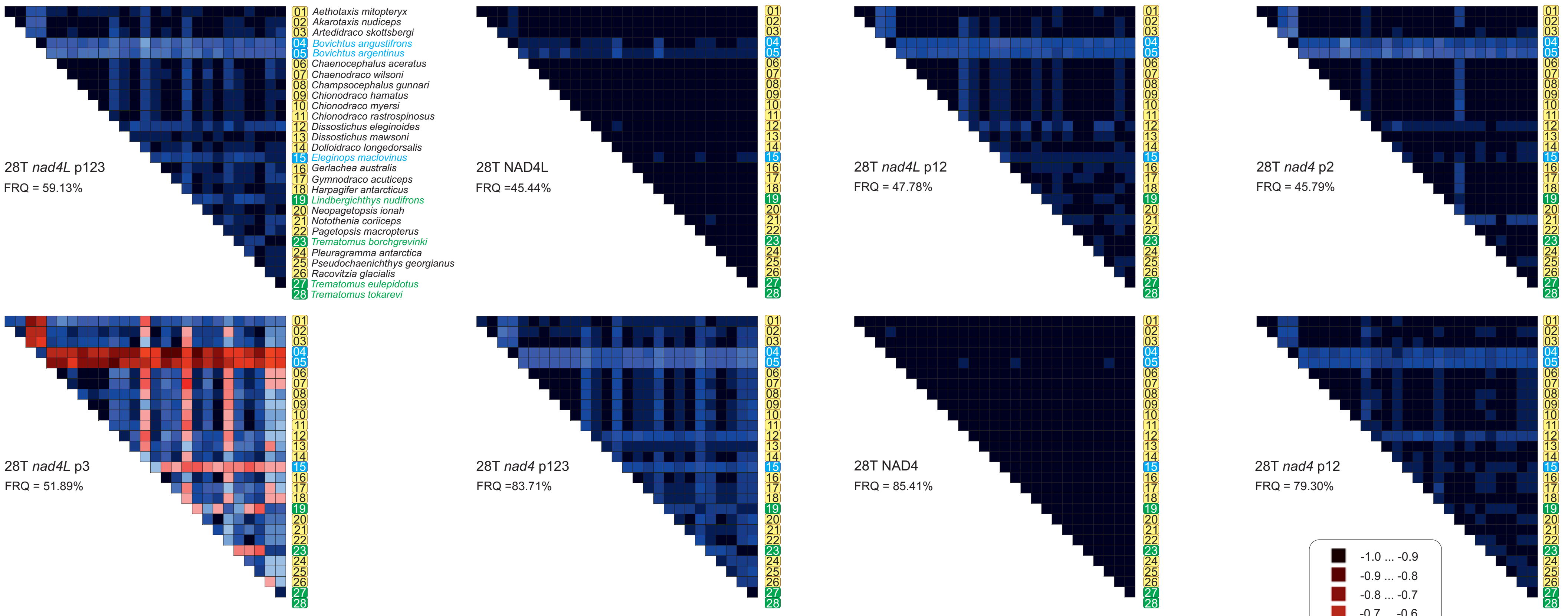

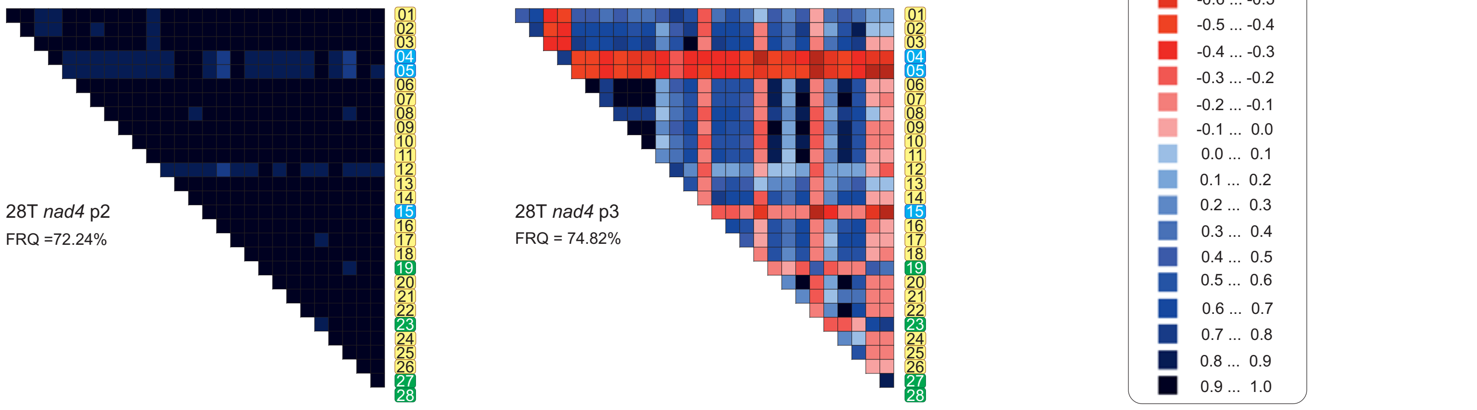

Supplementary Figure S29. AliGROOVE matrices and phylogenetic signal for *nad4L* and *nad4* multiple alignments. p123 (positions 1-3 of the codons), p12 (positions 1-2 of the codons), p2 (positions 2 of the codons) and p3 (positions 3 of the codons), codon position/s included in the analysed multiple alignment. FRQ, percentage of **F**ully-**R**esolved **Q**uartets. AliGROOVE calculates a pairwise mean similarity score between each pair of sequences (-1 ≤ range ≤ 1), and returns a coloured matrix. In the matrix, each pairwise comparison is represented by a coloured square, which varies from deep blue (i.e. non-random similarity, +1) to deep-brown (i.e. full random similarity, deep-brown coloured; -1). A red square indicates that heterogeneous aligned positions dominate between the two sequences, while a blue square indicates that the opposite is true.

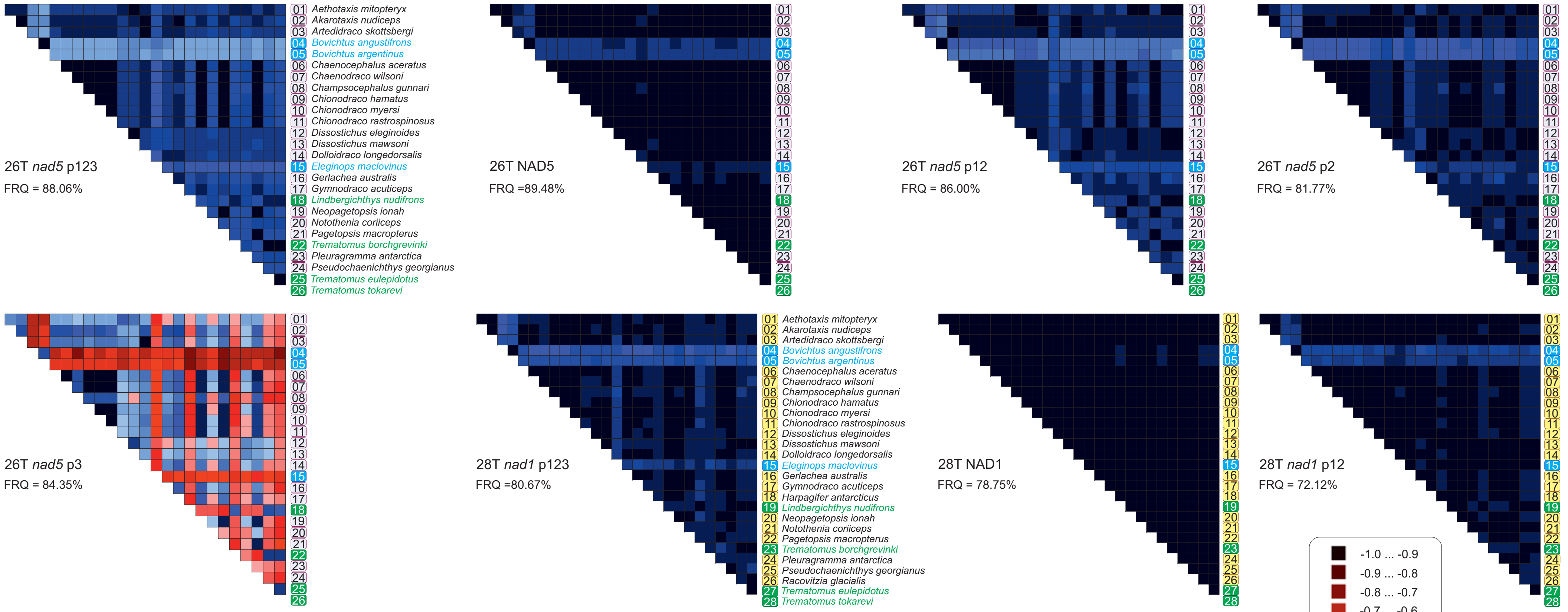

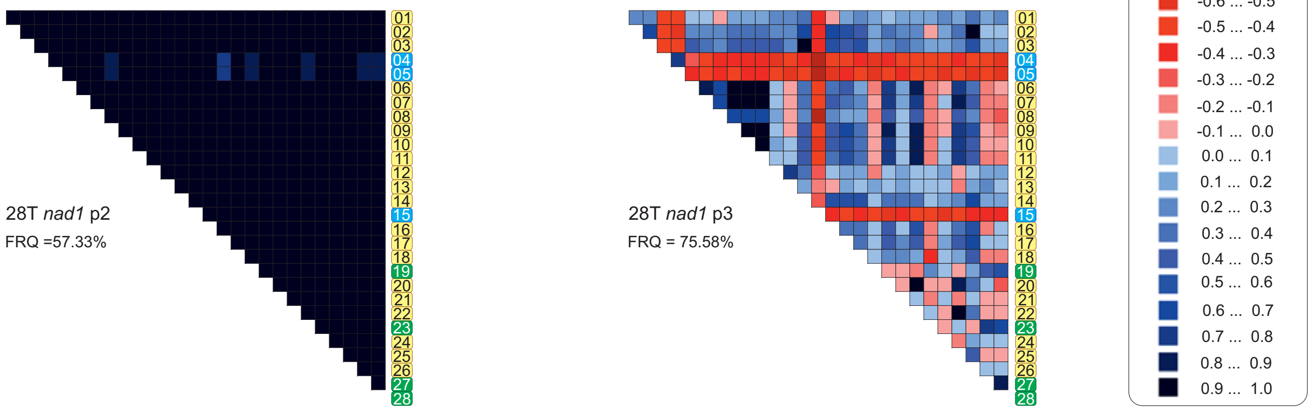

Supplementary Figure S30. AliGROOVE matrices and phylogenetic signal for *nad5* and *nad1* multiple alignments. p123 (positions 1-3 of the codons), p12 (positions 1-2 of the codons), p2 (positions 2 of the codons) and p3 (positions 3 of the codons), codon position/s included in the analysed multiple alignment. FRQ, percentage of **F**ully-**R**esolved **Q**uartets. AliGROOVE calculates a pairwise mean similarity score between each pair of sequences (-1 ≤ range ≤ 1), and returns a coloured matrix. In the matrix, each pairwise comparison is represented by a coloured square, which varies from deep blue (i.e. non-random similarity, +1) to deep-brown (i.e. full random similarity, deep-brown coloured; -1). A red square indicates that heterogeneous aligned positions dominate between the two sequences, while a blue square indicates that the opposite is true.

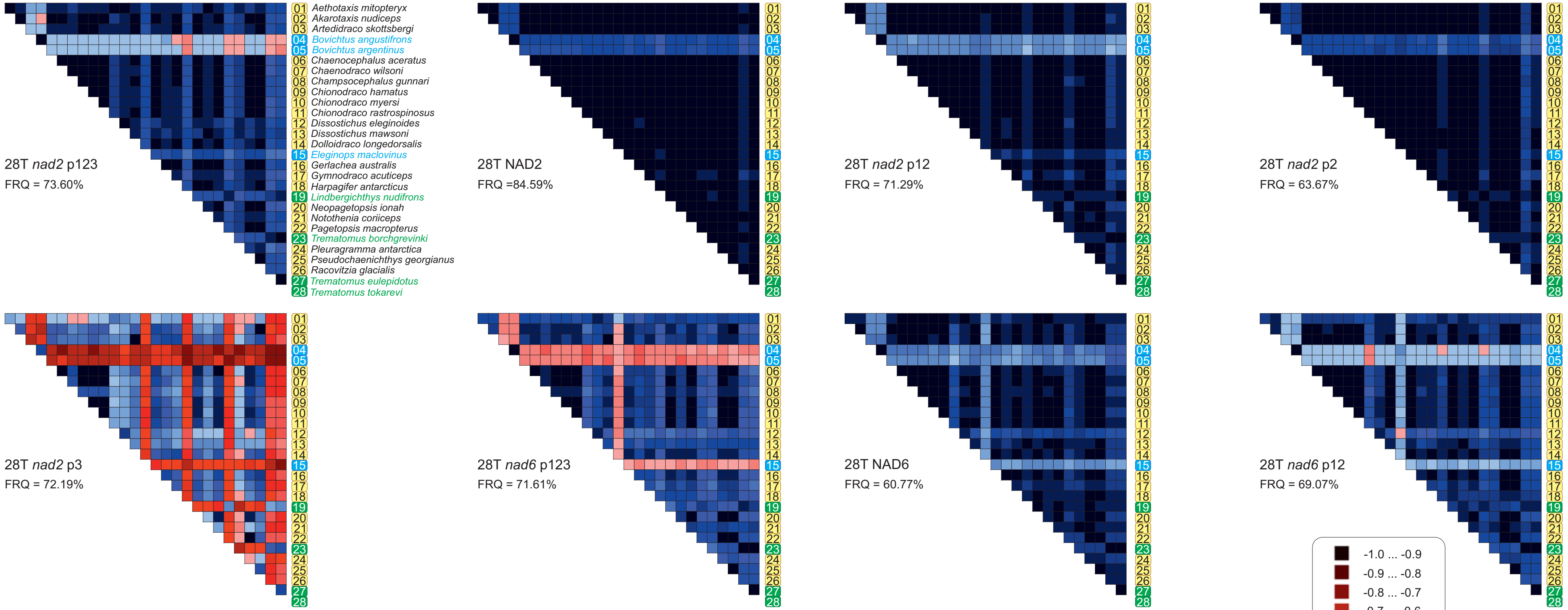

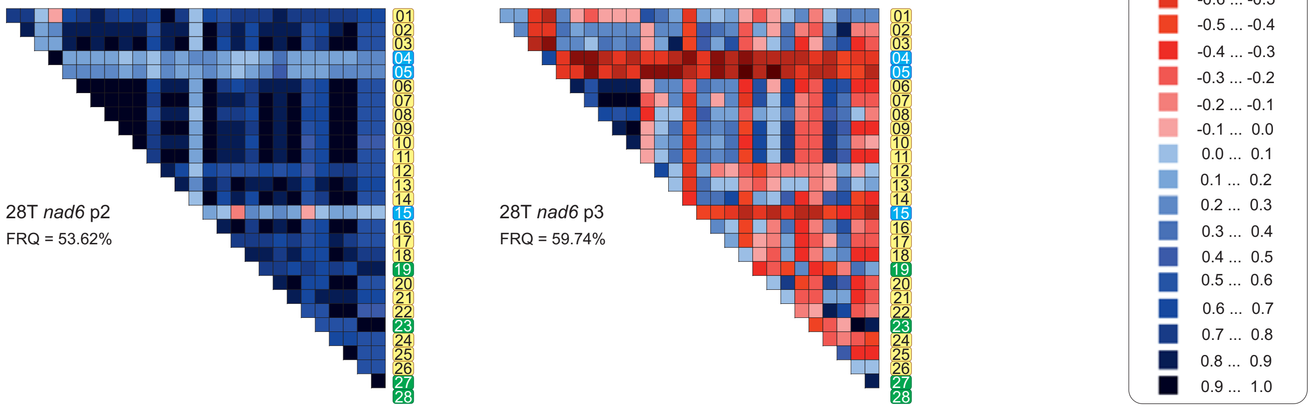

Supplementary Figure S31. AliGROOVE matrices and phylogenetic signal for *nad2* and *nad6* multiple alignments. p123 (positions 1-3 of the codons), p12 (positions 1-2 of the codons), p2 (positions 2 of the codons) and p3 (positions 3 of the codons), codon position/s included in the analysed multiple alignment. FRQ, percentage of **F**ully-**R**esolved **Q**uartets. AliGROOVE calculates a pairwise mean similarity score between each pair of sequences (-1 ≤ range ≤ 1), and returns a coloured matrix. In the matrix, each pairwise comparison is represented by a coloured square, which varies from deep blue (i.e. non-random similarity, +1) to deep-brown (i.e. full random similarity, deep-brown coloured; -1). A red square indicates that heterogeneous aligned positions dominate between the two sequences, while a blue square indicates that the opposite is true.

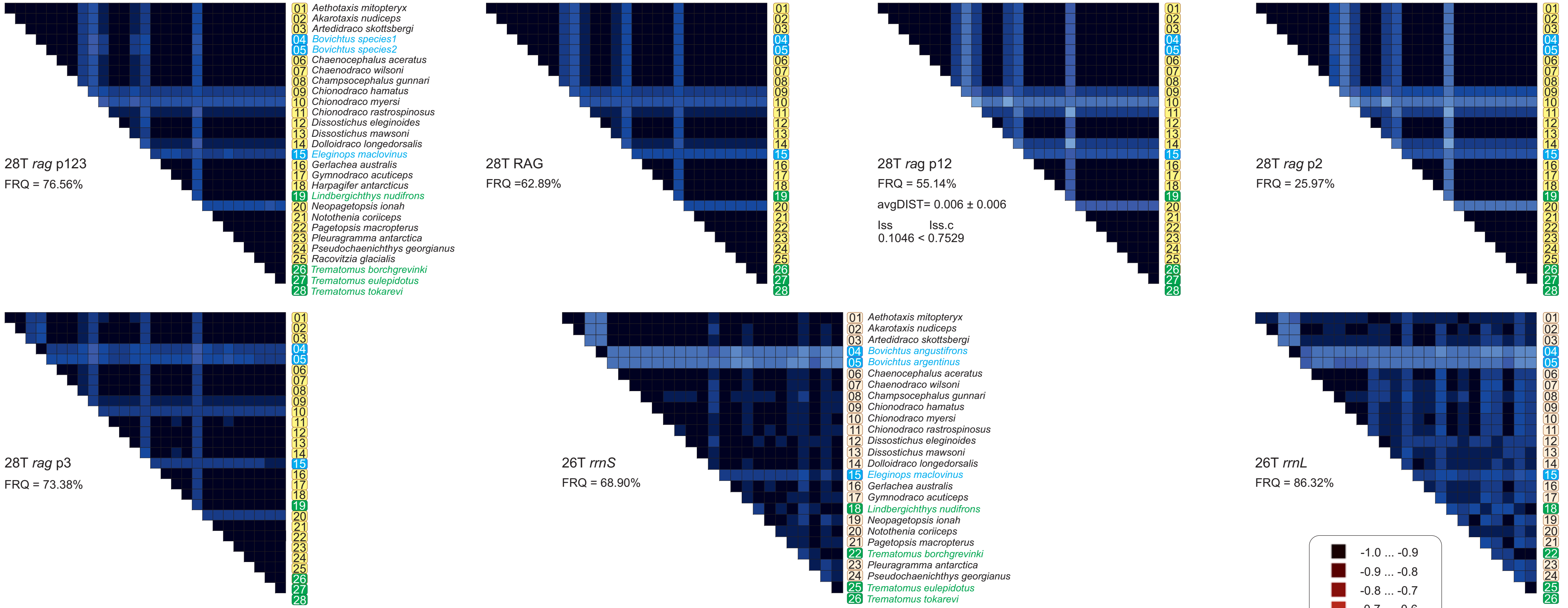

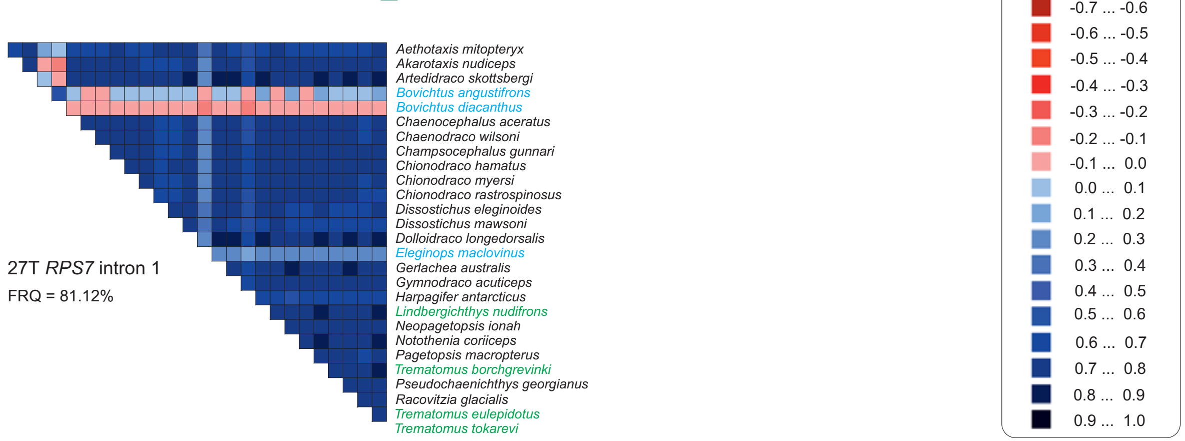

Supplementary Figure S32. AliGROOVE matrices and phylogenetic signal for *RAG*, *rrnS*, *rrnL* and *RPS7 intron 1* multiple alignments. p123 (positions 1-3 of the codons), p12 (positions 1-2 of the codons), p2 (positions 2 of the codons) and p3 (positions 3 of the codons), codon position/s included in the analysed multiple alignment. FRQ, percentage of **F**ully-**R**esolved **Q**uartets. AliGROOVE calculates a pairwise mean similarity score between each pair of sequences (-1 ≤ range ≤ 1), and returns a coloured matrix. In the matrix, each pairwise comparison is represented by a coloured square, which varies from deep blue (i.e. non-random similarity, +1) to deep-brown (i.e. full random similarity, deep-brown coloured; -1). A red square indicates that heterogeneous aligned positions dominate between the two sequences, while a blue square indicates that the opposite is true.

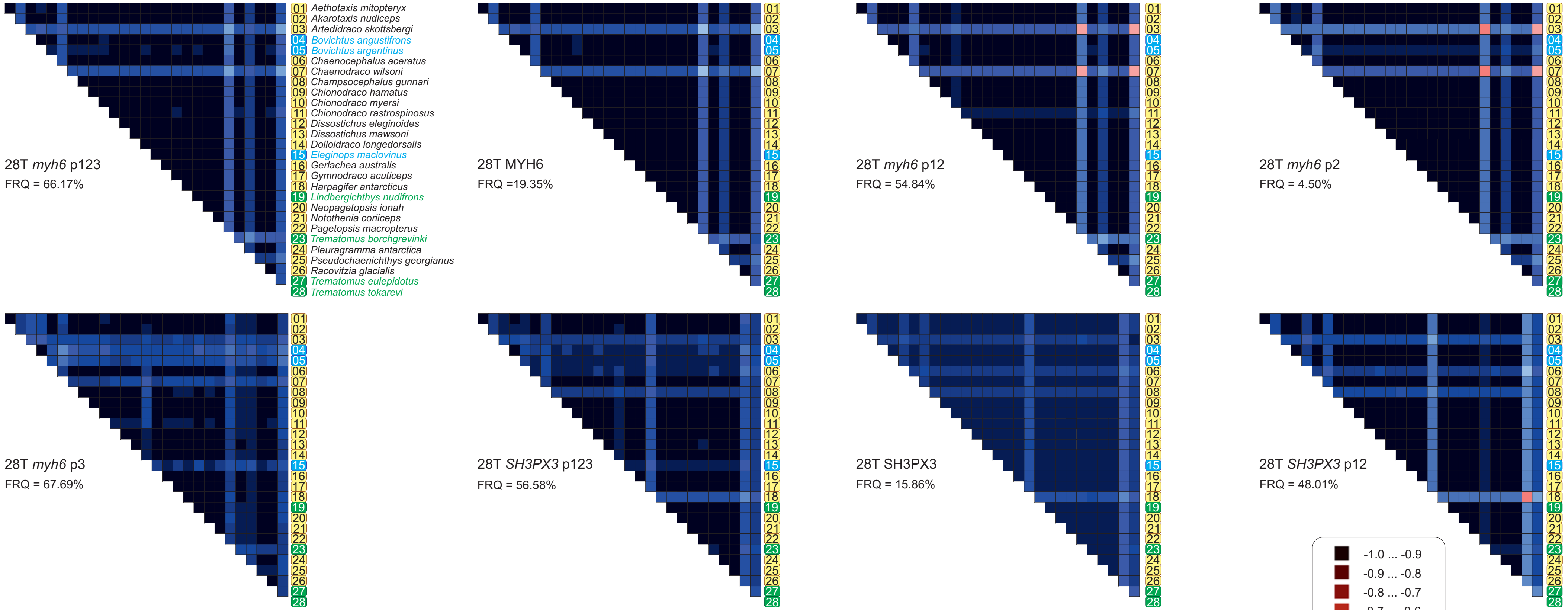

Supplementary Figure S33. AliGROOVE matrices and phylogenetic signal for *myh6* and *SH3PX3* multiple alignments. p123 (positions 1-3 of the codons), p12 (positions 1-2 of the codons), p2 (positions 2 of the codons) and p3 (positions 3 of the codons), codon position/s included in the analysed multiple alignment. FRQ, percentage of **F**ully-**R**esolved **Q**uartets. AliGROOVE calculates a pairwise mean similarity score between each pair of sequences (-1 ≤ range ≤ 1), and returns a coloured matrix. In the matrix, each pairwise comparison is represented by a coloured square, which varies from deep blue (i.e. non-random similarity, +1) to deep-brown (i.e. full random similarity, deep-brown coloured; -1). A red square indicates that heterogeneous aligned positions dominate between the two sequences, while a blue square indicates that the opposite is true.

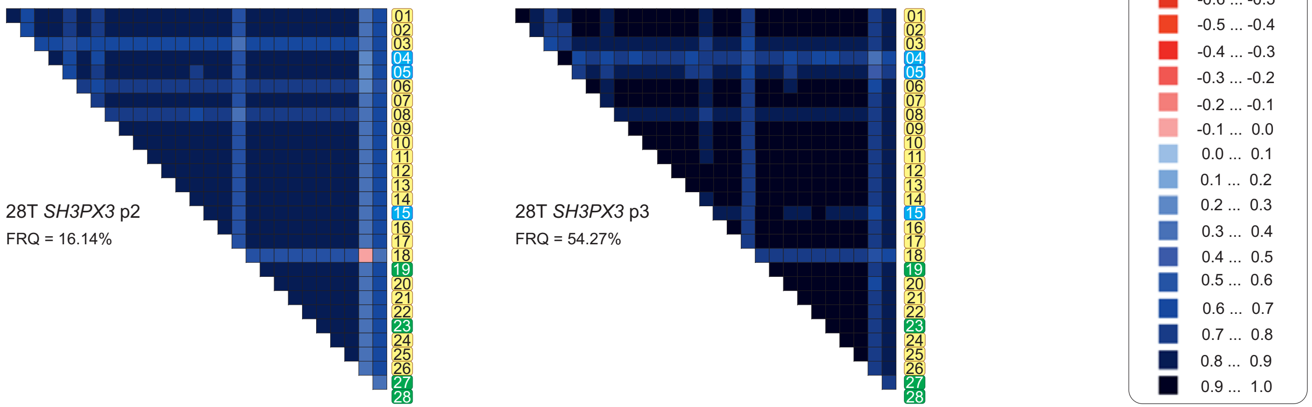

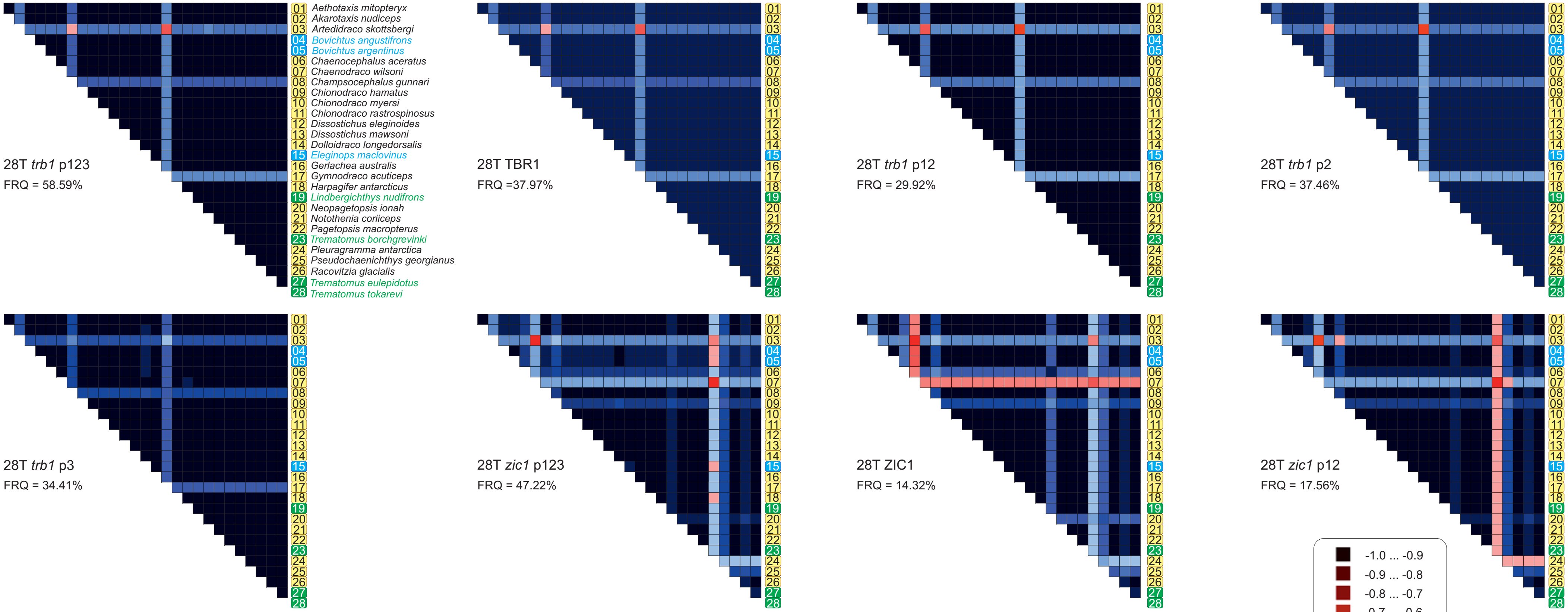

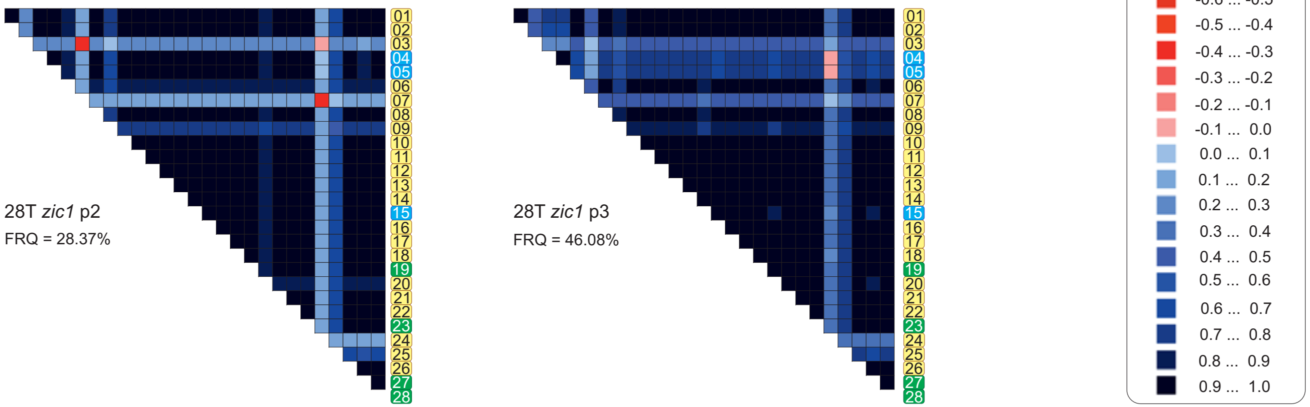

Supplementary Figure S34. AliGROOVE matrices and phylogenetic signal for *tbr1* and *zic1* multiple alignments. p123 (positions 1-3 of the codons), p12 (positions 1-2 of the codons), p2 (positions 2 of the codons) and p3 (positions 3 of the codons), codon position/s included in the analysed multiple alignment. FRQ, percentage of **F**ully-**R**esolved **Q**uartets. AliGROOVE calculates a pairwise mean similarity score between each pair of sequences (-1 ≤ range ≤ 1), and returns a coloured matrix. In the matrix, each pairwise comparison is represented by a coloured square, which varies from deep blue (i.e. non-random similarity, +1) to deep-brown (i.e. full random similarity, deep-brown coloured; -1). A red square indicates that heterogeneous aligned positions dominate between the two sequences, while a blue square indicates that the opposite is true.

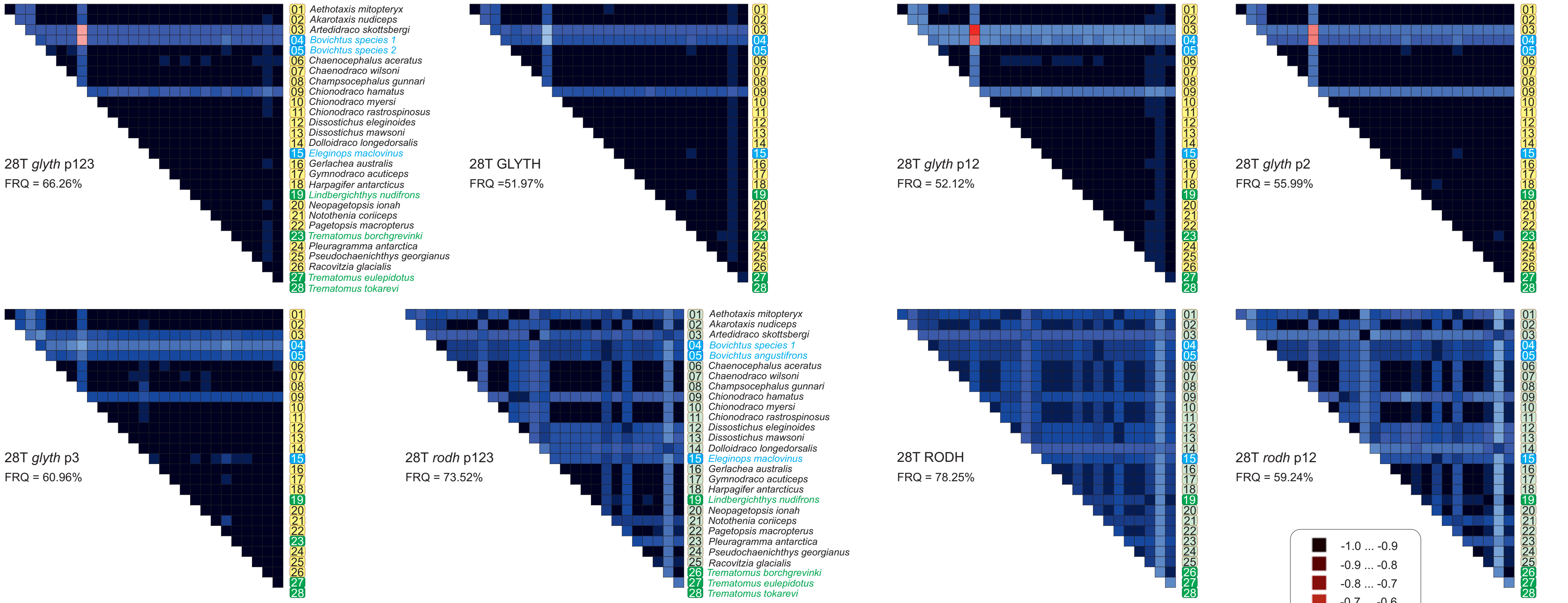

Supplementary Figure S35. AliGROOVE matrices and phylogenetic signal for *glyth* and *rodh* multiple alignments. p123 (positions 1-3 of the codons), p12 (positions 1-2 of the codons), p2 (positions 2 of the codons) and p3 (positions 3 of the codons), codon position/s included in the analysed multiple alignment. FRQ, percentage of **F**ully-**R**esolved **Q**uartets. AliGROOVE calculates a pairwise mean similarity score between each pair of sequences (-1 ≤ range ≤ 1), and returns a coloured matrix. In the matrix, each pairwise comparison is represented by a coloured square, which varies from deep blue (i.e. non-random similarity, +1) to deep-brown (i.e. full random similarity, deep-brown coloured; -1). A red square indicates that heterogeneous aligned positions dominate between the two sequences, while a blue square indicates that the opposite is true.

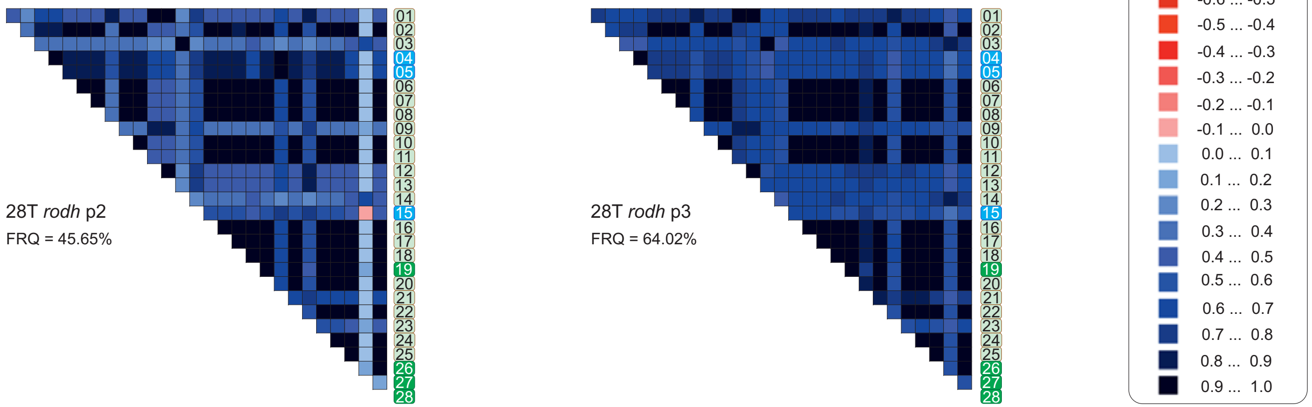

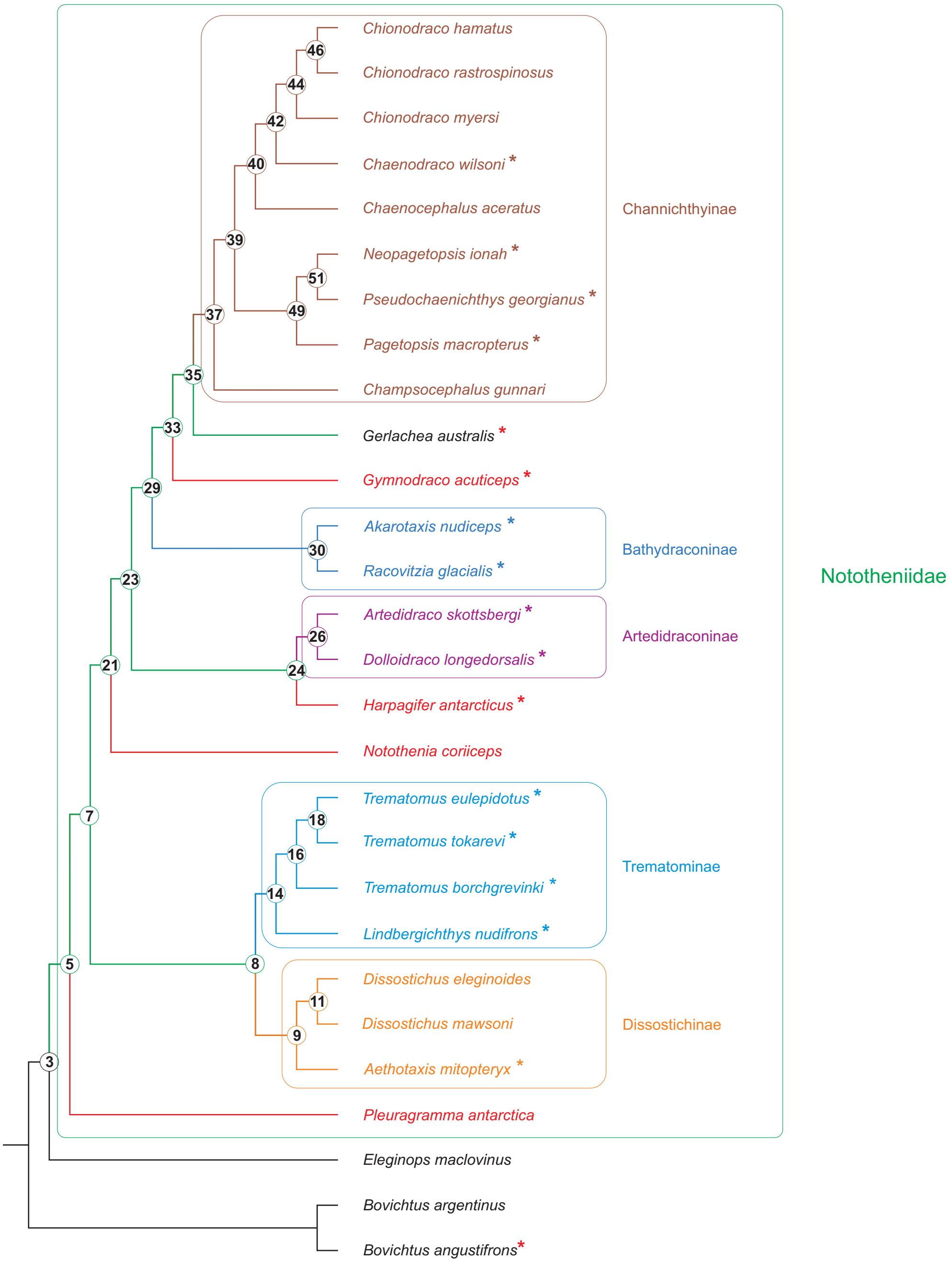

Supplementary Figure S36. Reference tree topology used in the aBSREL and RELAX analyses. Key internal nodes are marked to allow a rapid identification of the reference branches vs the test branches in the analyses. The coloured boxes identify different groups of test braches. For details, see the Type of selection acting on mitochondrial protein-coding genes: a phylogeny-based strategy paragraph in Supplementary Results and Discussion and Materials and Methods sections.

Supplementary Table S1. List of taxa *de novo* sequenced in this study and methodological and collection details. The table reports: the subfamily and species names of all 17 newly sequenced mitochondrial genomes, the geographic site of collection and the sampling cruise (locality (cruise)), the year of sampling, the institute where samples are stored or were stored (repository), the method for DNA extraction, and the sequencing technology. The classification of Notothenioidei follows Duhamel et al. (2014). The taxonomy of species follows WoRMS database (World Register of Marine Species, http://www.marinespecies.org/index.php; accessed 15-01-2021).

	Subfamily	Species	Locality (Cruise)	Year	Repository	DNA extraction	Sequencing
	**Bovicthidae**						
1	Bovichtidae	*Bovicthus angustifrons* Regan, 1913	Fossil Island, Tasmania, Australia		AWI	DNeasy Blood and Tissue Kit (Qiagen, Germany)	Illumina
	**Nototheniidae**						
2	Dissostichinae	*Aethotaxis mitopteryx* DeWitt, 1962	Southern Ocean	2004	MNHN	CTAB (Winnepenninckx et al. 1993)	Long PCRs, Ion Torrent
3	Trematominae	*Lindbergichthys nudifrons*# (Lönnberg, 1905)	South Shetlands, Elephant Island (RV Polarstern, PS79)	2012	AWI	RNeasy Mini (Qiagen, Hilden, Germany)	Illumina
4	Trematominae	*Trematomus borchgrevinki* (Boulenger, 1902)	line fishing next to Dumont d'Urville Station	2013	MNHN	CTAB (Winnepenninckx et al. 1993)	Long PCRs, Ion Torrent
5	Trematominae	*Trematomus eulepidotus* Regan, 1914	Weddell Sea (RV Polarstern, PS82)	2014	AWI	RNeasy Mini (Qiagen, Hilden, Germany)	Illumina
6	Trematominae	*Trematomus tokarevi* Andriashev, 1978	South Shetlands, Elephant Island (RV Polarstern, PS79)	2012	UNIPD	ZR Genomic DNATissue Midiprep Kit (Zymo Research corp.)	Illumina
7	Artedidraconinae	*Artedidraco skottsbergi* Lönnberg, 1905	South Shetlands, Elephant Island (RV Polarstern, PS79)	2012	UNIPD	ZR Genomic DNATissue Midiprep Kit (Zymo Research corp.)	Illumina
8	Artedidraconinae	*Dolloidraco longedorsalis* Roule, 1913	South Shetlands, Elephant Island (RV Polarstern, PS79)	2012	UNIPD	ZR Genomic DNATissue Midiprep Kit (Zymo Research corp.)	Illumina
9	Harpagiferinae	*Harpagifer antarcticus* Nybelin, 1947	South Shetlands, Elephant Island (RV Polarstern, PS79)	2012	UNIPD	Invisorb DNA extraction kit (Invitec).	Sanger
10	Bathydraconinae	*Akarotaxis nudiceps* (Waite, 1916)	South Shetlands, Elephant Island (RV Polarstern, PS79)	2012	UNIPD	ZR Genomic DNATissue Midiprep Kit (Zymo Research corp.)	Illumina
11	Bathydraconinae	*Racovitzia glacialis* Dollo, 1900	South Shetlands, Elephant Island (RV Polarstern, PS79)	2012	UNIPD	Invisorb DNA extraction kit (Invitec).	Sanger
12	Cygnodraconinae	*Gerlachea australis* Dollo, 1900	South Shetlands, Elephant Island (RV Polarstern, PS79)	2012	UNIPD	ZR Genomic DNATissue Midiprep Kit (Zymo Research corp.)	Illumina
13	Gymnodraconinae	*Gymnodraco acuticeps* Boulenger, 1902	South Shetlands, Elephant Island (RV Polarstern, PS79)	2012	UNIPD	ZR Genomic DNATissue Midiprep Kit (Zymo Research corp.)	Illumina
14	Channichthyinae	*Chaenodraco wilsoni* Regan, 1914	South Shetlands, Elephant Island (RV Polarstern, PS79)	2012	UNIPD	ZR Genomic DNATissue Midiprep Kit (Zymo Research corp.)	Illumina
15	Channichthyinae	*Neopagetopsis ionah* Nybelin, 1947	South Shetlands, Elephant Island (RV Polarstern, PS79)	2012	UNIPD	ZR Genomic DNATissue Midiprep Kit (Zymo Research corp.)	Illumina
16	Channichthyinae	*Pagetopsis macropterus* (Boulenger, 1907)	South Shetlands, Elephant Island (RV Polarstern, PS79)	2012	UNIPD	ZR Genomic DNATissue Midiprep Kit (Zymo Research corp.)	Illumina
17	Channichthyinae	*Pseudochaenichthys georgianus* Norman, 1937	South Shetlands, Elephant Island (RV Polarstern, PS79)	2012	UNIPD	ZR Genomic DNATissue Midiprep Kit (Zymo Research corp.)	Illumina

MNHN, Muséum national d'Histoire naturelle (Paris, France); AWI, Alfred Wegener Institute Helmholtz Centre for Polar and Marine Research (Bremerhaven, Germany); UNIPD, University of Padova (Padova, Italy). # Peninsular *Lindbergichthys nudifrons* species, *sensu* Dornburg et al. (2016).

References to Supplementary Table S1

Dornburg A, Federman S, Eytan RI, Near TJ. 2016. Cryptic species diversity in sub-Antarctic islands: A case study of *Lepidonotothen*. *Mol Phylogenet Evol.* 104:32-43.

Duhamel G, Hulley PA, Causse R, Koubbi P, Vacchi M, Pruvost P, Vigetta S, Irisson JO, Mormède S, Belchier M, et al. 2014. Biogeographic patterns of fish. In De Broyer C, Koubbi P, Griffiths H, Raymond B, d'Udekem d'Acoz C, Van de Putte A, Danis B, David B, Grant S, Gutt J, et al., editors. Biogeographic Atlas of the Southern Ocean. Cambridge: Scientific Committee on Antarctic Research. p. 328-498.

Winnepenninckx B, Backeljau T, De Wachter R. 1993. Extraction of high molecular weight DNA from molluscs. *Trends Genet.* **9**:407.

Supplementary Table S2. List of taxa considered in the analyses and accession numbers for mitochondrial sequences. The table reports: the subfamily and species names, the GenBank accession number of the mitochondrial genome, the GenBank accession number of the Control Region (CoRe), the degree of sequence completeness (coverage), the acronym of the gene order arrangement (GO, see main text) and the reference citing the genome (Ref). The classification of Notothenioidei follows Duhamel et al. (2014). The taxonomy of species follows WoRMS database (World Register of Marine Species, http://www.marinespecies.org/index.php; accessed 15-01-2021).

	Subfamily	Species	GenBank	CoRe	coverage	GO	Ref
**Bovichtidae**							
**1**	Bovichtidae	*Bovichtus angustifrons* Regan, 1913	**MT559884**		**complete**	**VertGO**	**(2)**
**2**	Bovichtidae	*Bovichtus argentinus* MacDonagh, 1931	AP006020		**complete**	**VertGO**	**(3)**
**Eleginopsidae**							
**3**	Eleginopsidae	*Eleginops maclovinus* (Cuvier, 1830)	KY038381		**complete**	**VertGO**	**(4)**
**Nototheniidae**							
**4**	Pleuragramminae	*Pleuragramma antarctica* Boulenger, 1902	JF933905		**complete**	**Noto1GO**	**(5)**
**5**	Dissostichinae	*Aethotaxis mitopteryx* DeWitt, 1962	**MT232658**		**complete**	**Noto1GO**	**(2)**
**6**	Dissostichinae	*Dissostichus eleginoides* Smitt, 1898	AB723627		**complete**	**DissoGO**	**(6)**
**7**	Dissostichinae	*Dissostichus mawsoni* Norman, 1937	LC138011		**complete**	**DissoGO**	**(7)**
**8**	Trematominae	*Lindbergichthys nudifrons* (Lönnberg, 1905)	**MT559890**		**complete**	**TremaGO**	**(2)**
**9**	Trematominae	*Trematomus borchgrevinki* (Boulenger, 1902)	**MT232659**		**complete**	**TremaGO**	**(2)**
**10**	Trematominae	*Trematomus eulepidotus* Regan, 1914	**MT559895**		**complete**	**TremaGO**	**(2)**
**11**	Trematominae	*Trematomus tokarevi* Andriashev, 1978	**MT559896**		**complete**	**TremaGO**	**(2)**
**12**	Nototheniinae	*Notothenia coriiceps* Richardson, 1844	JF933906		**complete**	**Noto2GO**	**(8)**
**13**	Artedidraconinae	*Artedidraco skottsbergi* Lönnberg, 1905	**MT559883**		**complete**	**Noto3GO**	**(2)**
**14**	Artedidraconinae	*Dolloidraco longedorsalis* Roule, 1913	**MT559886**		**complete**	**Noto3GO**	**(2)**
**15**	Harpagiferinae	*Harpagifer antarcticus* Nybelin, 1947	**MT559889**	GU214222	**partial**	**Noto2GO**	**(2, 9)**
**16**	Bathydraconinae	*Akarotaxis nudiceps* (Waite, 1916)	**MT559882**		**complete**	**Noto3GO**	**(2)**
**17**	Bathydraconinae	*Racovitzia glacialis* Dollo, 1900	**MT559894**	GU214226	**partial**	**RacoGO**	**(2, 9)**
**18**	Cygnodraconinae	*Gerlachea australis* Dollo, 1900	**MT559887**		**complete**	**Noto3GO**	**(2)**
**19**	Gymnodraconinae	*Gymnodraco acuticeps* Boulenger, 1902	**MT559888**		**complete**	**GymnGO**	**(2)**
**20**	Channichthyinae	*Champsocephalus gunnari* Lönnberg, 1905	GU217678		**complete**	**ChamGO**	**(10)**
**21**	Channichthyinae	*Chaenocephalus aceratus* (Lönnberg, 1906)	JF933907		**complete**	**Noto3GO**	**(11)**
**22**	Channichthyinae	*Chaenodraco wilsoni* Regan, 1914	**MT559885**		**complete**	**Noto3GO**	**(2)**
**23**	Channichthyinae	*Chionodraco hamatus* (Lönnberg, 1905)	KT921282		**complete**	**Noto3GO**	**(12)**
**24**	Channichthyinae	*Chionodraco myersi* DeWitt & Tyler, 1960	DQ526430	GU214228	**partial**	**Noto3GO**	**(9, 13)**
**25**	Channichthyinae	*Chionodraco rastrospinosus* DeWitt & Hureau, 1979	DQ526431	GU214229	**partial**	**Noto3GO**	**(9, 13)**
**26**	Channichthyinae	*Neopagetopsis ionah* Nybelin, 1947	**MT559891**		**complete**	**Noto3GO**	**(2)**
**27**	Channichthyinae	*Pagetopsis macropterus* (Boulenger, 1907)	**MT559892**		**complete**	**Noto3GO**	**(2)**
**28**	Channichthyinae	*Pseudochaenichthys georgianus* Norman, 1937	**MT559893**		**complete**	**Noto3GO**	**(2)**
**Sequences excluded from the analysis (see Supplementary Information for details)**							
	Trematominae	*Trematomus bernacchii* Boulenger, 1902	KU166863.1				**(14)**
	Trematominae	*Trematomus borchgrevinki* (Boulenger, 1902)	KU951144				**(15)**
	Trematominae	*Trematomus borchgrevinki* (Boulenger, 1902)	KX025131				**(16)**
	Trematominae	*Trematomus pennellii* Regan, 1914	MK007073		**partial**		**(17)**
	Cygnodraconinae	*Parachaenichthys charcoti* (Vaillant, 1906)	KP300644.1				**(18)**

References to Supplementary Table S2

1. Duhamel G, Hulley PA, Causse R, Koubbi P, Vacchi M, Pruvost P, Vigetta S, Irisson JO, Mormède S, Belchier M, et al. 2014. Biogeographic patterns of fish. In De Broyer C, Koubbi P, Griffiths H, Raymond B, d'Udekem d'Acoz C, Van de Putte A, Danis B, David B, Grant S, Gutt J, et al., editors. Biogeographic Atlas of the Southern Ocean. Cambridge: Scientific Committee on Antarctic Research. p. 328-498.
2. **This study**.
3. Satoh TP, Miya M, Mabuchi K, Nishida M. 2016. Structure and variation of the mitochondrial genome of fishes. BMC Genomics 17:719.
4. Lee-Estevez M, Larama G, Figueroa E, Ulloa-Rodríguez P, Díaz R, Valdebenito I, Farías JG. 2019. Complete mitochondrial genome sequence of Patagonian blenny, *Eleginops maclovinus* (Perciformes: Eleginopidae) with phylogenetic consideration. Conserv Genet Resour. 11:23-26.
5. Lee J, Lee H, Lee J, Choi J, Park H. 2015. Complete mitochondrial genome of the Antarctic silverfish, *Pleuragramma antarcticum* (Perciformes, Nototheniidae). Mitochondrial DNA 26:885-886.
6. Yanagimoto T, Nishida T. Unpublished.
7. Nagata K, Ota T. Unpublished.
8. Oh JS, Ahn DH, Lee J, Choi J, Chi YM, Park H. 2016. Complete mitochondrial genome of the Antarctic bullhead notothen, *Notothenia coriiceps* (Perciformes, Nototheniidae). Mitochondrial DNA A 27:1407-1408.
9. Zhuang X, Cheng CHC. 2010. ND6 gene "lost" and found: evolution of mitochondrial gene rearrangement in Antarctic notothenioids. *Mol Biol Evol*. 27:1391-1403.
10. Lin CY, Lin, WW, Kao, HW. 2012. The complete mitochondrial genome of the mackerel icefish, *Champsocephalus gunnari* (Actinopterygii: Channichthyidae), with reference to the evolution of mitochondrial genomes in Antarctic notothenioids. Zool J Lin Soc. 165:521-533.
11. Lee J, Lee H, Lee J, Jo J, Choi J, Park H. 2015. Complete mitochondrial genome of the Antarctic icefish, *Chaenocephalus aceratus* (Perciforms, Channichthyidae). Mitochondrial DNA 26:887-888.
12. Song W, Li L, Huang H, Meng Y, Jiang K, Zhang F, Chen X, Ma L. 2016. The complete mitochondrial genome of *Chionodraco hamatus* (Notothenioidei: Channichthyidae) with phylogenetic consideration. Mitochondrial DNA B 1:52-53.
13. Papetti C, Liò P, Rüber L, Patarnello T, Zardoya, R. 200. 7Antarctic fish mitochondrial genomes lack ND6 gene. J Mol Evol. 65:519-528.
14. Song W, Li L, Huang H, Zhao M, Jiang K, Zhang F, Zhao M, Chen X, Ma L. 2016. The complete mitochondrial genome sequence and gene organization of *Trematomus bernacchii* (Perciformes: Nototheniidae) with phylogenetic consideration. Mitochondrial DNA B 1:50-51.
15. Liu Y, Yang M, Zhang D, Chen L. Unpublished.
16. Zhao M, Song W, Ma L, Jiang KJ, Zhang FY. Unpublished.
17. Alam MJ, Kim JH, Andriyono S, Lee JH, Lee SR, Park H, Kim HW. 2019. Characterization of complete mitochondrial genome and gene organization of sharp-spined notothenia, *Trematomus pennellii* (Perciformes: Nototheniidae). Mitochondrial DNA B 4:648-649.
18. Oh JS, Kang S, Ahn DH, Kim MK, Lee H, Chi YM, Park H. 2016. Complete mitochondrial genome of the Antarctic dragonfish, *Parachaenichthys charcoti* (Notothenioidei: Bathydraconidae). Mitochondrial DNA A 27:3151-3152.

Supplementary Table S3. List of taxa considered in the analyses and accession numbers for nuclear sequences. *Glyt,* glycosyltransferase (*glyt*) gene; *myh6*, muscle myosin heavy chain 6 alpha (*myh6*) gene; *rag1*, recombination activating protein 1 (*RAG-1*) gene; *Rhod*, rhodopsin (*Rhod*) gene; *S7i,* ribosomal protein S7 (*RPS7*) gene, intron 1; *SH3PX3, SH3* and *PX* domain-containing 3-like protein (*SH3PX3*) gene; *tbr1*, T-box brain 1 (*tbr1*) gene; *zic1*, zic family member 1 (*zic1*) gene.

	**Species**	***glyt***	***myh6***	***rag1***	***Rhod***	***S7i***	***SH3PX3***	***tbr1***	***zic1***
**1**	*Bovichtus angustifrons* Regan, 1913*	KF139693	JN187019	Dornb2017	AY141299	JN186842	JN186937	JN186978	JN186801
**2**	*Bovichtus argentinus* MacDonagh, 1931*	KF139693	JN187020	KF141190	AY141299	JN186843	JN186938	JN186979	JN186802
**3**	*Eleginops maclovinus* (Cuvier, 1830)	KF139739	JN187023	KF141229	AY141303	JN186846	JN186941	JN186982	JN186805
**4**	*Pleuragramma antarctica* Boulenger, 1902	Dornb2017	JN187057	Dornb2017	DQ498795	***	JN186975	JN187016	JN186839
**5**	*Aethotaxis mitopteryx* DeWitt, 1962	Dornb2017	JN187038	Dornb2017	DQ498781	JN186862	JN186956	JN186997	JN186820
**6**	*Dissostichus eleginoides* Smitt, 1898	JX188801	JN187039	JX189902	DQ498780	JN186863	JN186957	JN186998	JN186821
**7**	*Dissostichus mawsoni* Norman, 1937	Dornb2017	JN187040	Dornb2017	DQ498794	AY517753	JN186958	JN186999	JN186822
**8**	*Lindbergichthys nudifrons* (Lönnberg, 1905)	HM166387	HM166362	HM166287	HM166262	JN186868	HM166237	HM166212	HM166187
**9**	*Trematomus borchgrevinki* (Boulenger, 1902)	HM166389	HM166365	HM166290	HM166264	FJ647674	HM166239	HM166214	HM166189
**10**	*Trematomus eulepidotus* Regan, 1914	HM166393	HM166368	HM166293	JQ063287	FJ647680	HM166243	HM166218	HM166193
**11**	*Trematomus tokarevi* Andriashev, 1978	HM166409	HM166384	HM166310	HM166284	FJ647710	HM166259	HM166234	HM166209
**12**	*Notothenia coriiceps* Richardson, 1844	Dornb2017	JN187047	Dornb2017	AY141302	FJ647673	JN186965	JN187006	JN186829
**13**	*Artedidraco skottsbergi* Lönnberg, 1905	Dornb2017	JN187027	Dornb2017	JF292779	JN186850	JN186945	JN186986	JN186809
**14**	*Dolloidraco longedorsalis* Roule, 1913	HQ169733	HQ169792	HQ169973	JF292796	FJ647619	HQ170179	HQ170238	HQ169672
**15**	*Harpagifer antarcticus* Nybelin, 1947	HQ169732	JN187037	HQ169972	HQ170033	JN186861	JN186955	JN186996	JN186819
**16**	*Akarotaxis nudiceps* (Waite, 1916)	HQ169765	HQ169824	HQ170005	HQ170067	HQ170152	HQ170211	HQ170270	HQ169704
**17**	*Racovitzia glacialis* Dollo, 1900	HQ169789	HQ169848	HQ170029	HQ170090	HQ170174	HQ170235	HQ170294	HQ169728
**18**	*Gerlachea australis* Dollo, 1900	HQ169776	HQ169835	HQ170016	HQ170077	HQ170162	HQ170222	HQ170281	HQ169715
**19**	*Gymnodraco acuticeps* Boulenger, 1902	HQ169778	HQ169838	HQ170019	HQ170080	HQ170165	HQ170225	HQ170284	HQ169718
**20**	*Champsocephalus gunnari* Lönnberg, 1905	HQ169740	HQ169799	HQ169980	HQ170042	HQ170137	HQ170186	HQ170245	HQ169679
**21**	*Chaenocephalus aceratus* (Lönnberg, 1906)	HQ169734	HQ169793	HQ169974	HQ170035	HM166091	HQ170180	HQ170239	HQ169673
**22**	*Chaenodraco wilsoni* Regan, 1914	HQ169736	HQ169795	HQ169976	HQ170037	HM165755	HQ170182	HQ170241	HQ169675
**23**	*Chionodraco hamatus* (Lönnberg, 1905)	HQ169746	HQ169804	HQ169985	DQ498792	HQ170142	HQ170191	HQ170250	HQ169684
**24**	*Chionodraco myersi* DeWitt & Tyler, 1960	HQ169747	HQ169807	HQ169988	HQ170048	HQ170145	HQ170194	HQ170253	HQ169687
**25**	*Chionodraco rastrospinosus* DeWitt & Hureau, 1979	HQ169749	HQ169808	HQ169989	HQ170050	HM165858	HQ170195	KC830288	HQ169688
**26**	*Neopagetopsis ionah* Nybelin, 1947	HQ169757	HQ169817	HQ169998	HQ170058	HM165690	HQ170204	HQ170263	HQ169697
**27**	*Pagetopsis macropterus* (Boulenger, 1907)	HQ169759	HQ169818	HQ169999	HQ170060	HM165520	HQ170205	HQ170264	HQ169698
**28**	*Pseudochaenichthys georgianus* Norman, 1937	HQ169763	HQ169823	HQ170003	HQ170064	HM165578	HQ170210	HQ170269	HQ169702

***, not available,

*KF139693, *Bovichtus diacanthus*; JN187019, *Bovichtus variegatus*; JN187020 *Bovichtus diacanthus*; Dornb2017 *Bovichtus variegatus*; KF141190, *Bovichtus diacanthus*; AY141299 *Bovichtus variegatus*; JN186842 *Bovichtus variegatus*; JN186843 *Bovichtus diacanthus*; JN186937 *Bovichtus variegatus*; JN186938 *Bovichtus diacanthus*; JN186978, *Bovichtus variegatus*; JN186979 *Bovichtus diacanthus*; JN186801 *Bovichtus variegatus*; JN186802 *Bovichtus diacanthus*; Dornb2017, available through Dornburg et al. (2017).

Reference to Supplementary Table S3

Dornburg A, Federman S, Lamb AD, Jones CD, Near TJ. 2017. Cradles and museums of Antarctic teleost biodiversity. *Nat Ecol Evol.* 1:1379-1384.

Supplementary Table S4. List of primers used to amplify and sequence the complete mitochondrial genomes of *Trematomus borchgrevinki* and *Aethotaxis mitopteryx* were obtained by long PCR. The table reports: the name of each primer, the 5'-3' sequence and the melting temperature (Tm).

**Primer name**	**Primer sequence 5'-3'**	***Tm***
AetH789	ACCACAAACTCTTGCCTAAACATTG	*55°C*
AetH920	CCCCGAAGCACTGTCTAGCACGT	*55°C*
AetH1649	CGGGACACTTCCAGAGCAACA	*55°C*
AetH2006	GATAAACCCACACCCCATTGCC	*55°C*
AetLDl521	GTGGTCGGGTTACCCTCTAAGC	*55°C*
L7061Tre	TGGCCATGTGGTTGGCTTGAAAC	*60°C*
H5231Tre	TAGACAGGYAGGCTTCGAYCCTACA	*60°C*
L11944Tre	CATAGCTACTACTTGGATTTGCACCA	*60°C*
H11910Tre	CAGCTCATCCATTGGTCTTAGGAAC	*60°C*
L15045thr	GGTTTAACCTCCGACCTCCG	*60°C*
IleH13957	TGATCCTTTATTTCAGGCACAACTC	*60°C*
Nd1LeuTre	GTTCAAGTCCTCTCCTTAACTATGC	*60°C*

Supplementary Table S5. Best partition scheme selected for *28T.Mito* + *28T.Nucl* datasets

```
#NEXUS

begin sets;

  charset part1 = genes.mit.nex: 1-678  5698-6738  6739-7083  7084-8460  8461-8751  8752-10587;
  charset part2 = genes.mit.nex: 679-849;
  charset part3 = genes.mit.nex: 850-1986  1987-3261  3262-3948  3949-4728  4729-5697;
  charset part4 = genes.mit.nex: 10588-11106;
  charset part5 = genes.mit.nex: 11107-12829  12830-13791;
  charset part6 = genes.nuc.nex: 1-864  865-1590  1591-2895  4424-5128  5765-6619;
  charset part7 = genes.nuc.nex: 2896-3681  5129-5764;
  charset part8 = genes.nuc.nex: 3682-4423;

  charpartition mine = TIM3+F+I+G4: part1, K2P+G4: part2, TIM3+F+I+G4: part3, TIM+F+G4: part4, TIM2+F+I+G4: part5, TN+F+I+G4: part6,
HKY+F+I+G4: part7, HKY+F+G4: part8;
end;
```

Notes

genes.mit.nex = *28T.Mito* data set; genes.nuc.nex = *28T.Nucl* data set

genes.mit.nex; gene arrangement

GENE	START	END	INITIAL	SIZE	FINAL
atp6	1	678	part1	678	part1
atp8	679	849	part2	171	part2
cob	850	1986	part3	1137	part3
cox1	1987	3261	part4	1275	part3
cox2	3262	3948	part5	687	part3
cox3	3949	4728	part6	780	part3
nad1	4729	5697	part7	969	part3
nad2	5698	6738	part8	1041	part1
nad3	6739	7083	part9	345	part1
nad4	7084	8460	part10	1377	part1
nad4L	8461	8751	part11	291	part1
nad5	8752	10587	part12	1836	part1
nad6	10588	11106	part13	519	part4
rrnL	11107	12829	part14	1723	part5
rrnS	12830	13791	part15	962	part5

genes.nuc.nex; gene arrangement

GENE	START	END	INITIAL	SIZE	FINAL
glyt	1	864	part1	864	part6
myh6	865	1590	part2	726	part6
rag1	1591	2895	part3	1305	part6
Rhod	2896	3681	part4	786	part7
S7i	3682	4423	part5	742	part8
SH3PX3	4424	5128	part6	705	part6
tbr1	5129	5764	part7	636	part7
zic1	5765	6619	part8	855	part6

INITIAL = starting partition number; FINAL = final partition assignment provided by IqTree.

Supplementary Table S6a. Summary of the events of episodic diversifying selection (green boxes) for each mitochondrial protein-coding gene at different taxonomic scale in the Notothenioidei as detected with aBSREL.

	**Taxon**												
**Gene**	**Nototheniidae**	***Pleuragramma antarctica***	**Dissostichinae**	***Aethotaxis mitopteryx***	***Dissostichus* genus**	**Trematominae**	***Notothenia coriiceps***	***Harpagifer antarcticus***	**Artedidraconinae**	**Bathydraconinae**	***Gymnodracro acuticeps***	**Channichthyinae**	***Champsocephalus gunnari***
cox1													
cox2													
cox3						■							
atp6													
atp8													
cob													
nad1													
nad2													
nad3										■			
nad4													
nad4L													
nad5												■	
nad6						■							

Supplementary Table S6b. Occurrence of the events of episodic diversifying selection in the protein-encoding mitochondrial genes of Notothenioidei and associated statistics (aBSREL software).

**Gene**	**Taxon**	**LRT**	**p-value**	**ω distribution over sites**	
cox3	**Trematominae, branch leading to node 18**	10.4663	0.0129	ω1 = 0.00 (99%)	ω2 = 62.2 (0.97%)
nad3	***Racovitzia glacialis***	6.1906	0.0484	ω1 = 0.165 (99%)	ω2 = 100000 (0.95%)
nad5	**Channichthyinae root, branch leading to node 37**	338.759	0.0000	ω1 = 0.238 (96%)	ω2 = 90.8 (3.8%)
nad6	***Trematomus* genus root, branch leading to node 16**	20.4080	0.0001	ω1 = 0.191 (97%)	ω2 = 120 (3.1%)

LRT, Likelihood ratio test.

Supplementary Table S7a. Summary of the events of intensification or relaxation (blue and red boxes respectively) for each mitochondrial protein-coding gene at different taxonomic scale in the Notothenioidei as detected with RELAX.

	Taxon												
**Gene**	**Nototheniidae**	***Pleuragramma antarctica***	**Dissostichinae**	***Aethotaxis mitopteryx***	***Dissostichus* genus**	**Trematominae**	***Notothenia coriiceps***	***Harpagifer antarcticus***	**Artedidraconinae**	**Bathydraconinae**	***Gymnodracro acuticeps***	**Channichthyinae**	***Champsocephalus gunnari***
cox1													
cox2												blue	
cox3	red	red				blue						blue	
atp6						red						blue	
atp8										blue			
cob	red		red	red								blue	
nad1			red		red								
nad2	red	red				red	blue		blue		red	blue	blue
nad3			red		red								
nad4L						blue							
nad4	red		red		red	blue				red		blue	blue
nad5							red					blue	blue
nad6	red		red		red		blue	red			red	blue	

event of **intensification** of selection identified by RELAX (blue)

event of **relaxation** of selection identified by RELAX (red)

Supplementary Table S7b. Statistics related to the events of intensification or relaxation of selection (blue/red boxes) in the mitochondrial protein coding genes of Notothenioidei and generated with RELAX.

gene	I/R	result	Taxon	gene	I/R	result	Taxon
cox2		Test for selection intensification (K = 1.56) was significant (p = 0.038, LR = 4.29).	Channichthyinae	*nad3*		Test for selection relaxation (K = 0.01) was significant (p = 0.049, LR = 3.86).	Dissostichinae
				nad3		Test for selection relaxation (K = 0.01) was significant (p = 0.031, LR = 4.65).	*Dissostichus* genus
cox3		Test for selection relaxation (K = 0.35) was significant (p = 0.023, LR = 5.19).	Nototheniidae				
cox3		Test for selection relaxation (K = 0.05) was significant (p = 0.003, LR = 8.64).	*Pleuragramma antarctica*	*nad4L*		Test for selection intensification (K = 20.43) was significant (p = 0.032, LR = 4.59).	Trematominae
cox3		Test for selection intensification (K = 6.41) was significant (p = 0.025, LR = 5.00).	Trematominae				
cox3		Test for selection intensification (K = 4.63) was significant (p = 0.006, LR = 7.55).	Channichthyinae	*nad4*		Test for selection relaxation (K = 0.77) was significant (p = 0.007, LR = 7.18).	Nototheniidae
				nad4		Test for selection relaxation (K = 0.51) was significant (p = 0.000, LR = 49.55).	Dissostichinae
atp6		Test for selection relaxation (K = 0.73) was significant (p = 0.013, LR = 6.19).	Trematominae	*nad4*		Test for selection relaxation (K = 0.26) was significant (p = 0.000, LR = 69.47).	*Dissostichus* genus
atp6		Test for selection intensification (K = 2.09) was significant (p = 0.020, LR = 5.37).	Channichthyinae	*nad4*		Test for selection intensification (K = 1.78) was significant (p = 0.005, LR = 7.84).	Trematominae
				nad4		Test for selection relaxation (K = 0.67) was significant (p = 0.012, LR = 6.29).	Bathydraconinae
atp8		Test for selection intensification (K = 2.83) was significant (p = 0.030, LR = 4.69).	Bathydraconinae	*nad4*		Test for selection intensification (K = 12.23) was significant (p = 0.000, LR = 19.34).	Channichthyinae
				nad4		Test for selection intensification (K = 17.04) was significant (p = 0.003, LR = 8.86).	*Champsocephalus gunnari*
cob		Test for selection relaxation (K = 0.40) was significant (p = 0.032, LR = 4.57).	Nototheniidae				
cob		Test for selection relaxation (K = 0.70) was significant (p = 0.020, LR = 5.38).	Dissostichinae	*nad5*		Test for selection relaxation (K = 0.08) was significant (p = 0.000, LR = 14.49).	*Notothenia coriiceps*
cob		Test for selection relaxation (K = 0.64) was significant (p = 0.019, LR = 5.48).	*Aethotaxis mitopteryx*	*nad5*		Test for selection intensification (K = 3.26) was significant (p = 0.031, LR = 4.67).	Channichthyinae
cob		Test for selection intensification (K = 4.35) was significant (p = 0.000, LR = 18.05).	Channichthyinae	*nad5*		Test for selection intensification (K = 2.12) was significant (p = 0.001, LR = 11.66).	*Champsocephalus gunnari*
nad1		Test for selection relaxation (K = 0.77) was significant (p = 0.028, LR = 4.83).	Dissostichinae	*nad6*		Test for selection relaxation (K = 0.67) was significant (p = 0.009, LR = 6.79).	Nototheniidae
nad1		Test for selection relaxation (K = 0.68) was significant (p = 0.006, LR = 7.44).	*Dissostichus* genus	*nad6*		Test for selection relaxation (K = 0.51) was significant (p = 0.002, LR = 9.47).	Dissostichinae
				nad6		Test for selection relaxation (K = 0.48) was significant (p = 0.005, LR = 8.05).	*Dissostichus* genus
nad2		Test for selection relaxation (K = 0.74) was significant (p = 0.027, LR = 4.92).	Nototheniidae	*nad6*		Test for selection intensification (K = 3.18) was significant (p = 0.005, LR = 7.72).	*Notothenia coriiceps*
nad2		Test for selection relaxation (K = 0.65) was significant (p = 0.019, LR = 5.50).	*Pleuragramma antarctica*	*nad6*		Test for selection relaxation (K = 0.43) was significant (p = 0.010, LR = 6.56).	*Harpagifer antarcticus*
nad2		Test for selection relaxation (K = 0.82) was significant (p = 0.019, LR = 5.55).	Trematominae	*nad6*		Test for selection relaxation (K = 0.38) was significant (p = 0.000, LR = 13.65).	*Gymnodracro acuticeps*
nad2		Test for selection intensification (K = 1.67) was significant (p = 0.017, LR = 5.74).	*Notothenia coriiceps*	*nad6*		Test for selection intensification (K = 2.80) was significant (p = 0.000, LR = 16.39).	Channichthyinae
nad2		Test for selection intensification (K = 1.73) was significant (p = 0.033, LR = 4.53).	Artedidraconinae				
nad2		Test for selection relaxation (K = 0.21) was significant (p = 0.000, LR = 27.68).	*Gymnodracro acuticeps*			**Channichthyinae; *nad5*: selected branches**	
nad2		Test for selection intensification (K = 1.49) was significant (p = 0.000, LR = 14.29).	Channichthyinae	*nad5*		Test for selection relaxation (K = 0.11) was significant (p = 0.000, LR = 16.78).	root, branch leading to node 37
nad2		Test for selection intensification (K = 1.58) was significant (p = 0.028, LR = 4.84).	*Champsocephalus gunnari*	*nad5*		Test for selection intensification (K = 7.22) was significant (p = 0.000, LR = 26.22).	branch leading to node 39 and all branches emerging from it

I/R, Intensification/Relaxation of the selection; **K**, the *selection intensity parameter*; **p**, p-value; **LR**, Likelihood ratio test.
